# Supplementary material for: DNA T-shaped crossover tiles for 2D tessellation and nanoring reconfiguration
Source: Nat Commun. 2023 Nov 23;14:7675. doi: 10.1038/s41467-023-43558-8 (PMC10667507; doi:10.1038/s41467-023-43558-8)
Supplement: Supplementary file 1 — Supplementary information [file 41467_2023_43558_MOESM1_ESM.pdf]

Supplementary Information

DNA T-Shaped Crossover Tiles for 2D Tessellation and Nanoring Reconfiguration

Qi Yang[a], Xu Chang[a], Jung Yeon Lee[a], Minu Saji[a], Fei Zhang[a]*

[a]Department of Chemistry, Rutgers University, Newark, NJ 07102, USA

*Address correspondence to this author at the Department of Chemistry, Rutgers University, Newark, NJ 07102, USA; Tel: 973-353-5520; E-mails: fei.zhang@rutgers.edu

Inventory of Supplementary Information:

Section 1 Supplementary Methods **1-2**

Section 2 Supplementary Figures...**3-64**

Section 3 Supplementary notes...**65-87**

Section 4 Supplementary Tables...**88-94**

Section 5 Supplementary References...**94**

Section 6 Source data of Supplementary information...**94-102**

Section 1 Supplementary Methods

Annealing program for the assembly of nanostructures

For the assembly of ladders and 2D arrays, 2 h, 16 h and 66 h annealing programs were used.

2 h program: 94 °C for 2 minutes; 92 °C to 76 °C at 2 °C per 5 minutes; 72 °C to 24 °C at 4 °C per 5 minutes; then hold at 4 °C.

16 h program: 94 °C for 5 minutes; 90 °C for 5 minutes; 86°C to 77 °C at 1°C per 5 minutes; 76°C to 70°C at 1 °C per 10 minutes; 69 °C to 37 °C at 1 °C per 15 minutes; 36 °C to 20 °C at 1 °C per 20 minutes; then hold at 4°C.

66 h program: 94 °C for 5 minutes; 90 °C for 5 minutes; 86 °C to 76 °C at 1 °C per 5 minutes; 75°C to 71 °C at 1°C per 30 minutes; 70 °C to 61 °C at 1°C per 40 minutes; 60 °C to 46 °C at 1 °C per 50 minutes; 45°C to 38 °C at 1°C per 60 minutes; 37 °C to 20 °C at 1°C per 70 minutes; 32°C to 27 °C at 1°C per 30 minutes; 26 °C to 15 °C at 1°C per 60 minutes; then hold at 4°C.

For dynamic ring assembly (2 h program): 95 °C for 5 minutes; 65 °C for 30 minutes; 50 °C for 30 minutes; 37 °C for 30 minutes; 22 °C for 30 minutes; then hold at 22 °C.

Calculation of the Hybridization energy of sticky ends

The Nearest-neighbor thermodynamic parameters for DNA Watson-Crick pairs in 1 M NaCl [1]. The calculation formula and parameters are shown below:

$$\Delta G = \Delta G_{initiation} + \Delta G_{symmetry} + \sum \Delta G_{stacking} + \Delta G_{ATterm}$$

The $\Delta G_{initiation}$ is +1.96 kcal mol^{-1}. The $\Delta G_{symmetry}$ correction is +0.43 kcal mol^{-1}, which applies to only self-complementary sticky ends. The terminal AT penalty (ΔG_{ATterm}) is applied for each end of a sticky end pair that has a terminal AT (a sticky end duplex with one end closed by AT pairs would have a penalty of 0.05 kcal mol^{-1}, or both ends closed by AT pairs have a penalty of 0.1 kcal mol^{-1}). For the calculation of $\Sigma\Delta G_{stacking}$ please see references 1 for further explanation. The hybridization energy of sticky ends of each design is summarized in Supplementary Table 2-12.

Transmission electron microscopy (TEM)

TEM images were acquired on a JEOL 1200EX electron microscope with AMT-XR41 digital camera. For the sample preparation, 1 μL of annealed DNA solution was diluted into 15 μL stock solution and mixed gently with pipet by 20 times up and down pipetting. A drop of the stock solution (5 μL) was deposited on a glow-discharged carbon coated copper grid (Electron Microscopy Sciences), incubated for 3 min, and blotted away the remained solution by lightly touching an edge or corner of filter paper to the edge of the grid. The grid was stained with 5 μL 0.75 % (w/v) uranyl formate for 1 min. Samples were air dried before examination in the TEM.

Toehold-mediated strand displacement reaction (SDR)

The dynamic transition (or reaction) of both the SST nanoring and single monomer nanoring are achieved by SDR. Here, the core idea was manipulating the length of one or two horizontal arms by SDR and at the same time retaining the length of the supporting arm. For example, for two states transformation of a C tile based single monomer nanoring, the switching between state 1 to state 2 was achieved by tuning the length of both horizontal arms by SDR simultaneously and keeping the *N* value and the width of the nanoring unchanged (Supplementary Fig 34). In this process the only thing that changed is the diameter of the ring. Two 8 nt toeholds are anchored to upper and lower horizontal arms of TC-C-9-3.5-6, respectively. Before transition, the top and bottom horizontal arms of TC-C-9-3.5-6 (state 1) are 9 turns and 6 turns, respectively. After adding the invader strand 1 and 2, the invader strand binds the toehold and displaces the two complementary strands from the state 1 structure, leaving an intermediate with a partially single

stranded horizontal arm. Notably, a stem loop structure is designed within this single strand region to reduce the length of the arm. Then the setting strand 1 and 2 are added to the system, hybridize to the remaining single strand region of the horizontal arms producing the state 2 structure (TC-C-7-3.5-4). This process can be reversed in two steps by adding invader strands (3, 4) and setting strands (3, 4), to reverse states 2 to states 1.

Similarly, for the four states transformation, from TC-C-9-3.5-6 to TC-C-7-3.5-6, invader strand 1 and setting strand 1 were used. From TC-C-7-3.5-6 to TC-C-7-3.5-4, invader strand 2 and setting strand 2 were used. For information about the rest of invader strands and setting strands please refer to Supplementary Fig 44. Four groups of nanoring dynamic system shared the same invader strands and setting strands pairs as each transition case.

The calculation of number of monomer tiles in one nanoring.

The number of monomers in a DNA nanoring can be calculated based on the length of upper horizontal arm (*L*2), vertical arm (*R*), and lower horizontal arm (*L*1) of C-shape or Z-shape TC tiles. Here, *C* is the circumference of the inner circle of ring shape. *N* is the calculated number of monomers in one nanoring. The calculation of monomer numbers (*N* value) is as below.

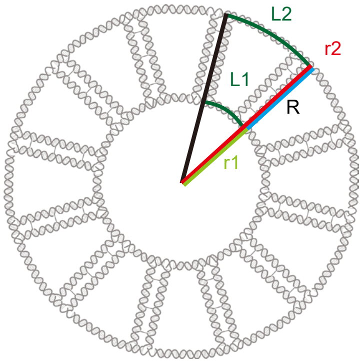

Step 1: *r*2 = *r*2+*R*

Step 2: *L*2/*L*1 = *r*2/*r*1 = (*r*1+*R*)/*r*1

Step 3: *R*1 = *L*1**R*/(*L*2-*L*1)

Step 4: *C*1 = 2*π*1 = 2*π**L*1**R*/(*L*2-*L*1)

Step 5: *N* = *C*1/*L*1

The calculated N value of four pairs of 4-state monomers are summarized in Supplementary table 1.

Yield calculation of the Nanoring

The statistics of nanoring yield were done by hand counting. For each sample, at least 260 assembled structures were counted. Here is an example to show the yield calculations of 12-monomer rings:

$$yield_{N=12} = \frac{number\ of\ rings\ (N = 12)\ \times\ 12\ tiles}{Total\ number\ of\ C\ tiles}$$

In this equation, the number of rings (*N* = 12) was counted from AFM images. The Total number of C tiles was estimated from the sum of all the sized rings and unassembled fragments. For the detailed yield calculations of each sample see Supplementary figures and Supplementary Excel file.

Section 2 Supplementary Figures

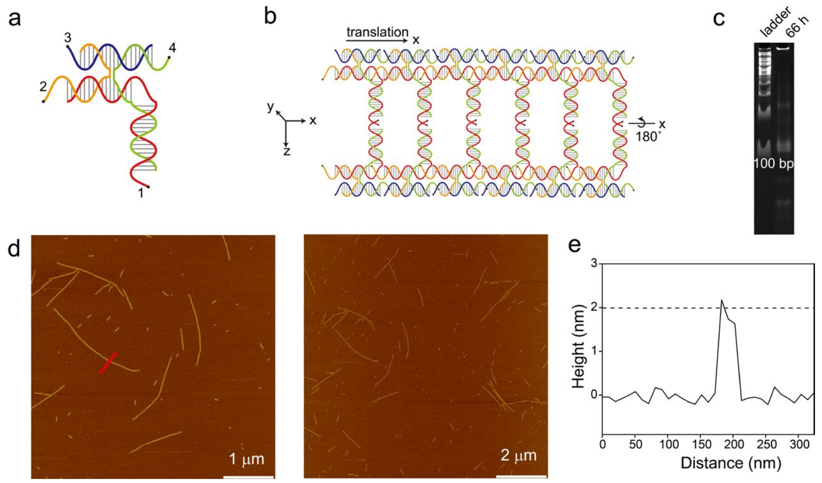

Supplementary Figure 1 | Schematics and characterization of the design of TC-1-1. a) The structural design of TC-1-1. b) Sticky ends matching rules. c) Native gel images. d) AFM images (66 h annealing program). e) The height measurement of TC-1-1. Notes, all the height data were analyzed by Bruker NanoScope Analysis 2.0. Black dots marked in tiles schematics represent the 5' primer of the strands. The red line labeled in AFM images shows the height data extract location. Source data are provided as a Source Data file.

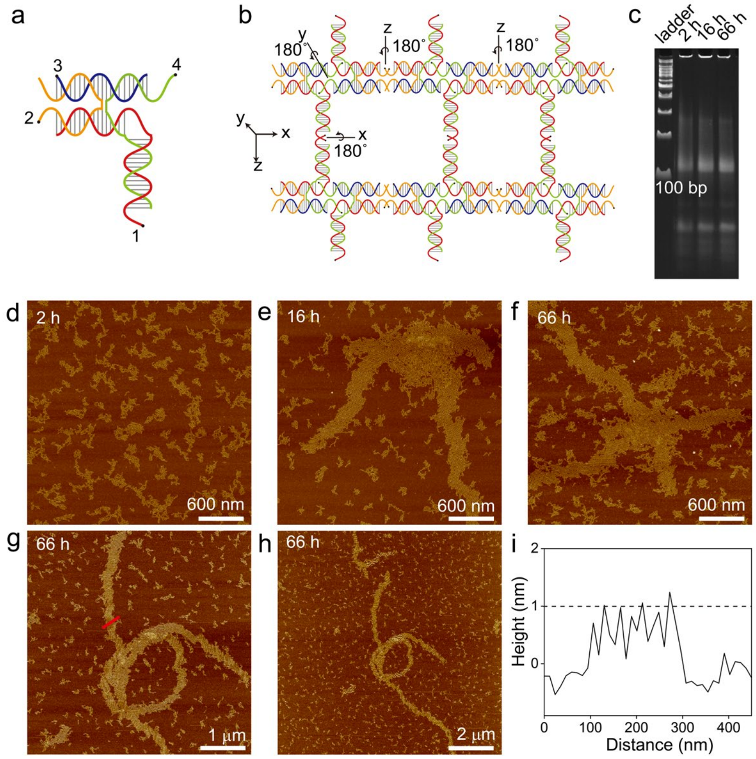

Supplementary Figure 2 | Schematics and characterization of the design of TC-1-2. a) The structural design of TC-1-2. b) Sticky ends matching rules. c) Native gel images of TC-1-2. d-h) AFM images (2 h, 16 h, 66 h annealing program). i) Height measurement of TC-1-2. The red line labeled in AFM images shows the height data extract location. Source data are provided as a Source Data file.

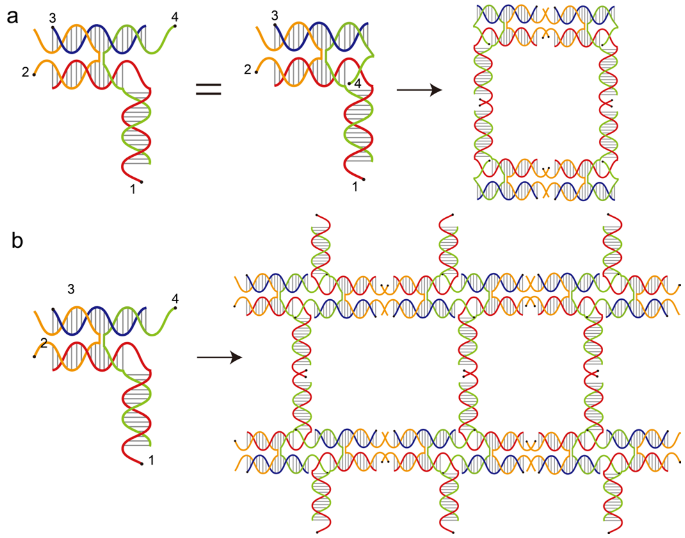

Supplementary Figure 3 | The possible intramolecular binding assembled structure of TC-1-2, a) intramolecular binding, a defined rectangular structure; b) intermolecular binding, a grid.

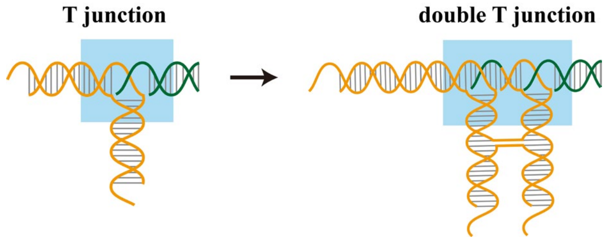

Supplementary Figure 4 | Structural design of double T junction. The double T junction is two T junctions overlapping with each other. The T junction and double T junction are highlighted with light blue color**.**

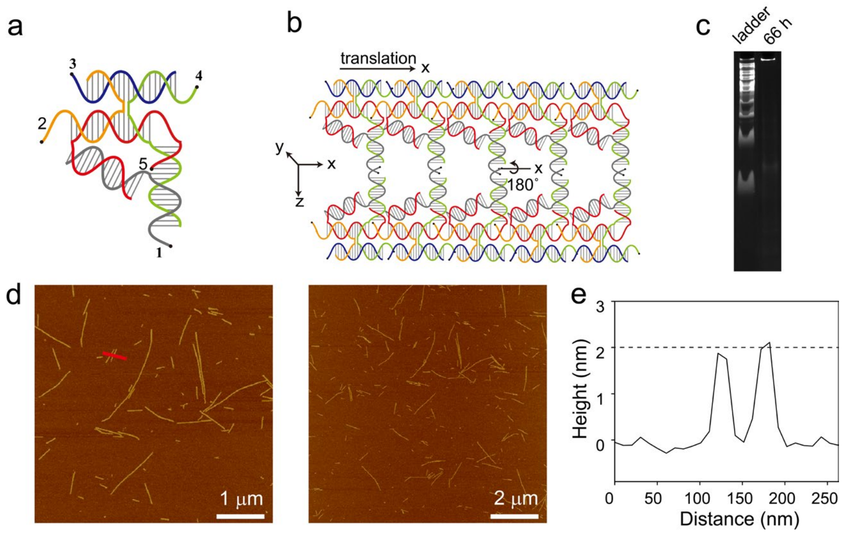

Supplementary Figure 5 | Schematics and characterization of the design of TC-2-1. a) The structural design of TC-2-1. b) Sticky ends matching rules. c) Native gel images of TC-2-1. d) AFM images (66 h annealing program). e) Height measurement of TC-2-1. The red line labeled in AFM images shows the height data extract location. Source data are provided as a Source Data file.

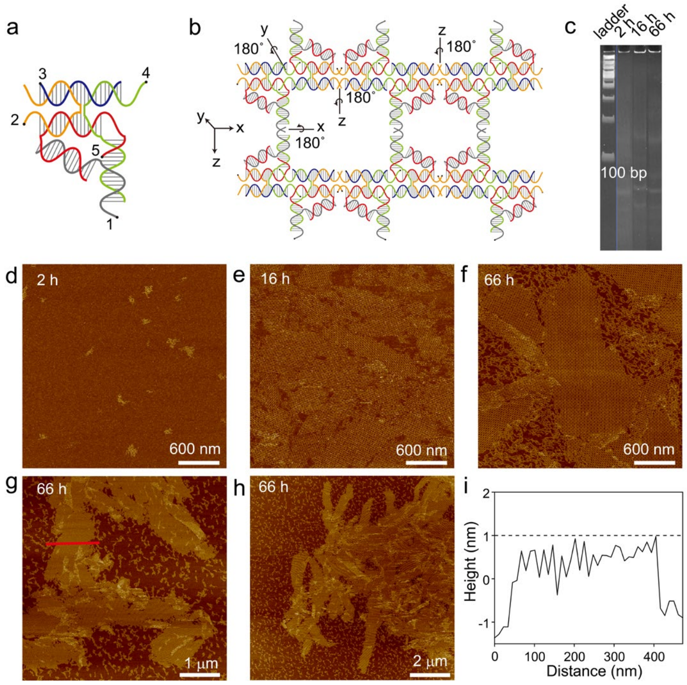

Supplementary Figure 6 | Schematics and characterization of the design of TC-2-2. a) The structural design of TC-2-2. b) Sticky ends matching rules. c) Native gel images of TC-2-2. d-h) AFM images (2 h, 16 h, 66 h annealing program). i) Height measurement of TC-2-2. The red line labeled in AFM images shows the height data extract location. Source data are provided as a Source Data file.

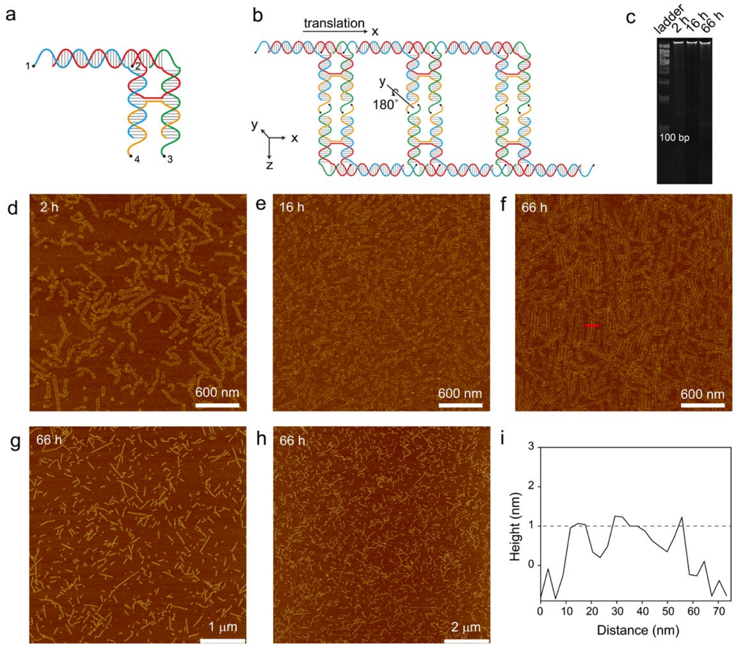

Supplementary Figure 7 | Schematics and characterization of the design of TC-3-1 a) The structural design of TC-3-1. b) Sticky ends matching rules. c) Native gel images of TC-3-1. d-h) AFM images (2 h, 16 h, 66 h annealing program). i) Height measurement of TC-3-1. The red line labeled in AFM images shows the height data extract location. Source data are provided as a Source Data file.

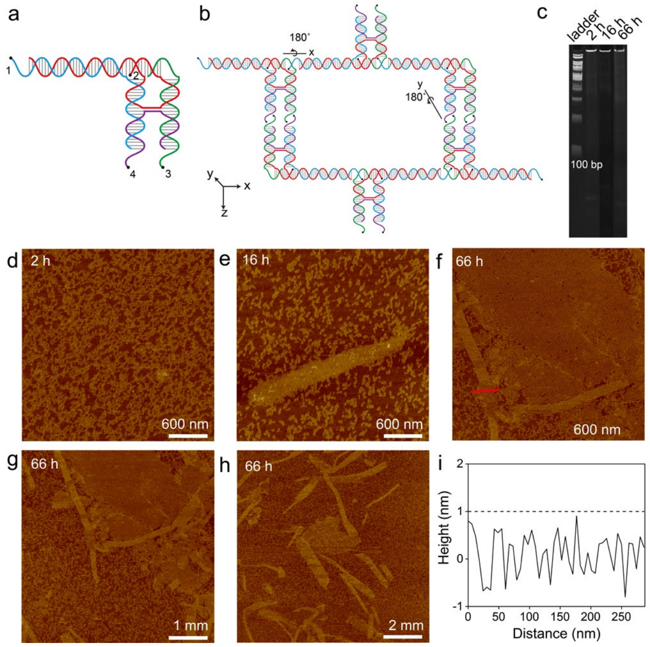

Supplementary Figure 8 | Schematics and characterization of the design of TC-3-2. a) The structural design of TC-3-2. b) Sticky ends matching rules. c) Native gel images of TC-3-2. d-h) AFM images (2 h, 16 h, 66 h annealing program). i) Height measurement of TC-3-2. The red line shows the height data extract location. Source data are provided as a Source Data file.

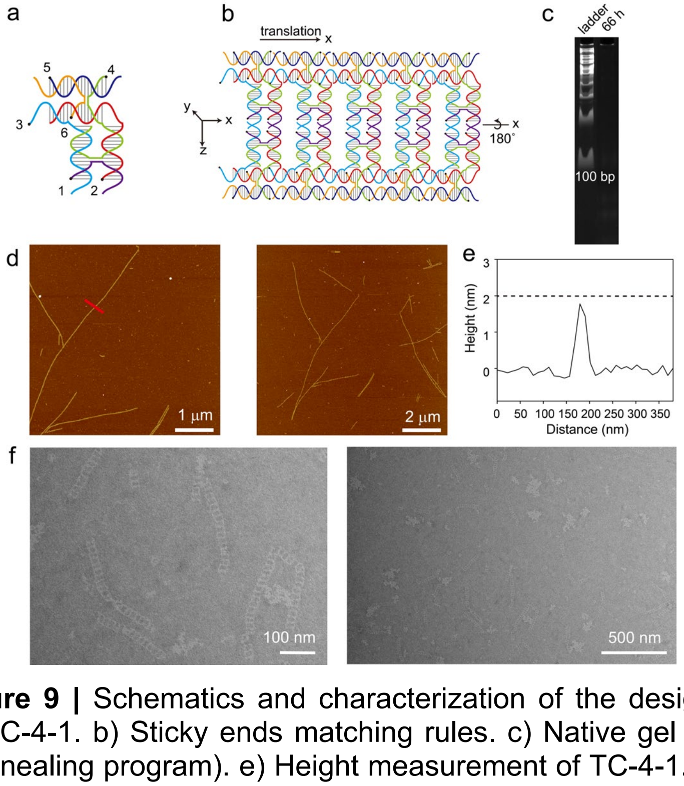

Supplementary Figure 9 | Schematics and characterization of the design of TC-4-1. a) The structural design of TC-4-1. b) Sticky ends matching rules. c) Native gel images of TC-4-1. d) AFM images (66 h annealing program). e) Height measurement of TC-4-1. f) TEM images (66 h annealing program) of TC-4-1. The red line shows the height data extract location. Source data are provided as a Source Data file.

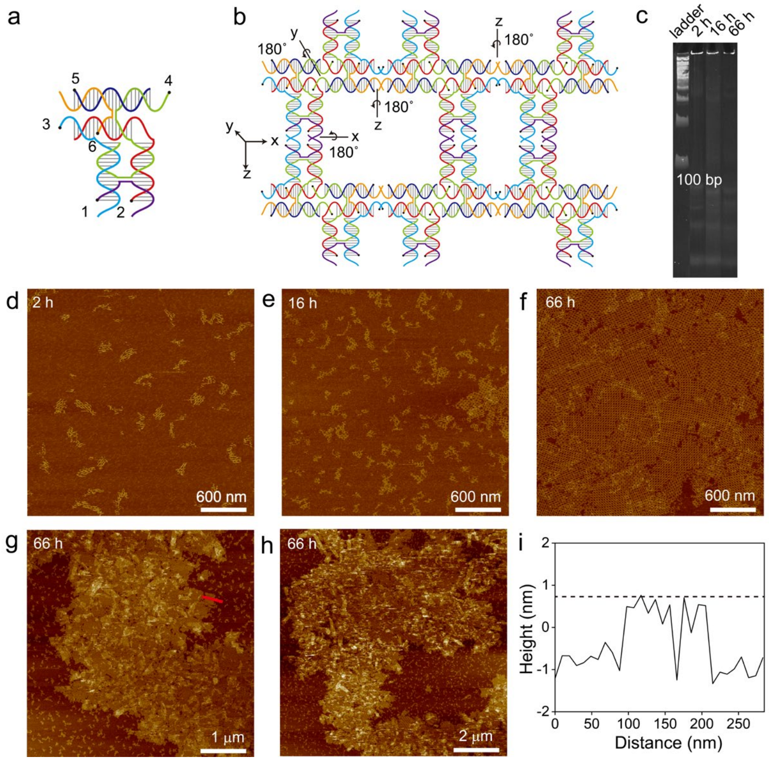

Supplementary Figure 10 | Schematics and characterization of the design of TC-4-2. a) The structural design of TC-4-2. b) Sticky ends matching rules. c) Native gel images of TC-4-2. d-h) AFM images (2 h, 16 h, 66 h annealing program). i) Height measurement of TC-4-2. The red line labeled in AFM images shows the height data extract location. Source data are provided as a Source Data file.

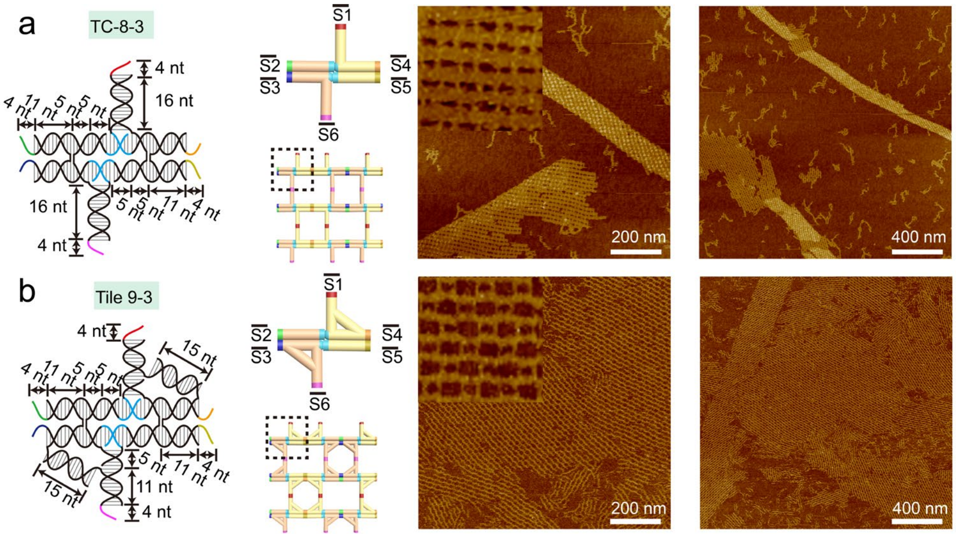

Supplementary Figure 11 | Schematics and characterization of the design of TC-8-3 and TC-9-3. The structure design, Sticky ends matching rules, and AFM images (66 h annealing program) of a) TC-8-3 and b) TC-9-3.

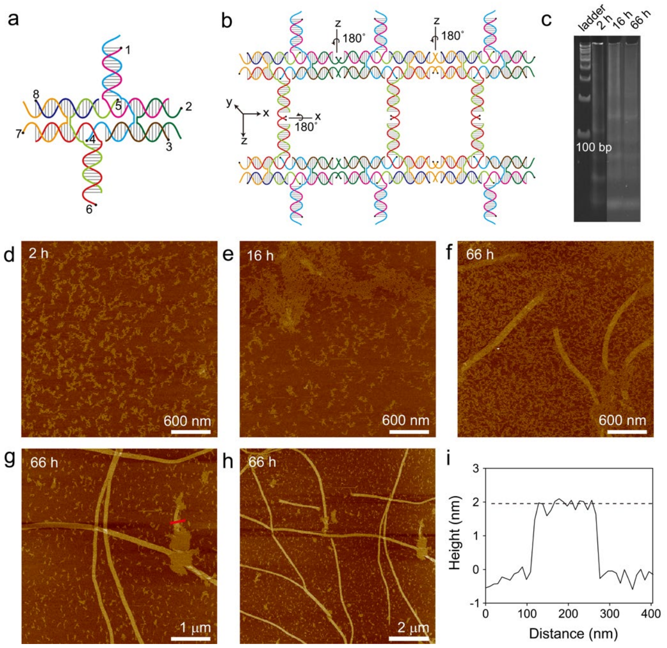

Supplementary Figure 12 | Schematics and characterization of the design of TC-8-3. a) The structural design of TC-8-3. b) Sticky ends matching rules. c) Native gel images of TC-8-3. d-h) AFM images (2 h, 16 h, 66 h annealing program). i) Height measurement of TC-8-3. The red line labeled in AFM images shows the height data extract location. Source data are provided as a Source Data file.

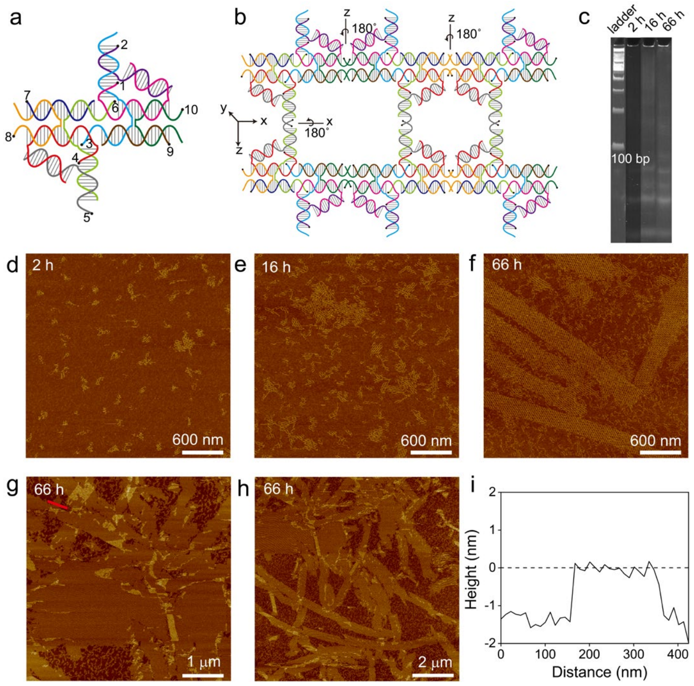

Supplementary Figure 13 | Schematics and characterization of the design of TC-9-3. a) The structural design of TC-9-3. b) Sticky ends matching rules. c) Native gel images of TC-9-3. d-h) AFM images (2 h, 16 h, 66 h annealing program). i) Height measurement of TC-9-3. The red line shows the height data extract location. Source data are provided as a Source Data file.

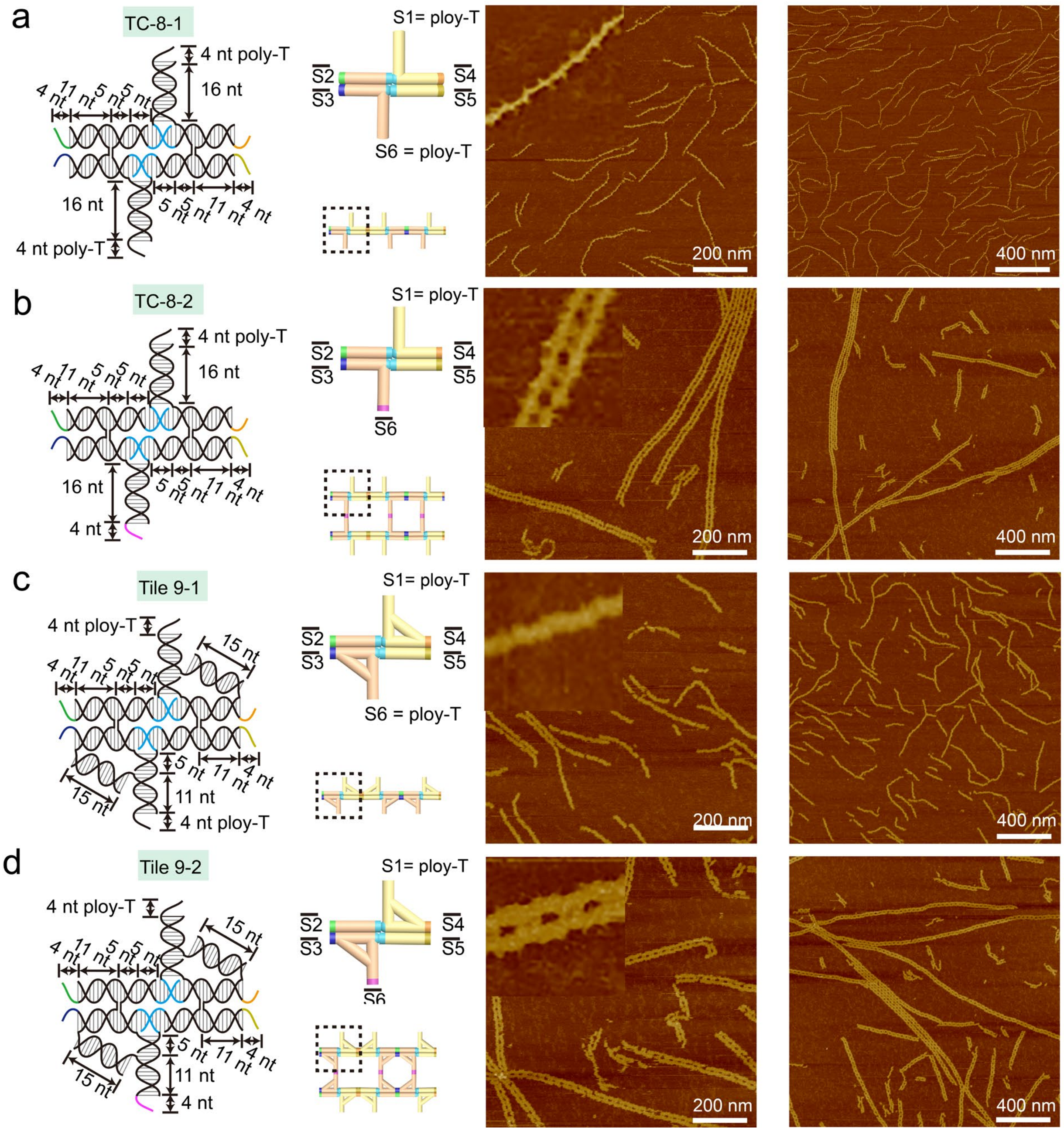

Supplementary Figure 14 | Schematics and characterization of the design of TC-8-1, TC-8-2, TC-9-1 and TC-9-2. The structural design, sticky ends matching rules, and AFM images (66 h annealing program) of a) TC-8-1, b) TC-8-2, c) TC-9-1 and d) TC-9-2.

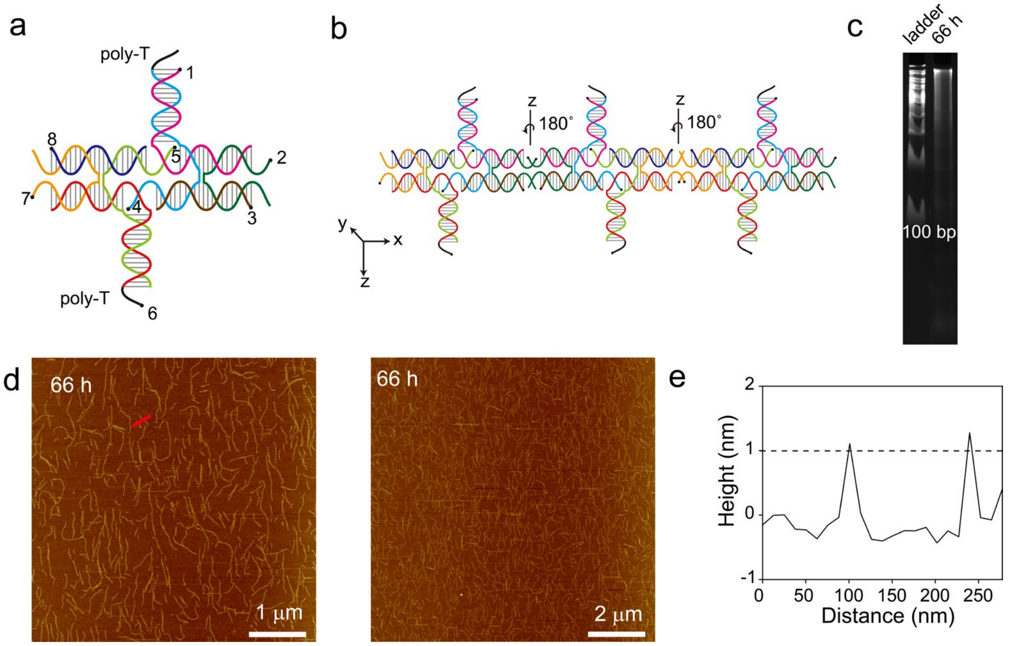

Supplementary Figure 15 | Schematics and characterization of the design of TC-8-1. a) The structural design of TC-8-1. b) Sticky ends matching rules. c) Native gel images of TC-8-1. d) AFM images (66 h annealing program). e) Height measurement of TC-8-1. The red line labeled in AFM images shows the height data extract location. Source data are provided as a Source Data file.

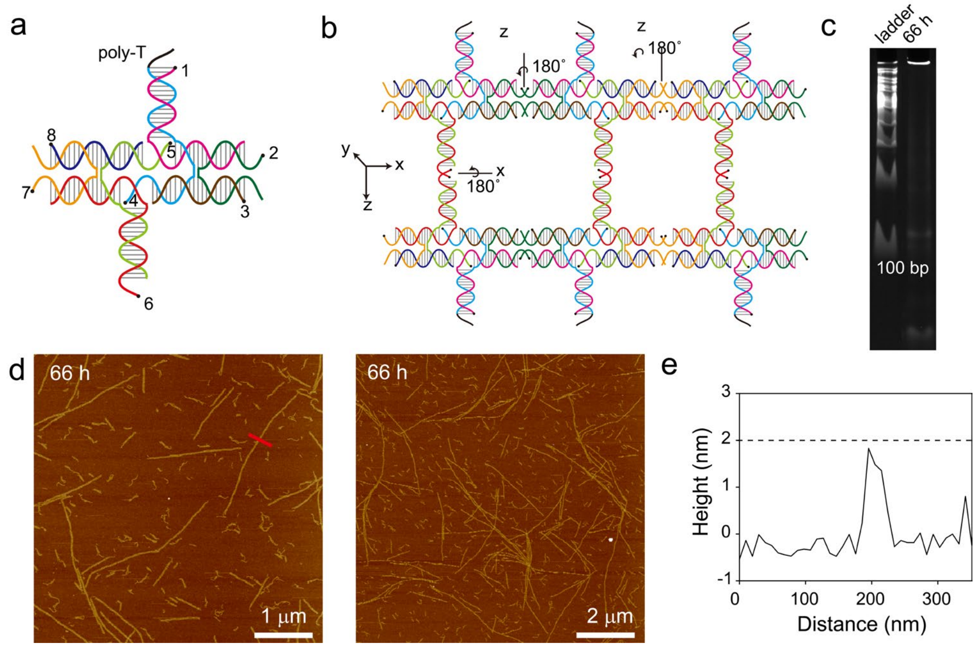

Supplementary Figure 16 | Schematics and characterization of the design of TC-8-2. a) The structural design of TC-8-2. b) Sticky ends matching rules. c) Native gel images of TC-8-2. d) AFM images (66 h annealing program). e) Height measurement of TC-8-2. The red line labeled in AFM images shows the height data extract location. Source data are provided as a Source Data file.

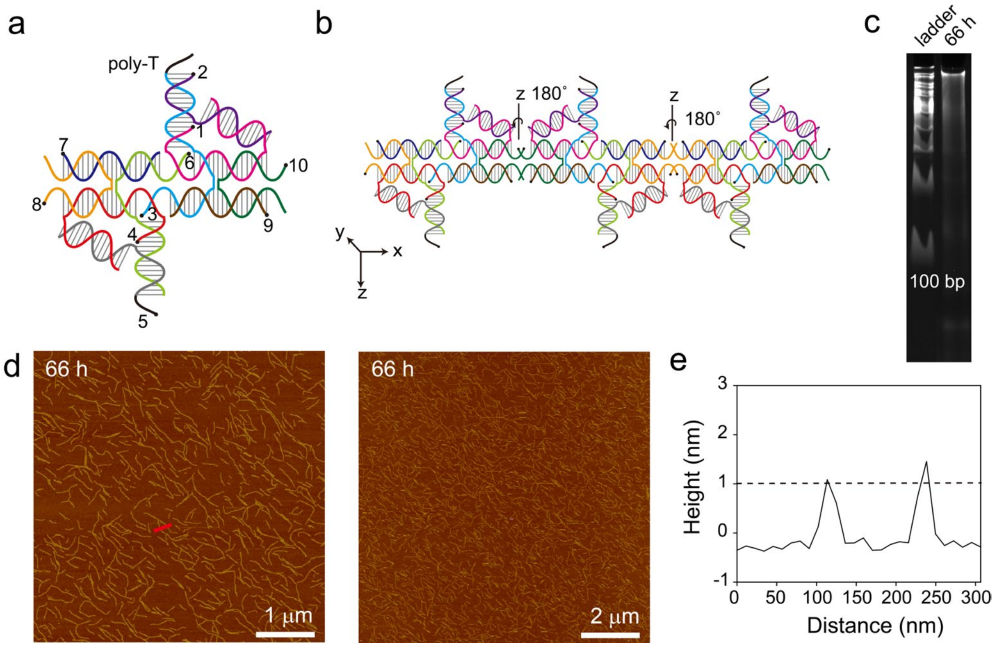

Supplementary Figure 17 | Schematics and characterization of the design of TC-9-1. a) The structural design of TC-9-1. b) Sticky ends matching rules. c) Native gel images of TC-9-1. d) AFM images (66 h annealing program). e) Height measurement of TC-9-1. The red line labeled in AFM images shows the height data extract location. Source data are provided as a Source Data file.

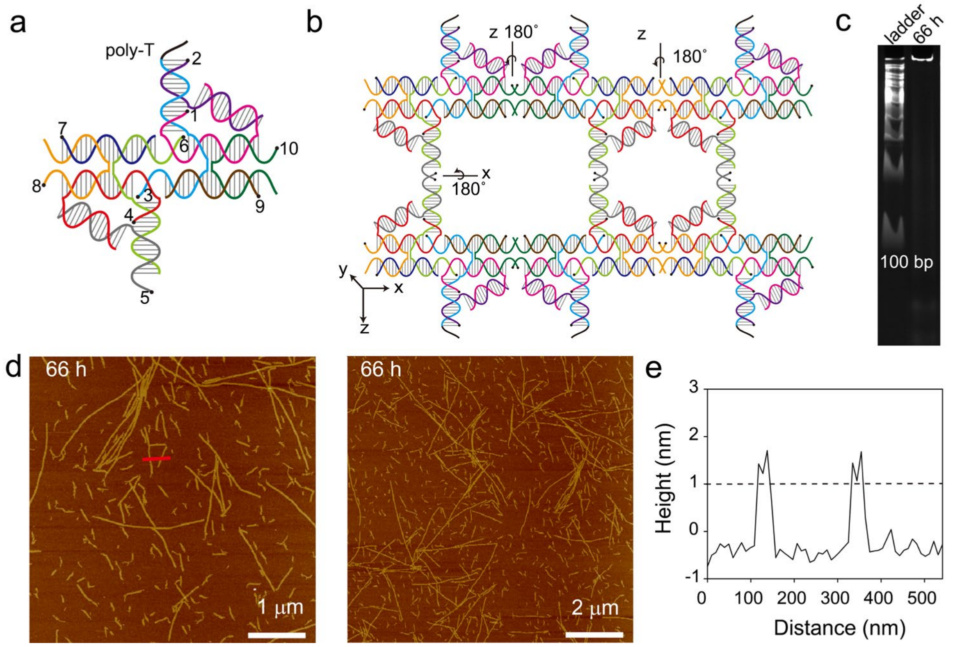

Supplementary Figure 18 | Schematics and characterization of the design of TC-9-2. a) The structural design of TC-9-2. b) Sticky ends matching rules. c) Native gel images of TC-9-2. d) AFM images (66 h annealing program). e) Height measurement of TC-9-2. The red line labeled in AFM images shows the height data extract location. Source data are provided as a Source Data file.

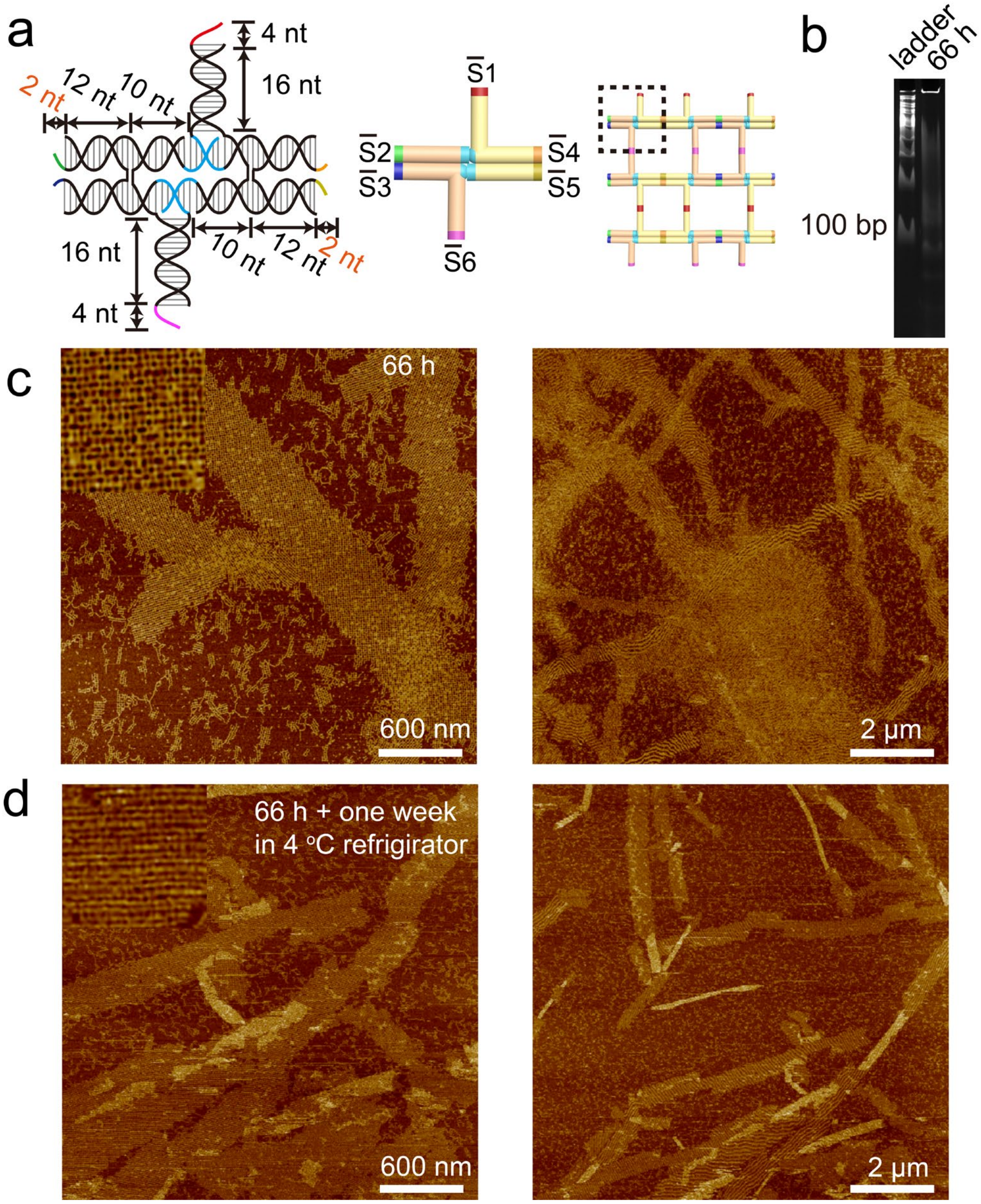

Supplementary Figure 19 | Schematics and characterization of the design of TC-8-4. a) The structural design and b) native gel images of TC-8-4. AFM images of TC-8-4, c) 66 h annealing program, d) 66 h annealing program and one week storage in 4 °C refrigerator. Source data are provided as a Source Data file.

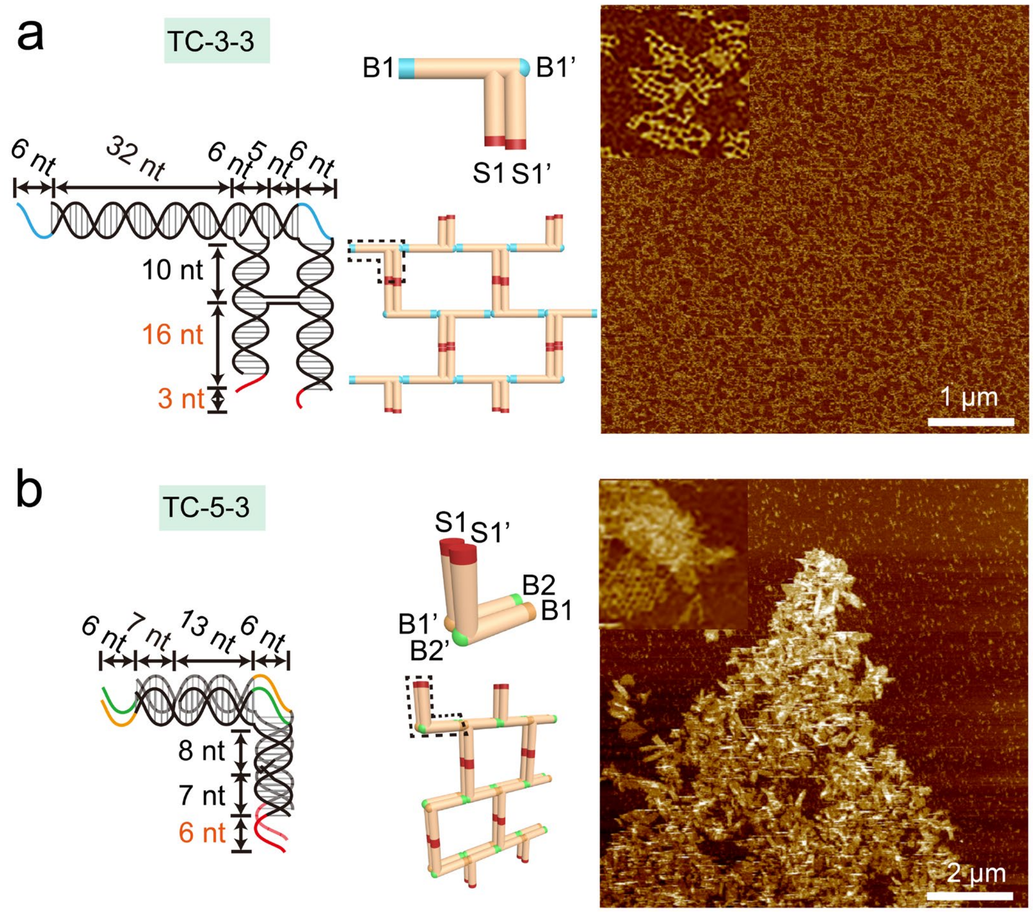

Supplementary Figure 20 | Schematics and characterization of the design of a) TC-3-3 and b) TC-5-3.

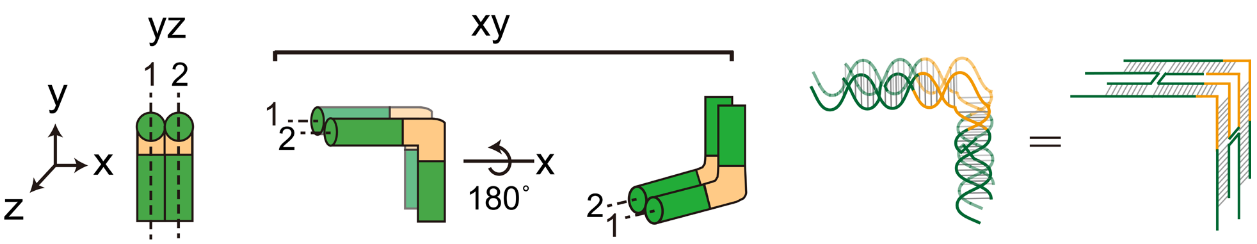

Supplementary Figure 21 | Structural for the design of two-layer TC tiles. The layers are labeled as numbers 1 and 2, with each dashed line denoting the center of an individual layer.

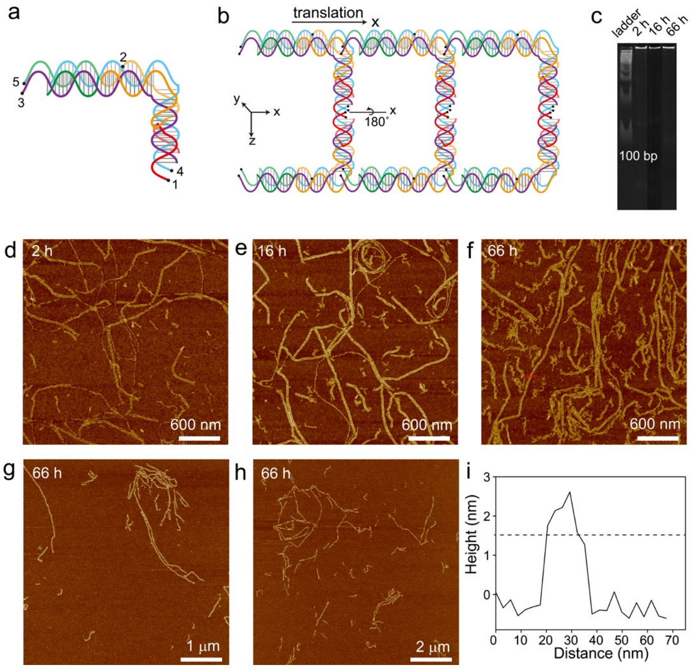

Supplementary Figure 22 | Schematics and characterization of the design of TC-5-1. a) The structural design of TC-5-1. b) Sticky ends matching rules. c) Native gel images of TC-5-1. d-h) AFM images (2h, 16 h, 66 h annealing program). i) Height measurement of TC-5-1. The red line labeled in AFM images shows the height data extract location. Source data are provided as a Source Data file.

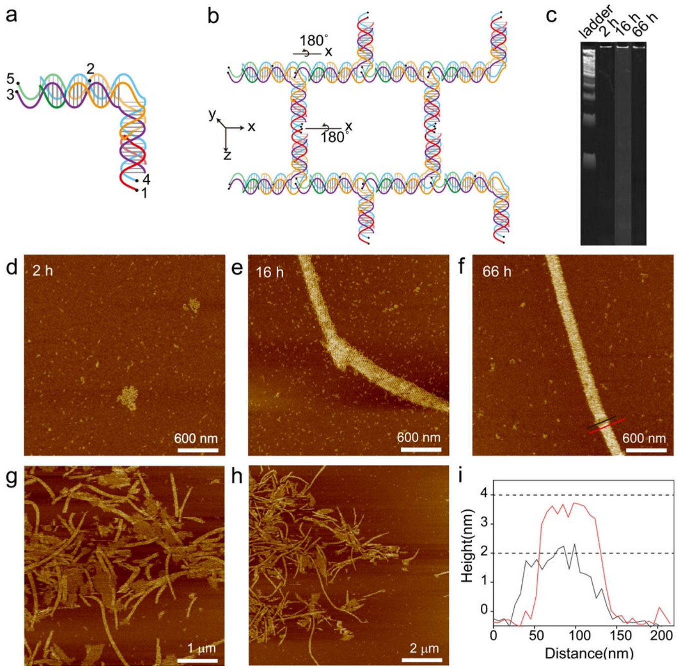

Supplementary Figure 23 | Schematics and characterization of the design of TC-5-2. a) The structural design of TC-5-2. b) Sticky ends matching rules. c) Native gel images of TC-5-2. d-h) AFM images (2h, 16 h, 66 h annealing program). i) Height measurement of TC-5-2. The red and black line labeled in AFM images show the height data extract location. Source data are provided as a Source Data file.

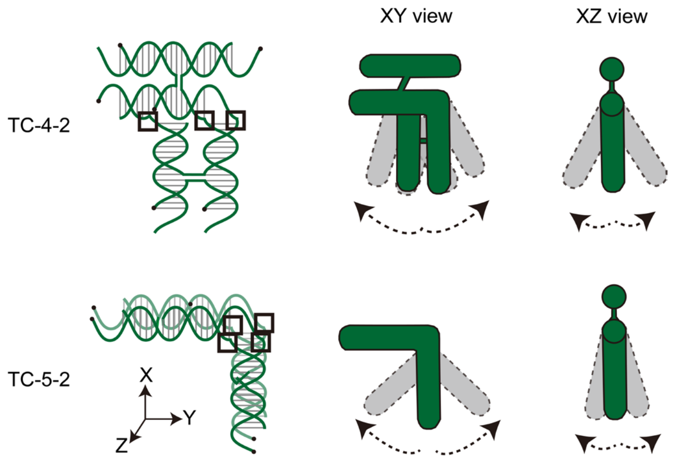

Supplementary Figure 24 | Schematic showing the flexibility of TC-4-2 and TC-5-2. The joint spots between horizontal and vertical helices are labeled by black square.

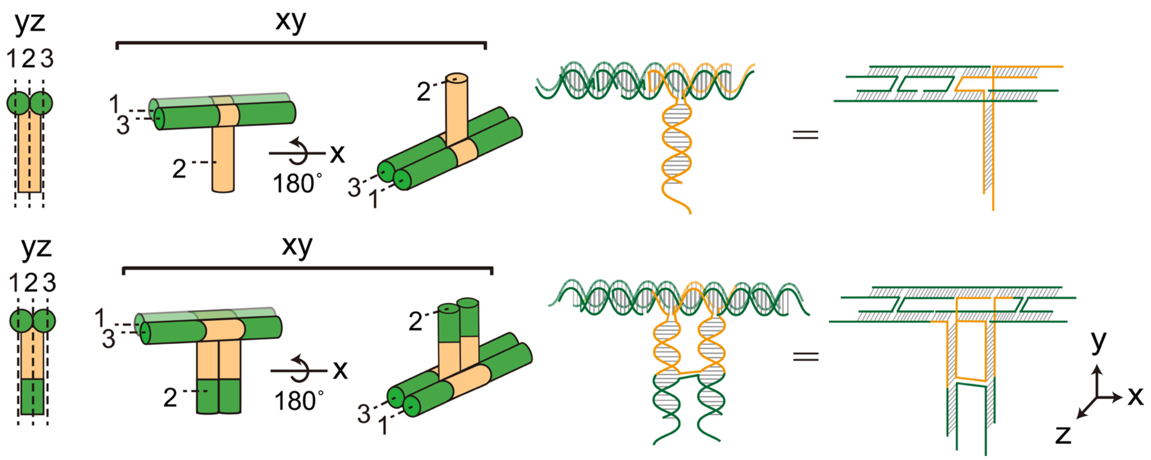

Supplementary Figure 25 | Structural design of three-layered DX-T tiles. The layers are labeled as numbers 1, 2, and 3, with each dashed line denoting the center of an individual layer.

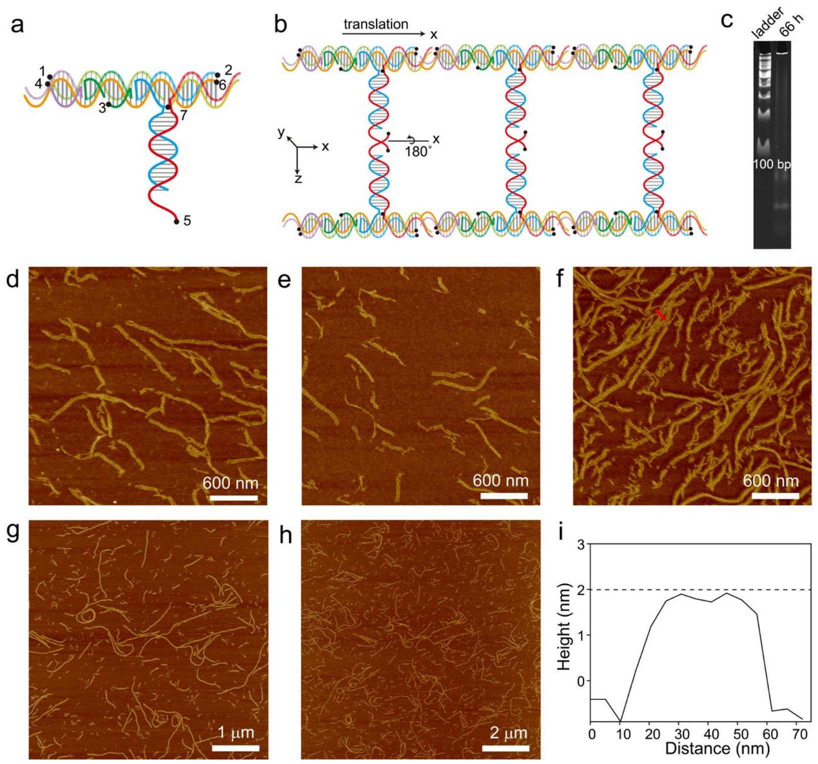

Supplementary Figure 26 | Schematics and characterization of the design of TC-6-1. a) The structural design of TC-6-1. b) Sticky ends matching rules. c) Native gel images of TC-6-1. d-h) AFM images (2h, 16 h, 66 h annealing program). i) Height measurement of TC-6-1. The red line labeled in AFM images shows the height data extract location. Source data are provided as a Source Data file.

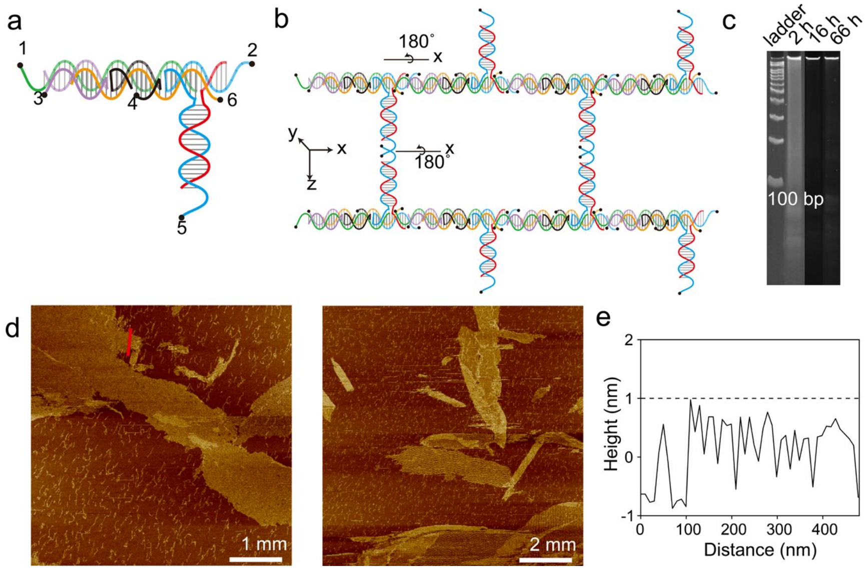

Supplementary Figure 27 | Schematics and characterization of the design of TC-6-2. a) The structural design of TC-6-2. b) Sticky ends matching rules. c) Native gel images of TC-6-2. d) AFM images (66 h annealing program). e) Height measurement of TC-6-2. The red line labeled in AFM images shows the height data extract location. Source data are provided as a Source Data file.

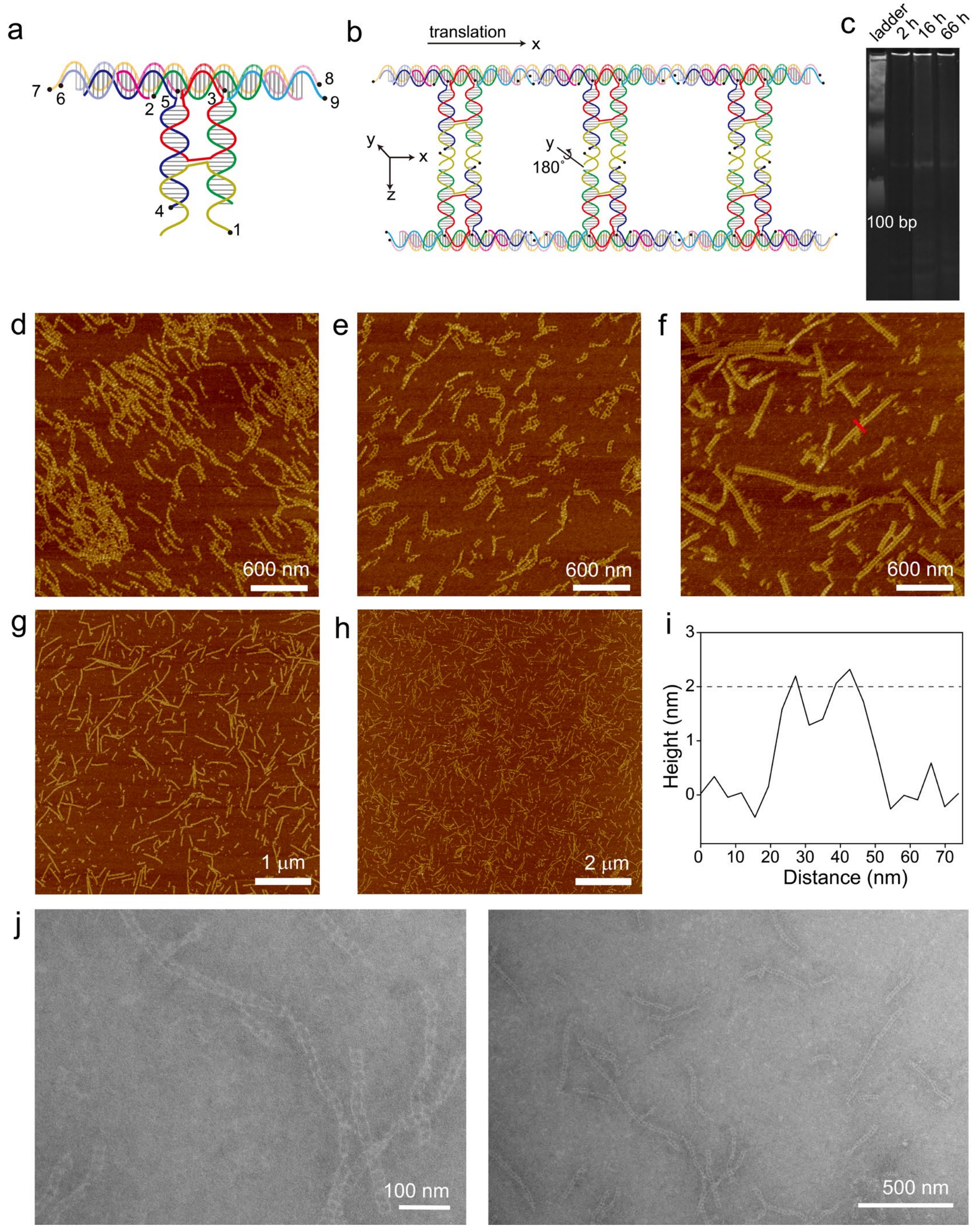

Supplementary Figure 28 | Schematics and characterization of the design of TC-7-1 a) The structural design of TC-7-1. b) Sticky ends matching rules. c) Native gel images of TC-7-1. d-h) AFM images (2h, 16 h, 66 h annealing program). i) Height measurement of TC-7-1. j) The TEM images of TC-7-1 (66 h annealing program). The red line labeled in AFM images shows the height data extract location. Source data are provided as a Source Data file.

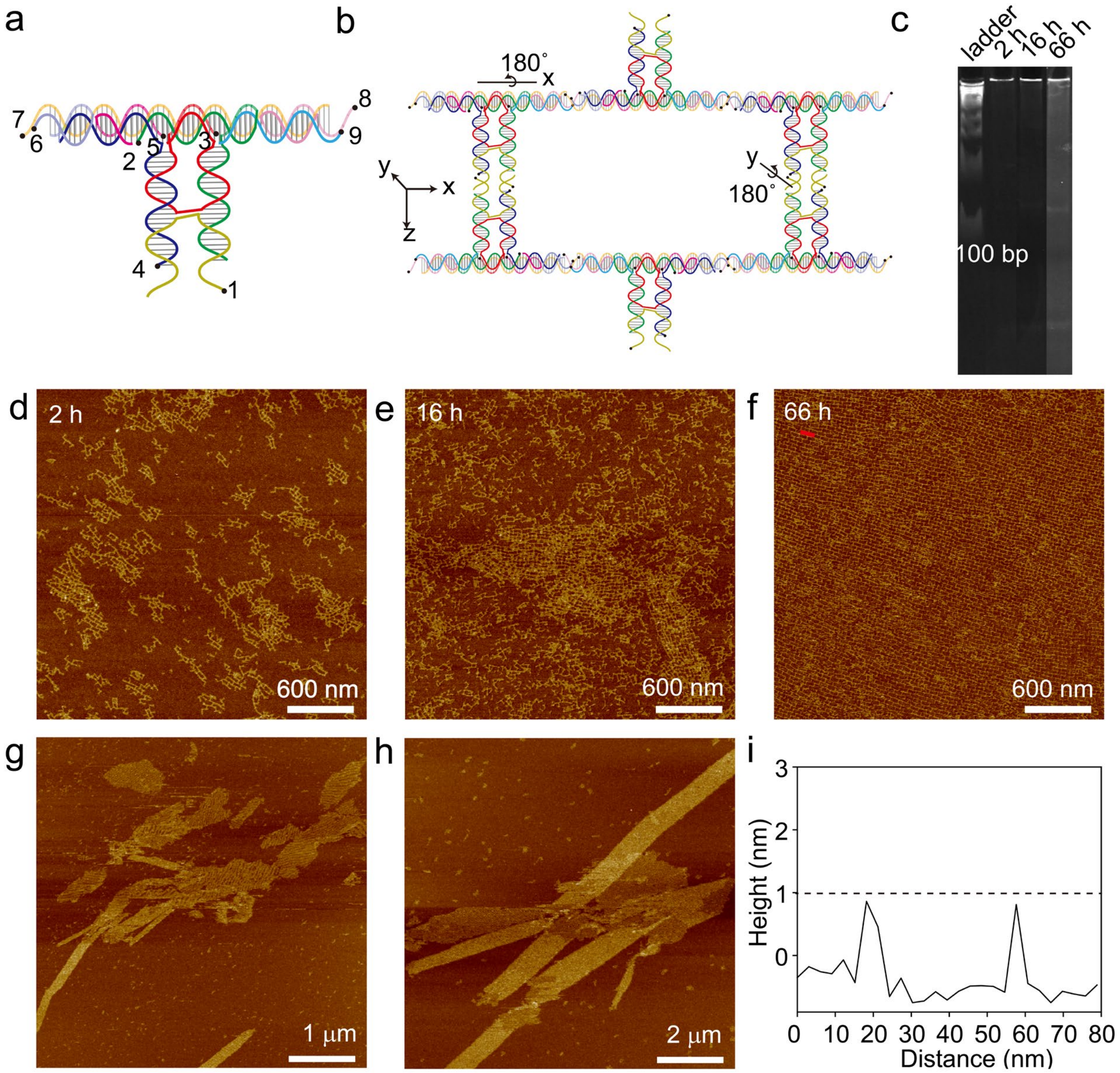

Supplementary Figure 29 | Schematics and characterization of the design of TC-7-2. a) The structural design of TC-7-2. b) Sticky ends matching rules. c) Native gel images of TC-7-2. d-h) AFM images (2h, 16 h, 66 h annealing program). i) Height measurement of TC-7-2. The red and black line labeled in AFM images show the height data extract location. Source data are provided as a Source Data file.

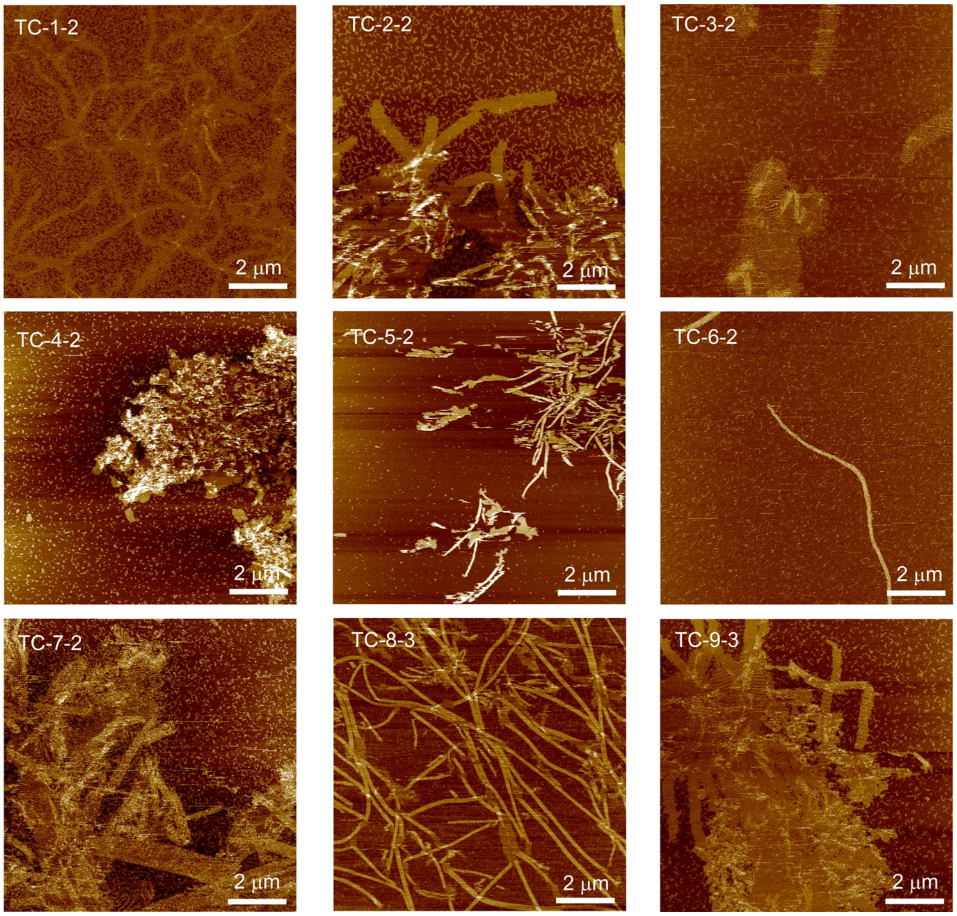

Supplementary Figure 30 | AFM images of all the tested TC tiles that target to form grids that assembled under 66 h annealing program and followed by one week storage in 4 °C refrigerator.

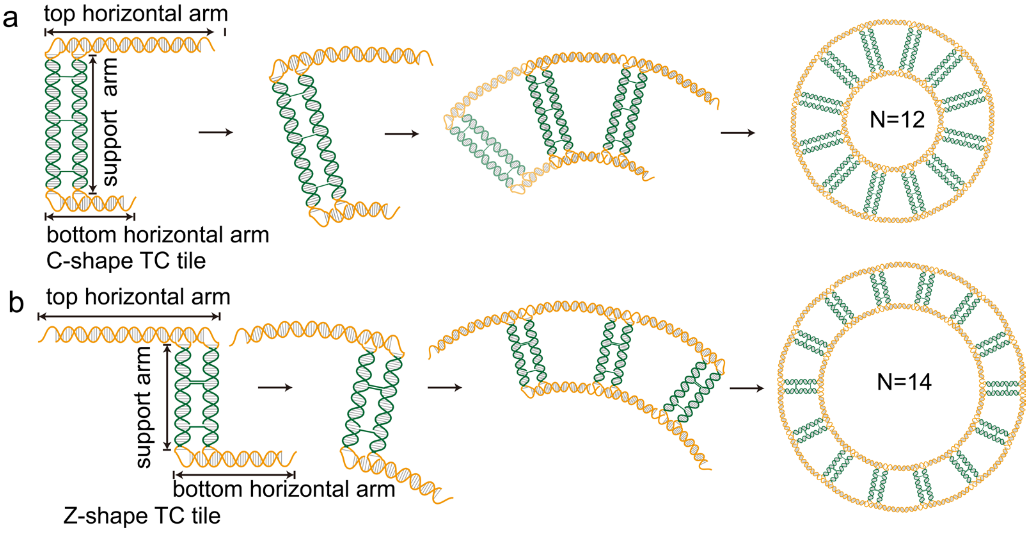

Supplementary Figure 31 | Schematics of C-shape and Z-shape TC tile that target defined ring shape. The two tiles are named TC-C-6-5.5-3 and TC-Z-6-4-4, respectively. The numbers represent the length of horizontal arms and support arm. For example, TC-C-6-5.5-3 means a C shape TC tile with a top horizontal arm of 6 helical turns of DNA, a supporting arm of 5.5 helical turns and a bottom horizontal arm of 3 helical turns.

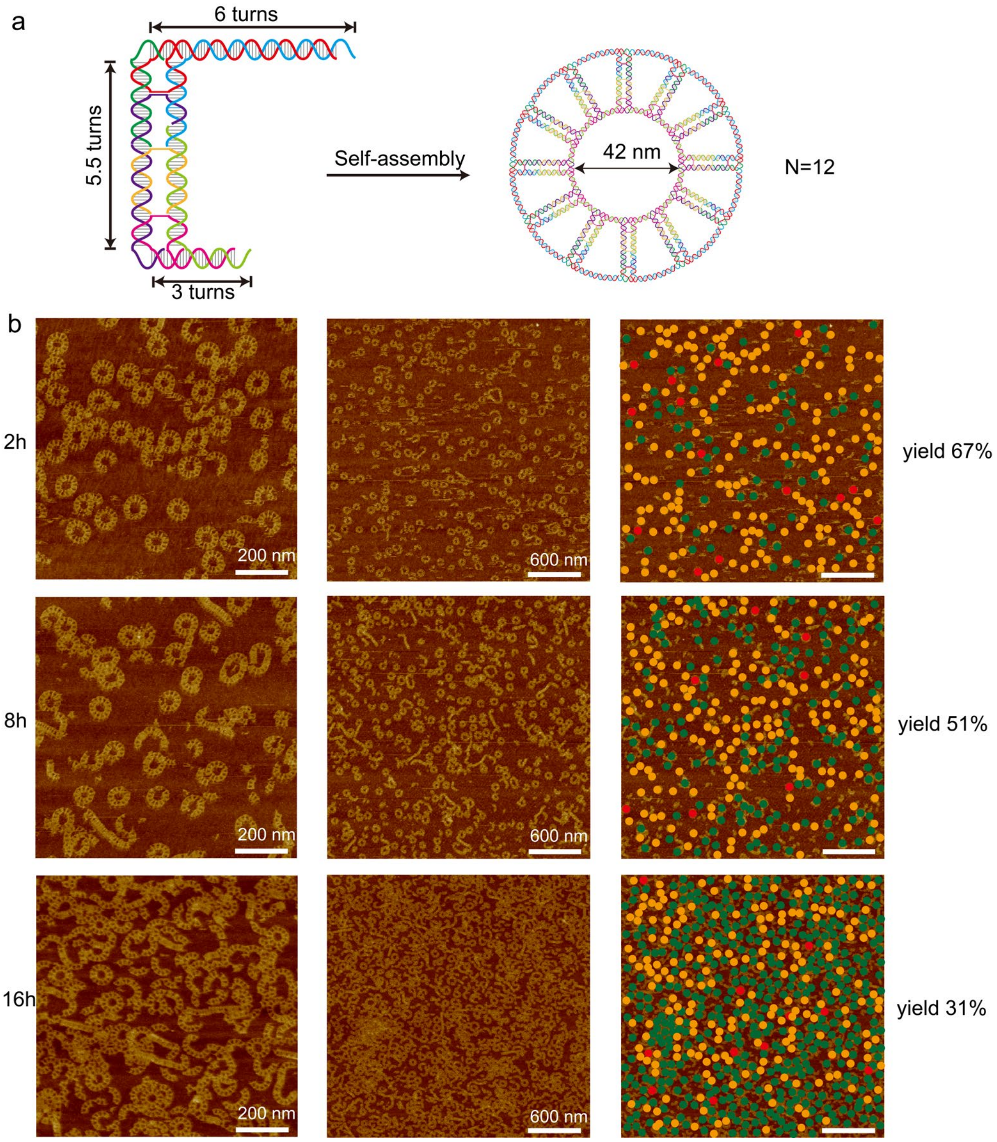

Supplementary Figure 32 | Schematics and AFM images of the defined ring shape formed by TC-C-6-5.5-3. a) Schematics of TC-C-6-5.5-3 and nanoring. b) The AFM images of defined ring under three different annealing programs (2 h, 8 h and 16h). The estimated assembly yield of target nanoring is calculated based on the AFM images. The yellow circle labels the well-formed ring (N=12), red circle labels well-formed ring (N=13), and the green circle labeled the by-products. Notes, one green circle labeled nanostructure is assumed to have the same number of monomers as a well-formed nanoring (N=12). The estimated yield of nanorings for each sample, 2 h, 268 nanorings (yellow 181, red 14, green 73), yield = 181×12 / (181×12+14×13+73×12) ≈ 67% (N=12); 8 h, 325 nanorings (yellow 167, red 7, green 151), yield = 167×12 / (167×12+7×13+151×12) ≈ 51% (N=12); 16 h, 643 nanorings (yellow 200, red 12, green 431), yield = 200×12 / (200×12+12×13+431×12) ≈ 31% (N=12). Source data are provided as a Source Data file.

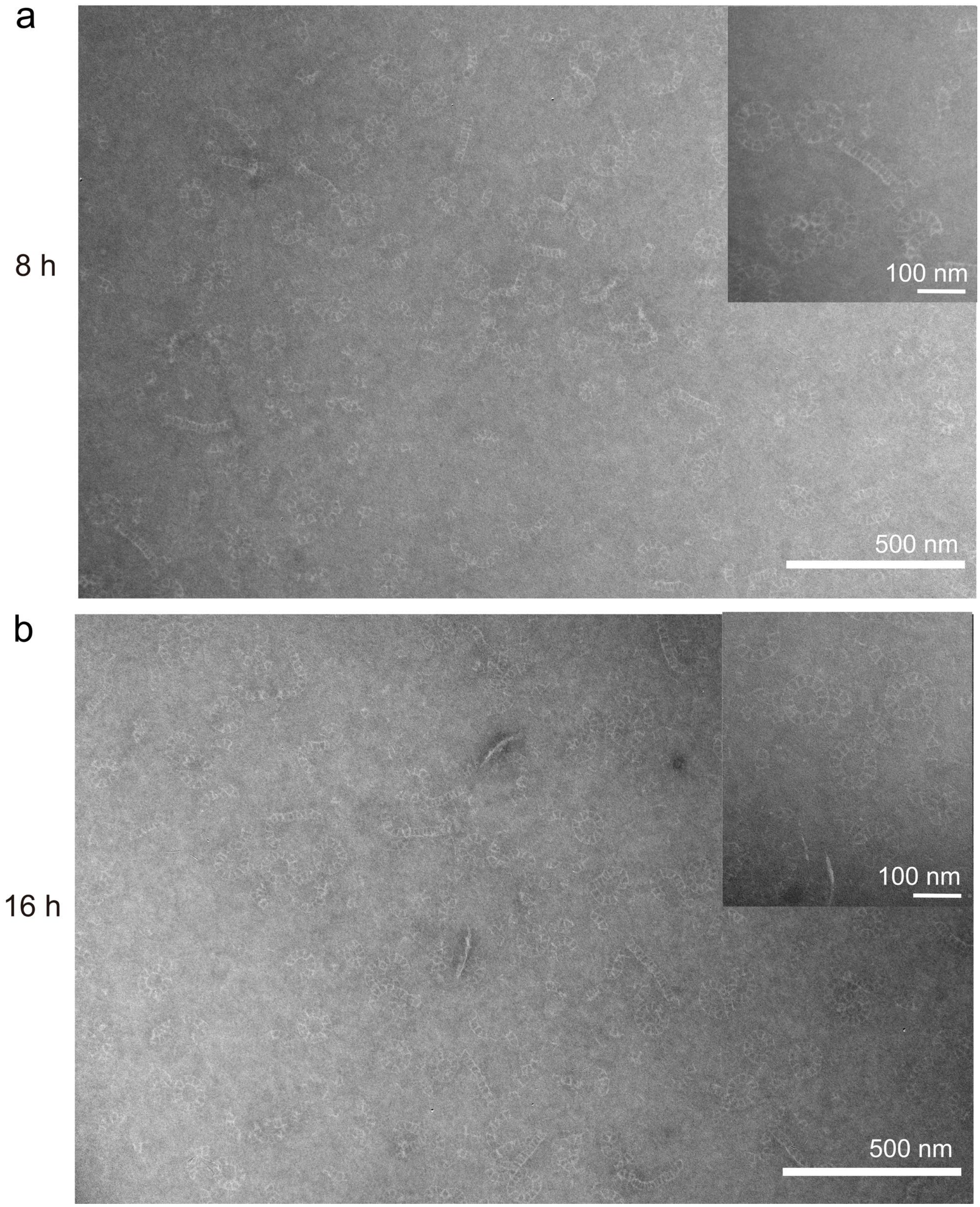

Supplementary Figure 33 | TEM mages of TC-C-6-5.5-3 assembled nanorings, a) 8 h and b) 16 h annealing program.

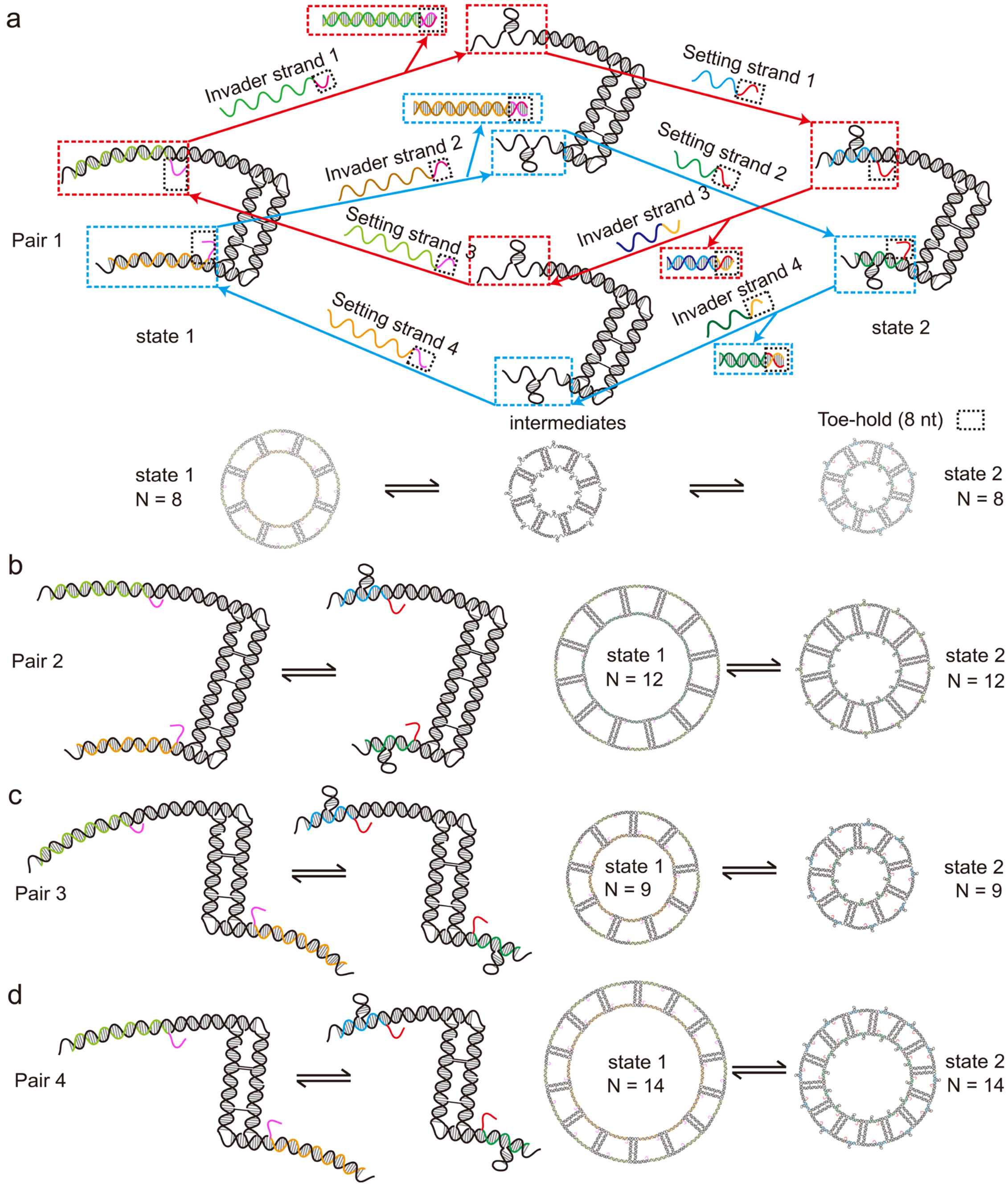

Supplementary Figure 34 |. The schematics of the transformation between two-state tiles and nanorings. a) pair 1, *N*=8, TC-C-9-3.5-6 $\rightleftharpoons$ TC-C-7-3.5-4. b) pair 2, *N*=12, TC-C-9-5.5-6 $\rightleftharpoons$ TC-C-7-5.5-4, c) pair 3, *N*=9, TC-Z-9-4-6 $\rightleftharpoons$ TC-C-7-4-4 and d) pair 4, *N*=14, TC-Z-8-4-6 $\rightleftharpoons$ TC-Z-6-4-4. Notes, four pairs of two states nanorings dynamics share same invaders strands and setting strands, the schematics are the same. For example, pair 1 of TC-C-9-3.5-6 transform to TC-C-7-3.5-4 by two steps. After adding the invader strands 1 and 2, the invader strands start to bind with the toehold, then displace the two complementary strands from the state 1 structure, so that an intermediate with a partially single stranded horizontal arm is generated. After adding two setting strands 1 and 2, they regionally bind with the single stranded horizontal arms and output the state 2 structure. Reversibly, by the two step addition of invader strands (3, 4) and setting strands (3, 4), state 2 will switch back to state 1.

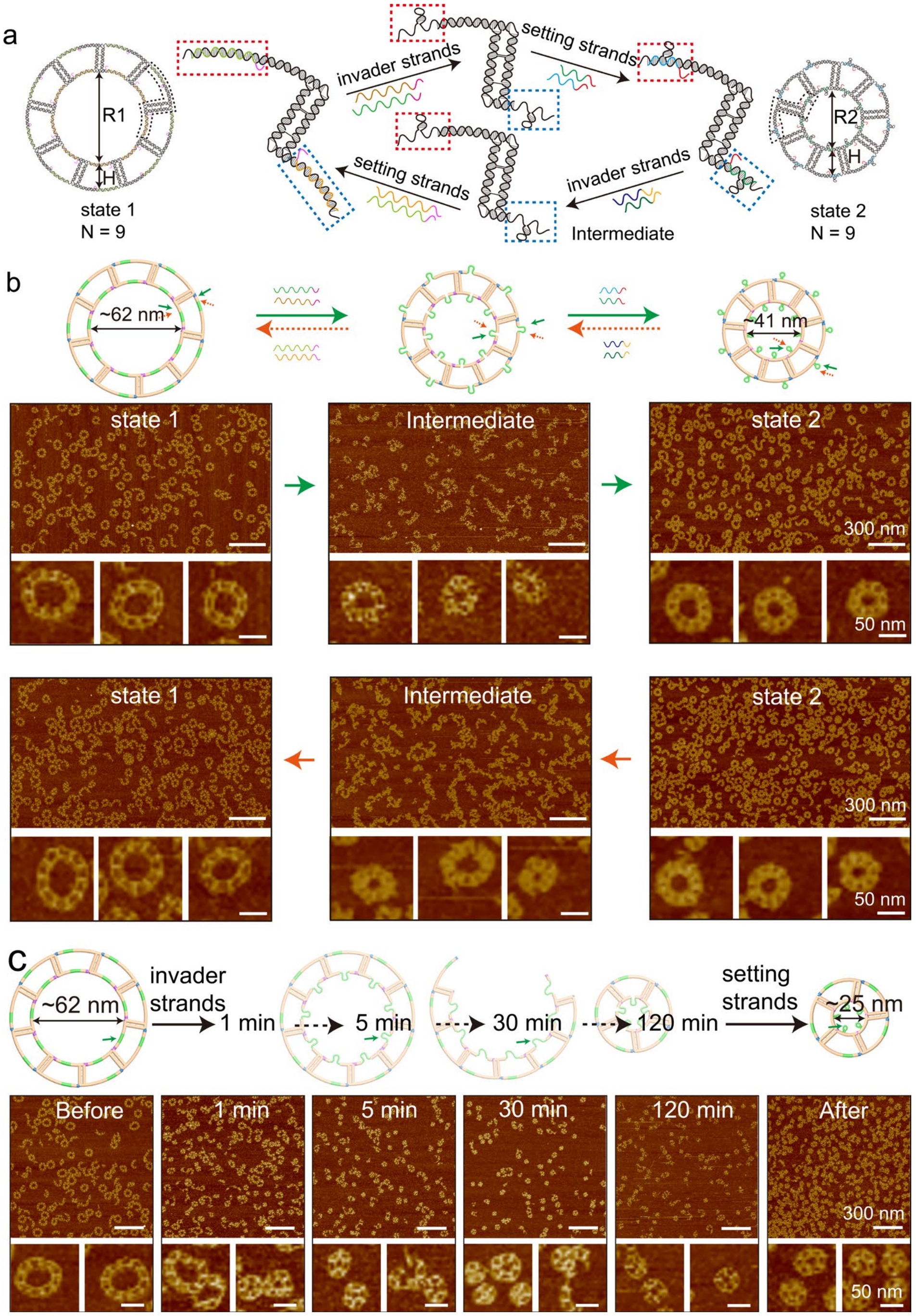

Supplementary Figure 35 | The study of intermediates between two states nanoring based on Z-Shape TC tiles. a) Schematics of nanoring transformation induced by SDR. From state 1 to state 2, two invader strands and two setting strands were added, and vice versa. b) The schematics and AFM images of two nanoring transformations, transformed intermediates and products. The *N* retains the same as state 1 after transformation. The typical assemblies are cropped out and presented. c) The schematics and AFM images of a big nanoring transform to small nanoring with *N* value decrease (From *N* = 8 to *N* = 5). After adding invader strands four of the time points are tracked by AFM imaging, including 1min, 5 min, 30 min and 120 min. Source data are provided as a Source Data file.

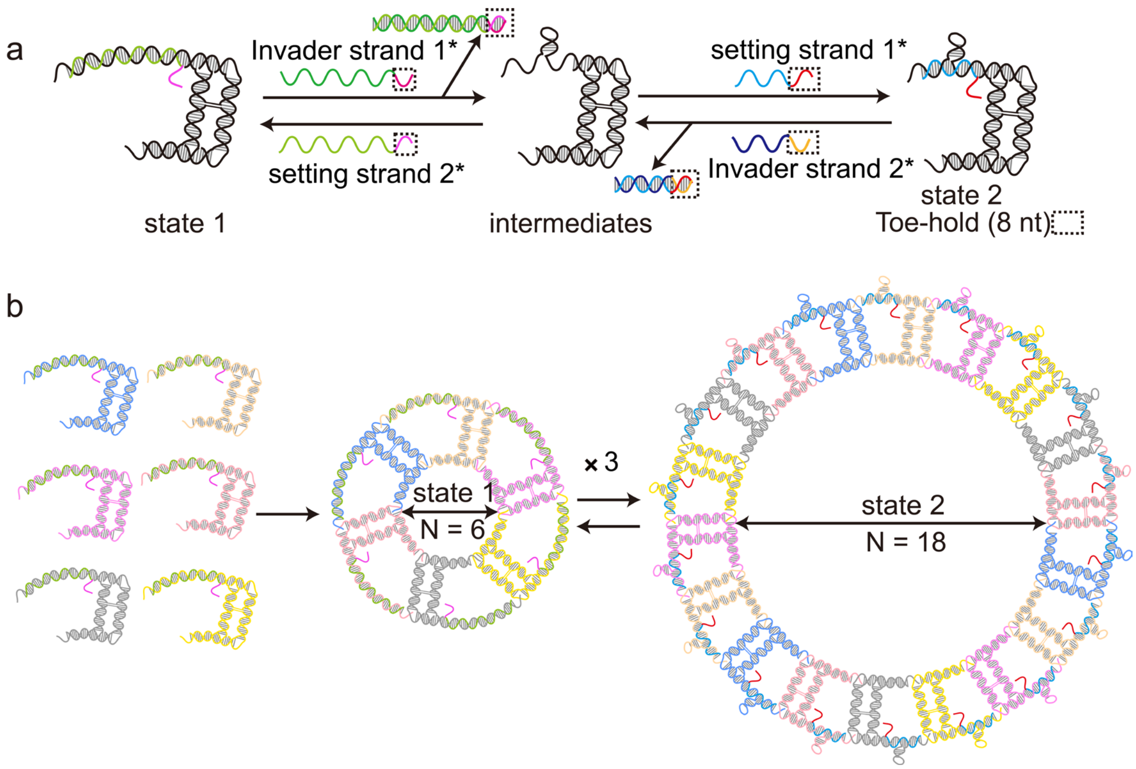

Supplementary Figure 36 | Schematics of the reconfigurable SST nanorings. a) The C-shape TC tiles that were used for SST nanoring. The tile for state 1 is TC-C-6-2.5-3 and state 2 is TC-C-4-2.5-3. b) Two-state SST nanorings. The 6-monomer nanoring consisted of six TC-C-6-2.5-3 monomers with different color-coded sequences, with well-defined matching rules between sticky ends. The information of invader and setting strands are summarized in Supplementary table 14.

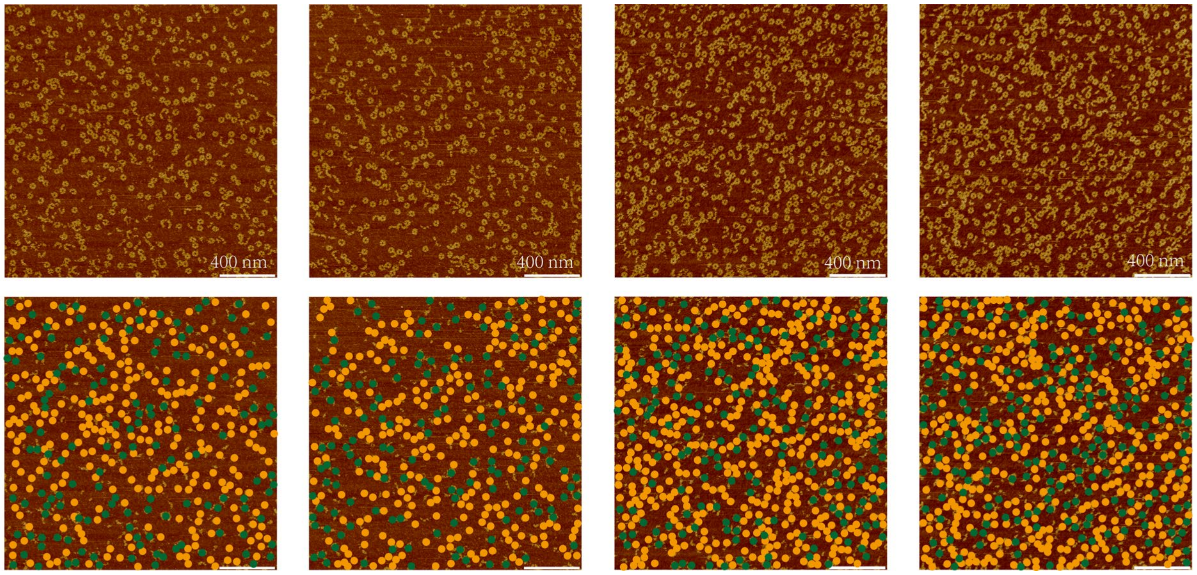

Supplementary Figure 37 | The estimated assembly yield of small SST nanorings (without SDR toeholds, N = 6). Four of 2 μm ×2 μm AFM images are used for yield counting, with 2093 total counted nanorings (yellow 1401, green 692). The calculated yield is 67%. Source data are provided as a Source Data file.

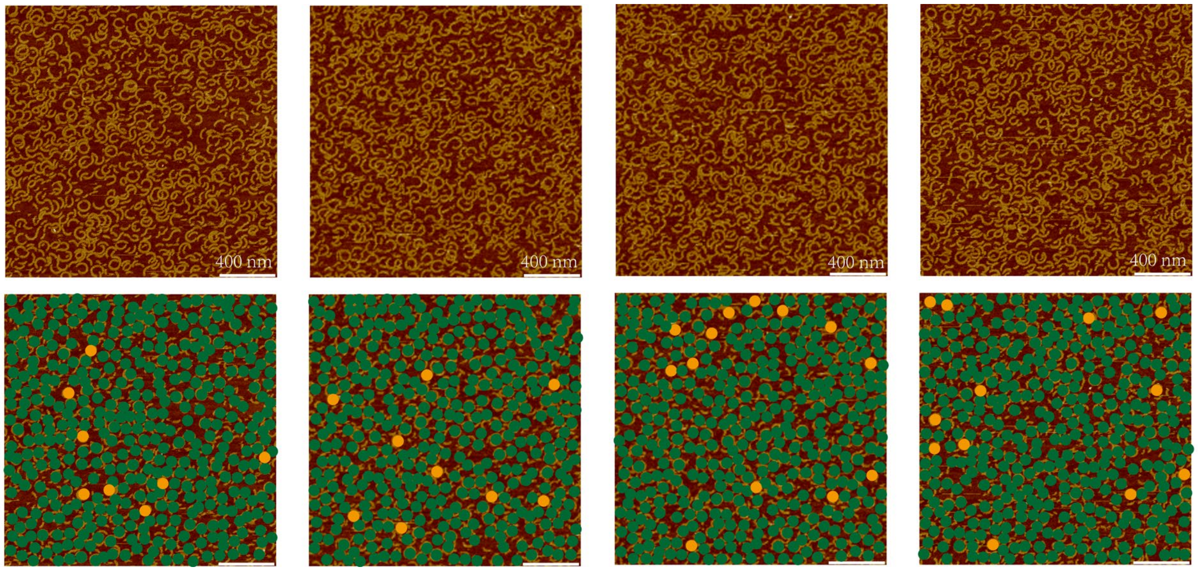

Supplementary Figure 38 | The estimated assembly yield of big SST nanorings (without SDR toeholds, N = 18). Four of 2 μm ×2 μm AFM images are used for yield counting, with 1613 total counted nanorings (yellow 42, green 1571). The calculated yield is 3%. Source data are provided as a Source Data file.

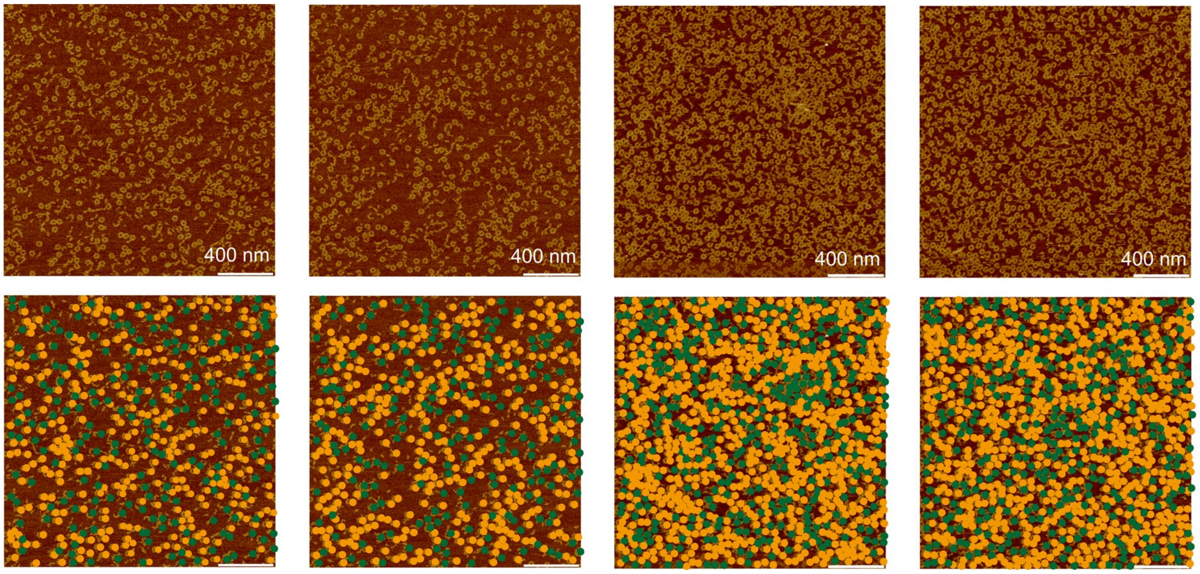

Supplementary Figure 39 | The estimated assembly yield of small SST nanorings (with SDR toeholds, N = 6). Four of 2 μm ×2 μm AFM images are used for yield counting, with 3166 total counted nanorings (yellow 2061, green 1105). The calculated yield is 65%. Source data are provided as a Source Data file.

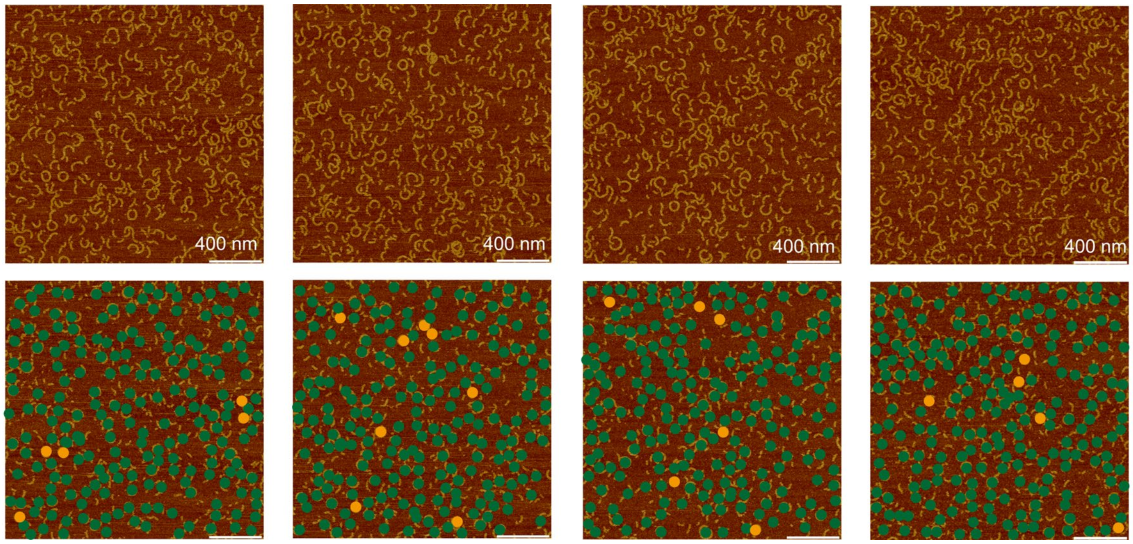

Supplementary Figure 40 | The estimated assembly yield of big SST nanorings (with SDR toeholds, N = 18). Four of 2 μm ×2 μm AFM images are used for yield counting, with 862 total counted nanorings (yellow 24, green 838). The calculated yield is 3%. Source data are provided as a Source Data file.

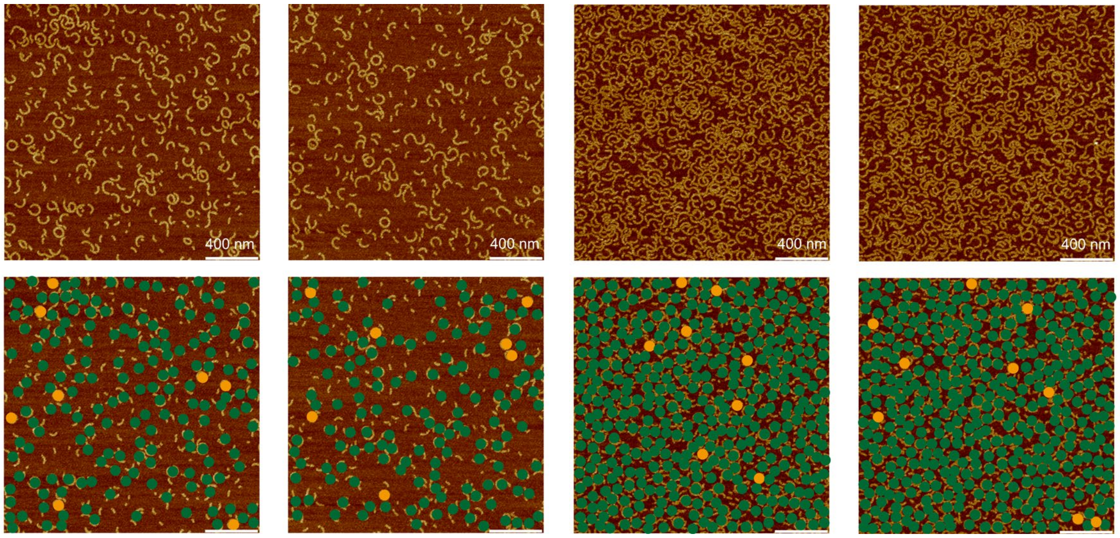

Supplementary Figure 41 | The estimated assembly yield of transformed big SST nanorings (N = 18). Four of 2 μm ×2 μm AFM images are used for yield counting, with 1146 total counted nanorings (yellow 32, green 1114). The calculated yield is 3%. Source data are provided as a Source Data file.

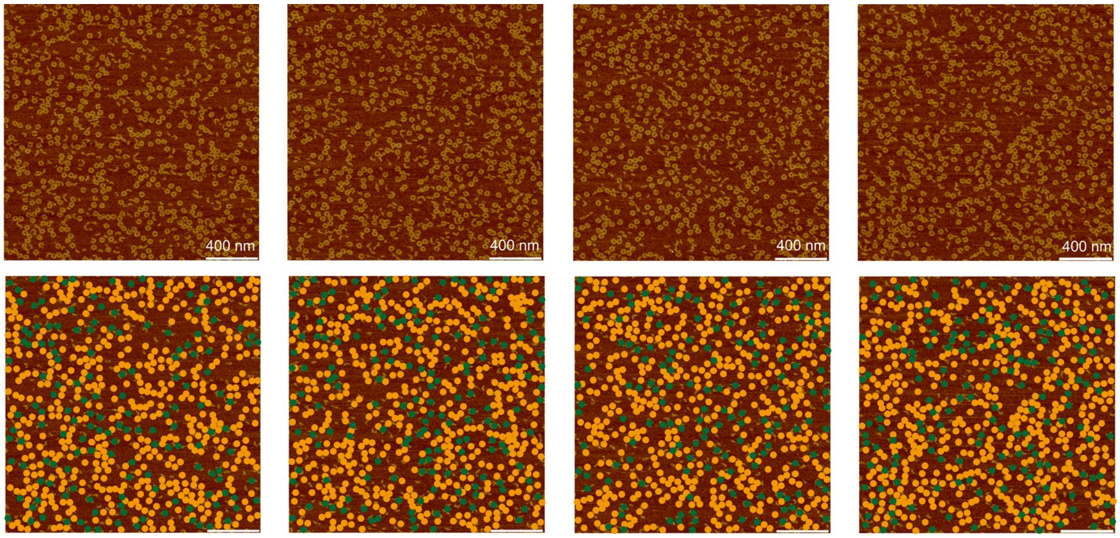

Supplementary Figure 42 | The estimated assembly yield of transformed small SST nanorings (N = 6). Four of 2 μm ×2 μm AFM images are used for yield counting, with 2592 total counted nanorings (yellow 1958, green 634). The calculated yield is 76%. Source data are provided as a Source Data file.

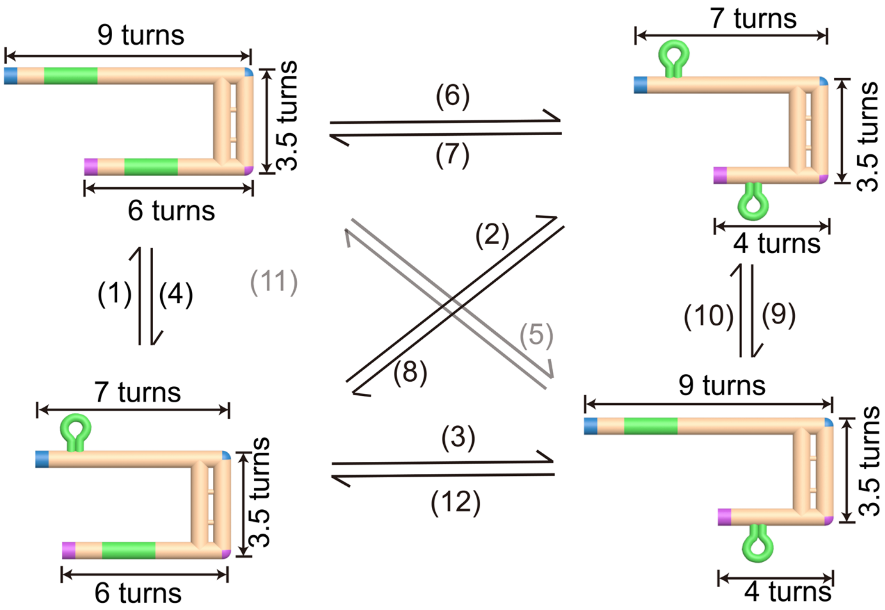

Supplementary Figure 43 | The schematics of the transformation between four-state tiles of pair 1 C-shape TC tiles. The four tiles are TC-C-9-3.5-6, TC-C-7-3.5-6, TC-C-9-3.5-4, and TC-C-7-3.5-4.

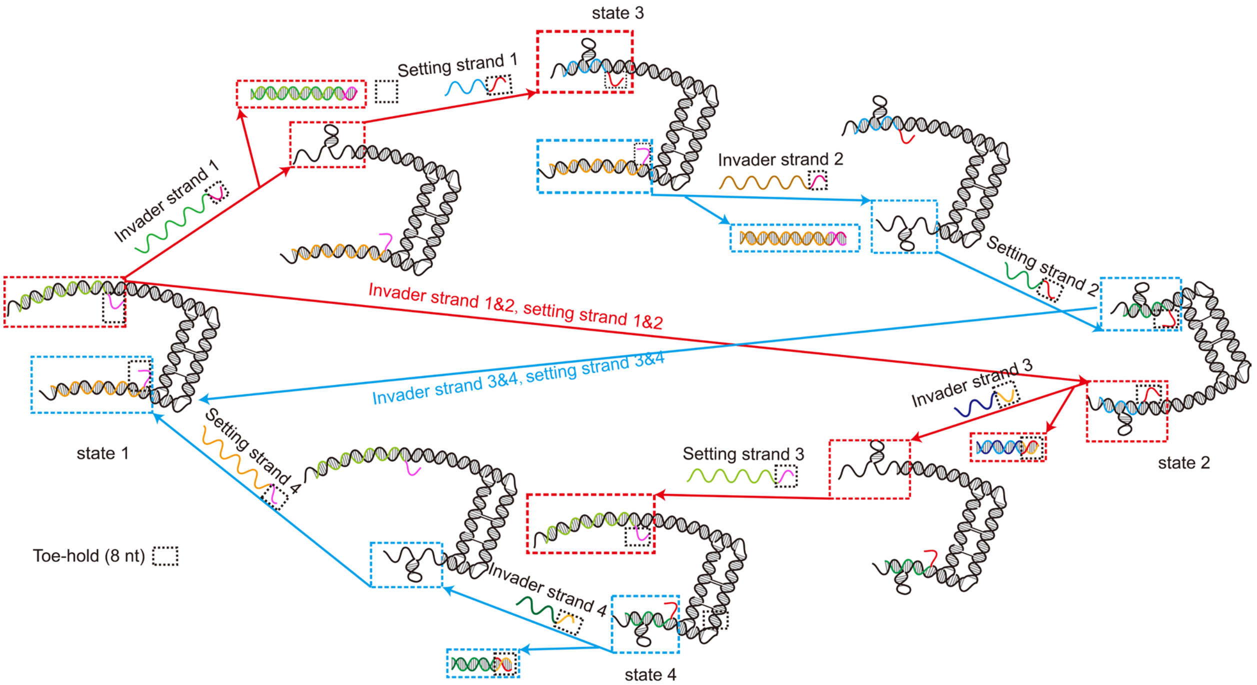

Supplementary Figure 44 |. The schematics of the transformation between four-state TC tiles and nanorings. Notes, four pairs of four states nanorings dynamics shared same invaders strands and setting strands, the schematics are same. Here, we use pair 1 C-shape TC tiles as an example. From state 1 to state 3, by adding invader strand 1 and setting strand 1, TC-C-9-3.5-6 transform to TC-C-7-3.5-6. From state 1 to state 2, by adding invader strand 1&2 and setting strand 1&2, TC-C-9-3.5-6 transform to TC-C-7-3.5-4. From state 3 to state 2, by adding invader strand 2 and setting strand 2, TC-C-9-3.5-6 transform to TC-C-7-3.5-6. From state 2 to state 1, by adding invader strand 3&4 and setting strand 3&4, TC-C-9-3.5-6 transform to TC-C-7-3.5-4.

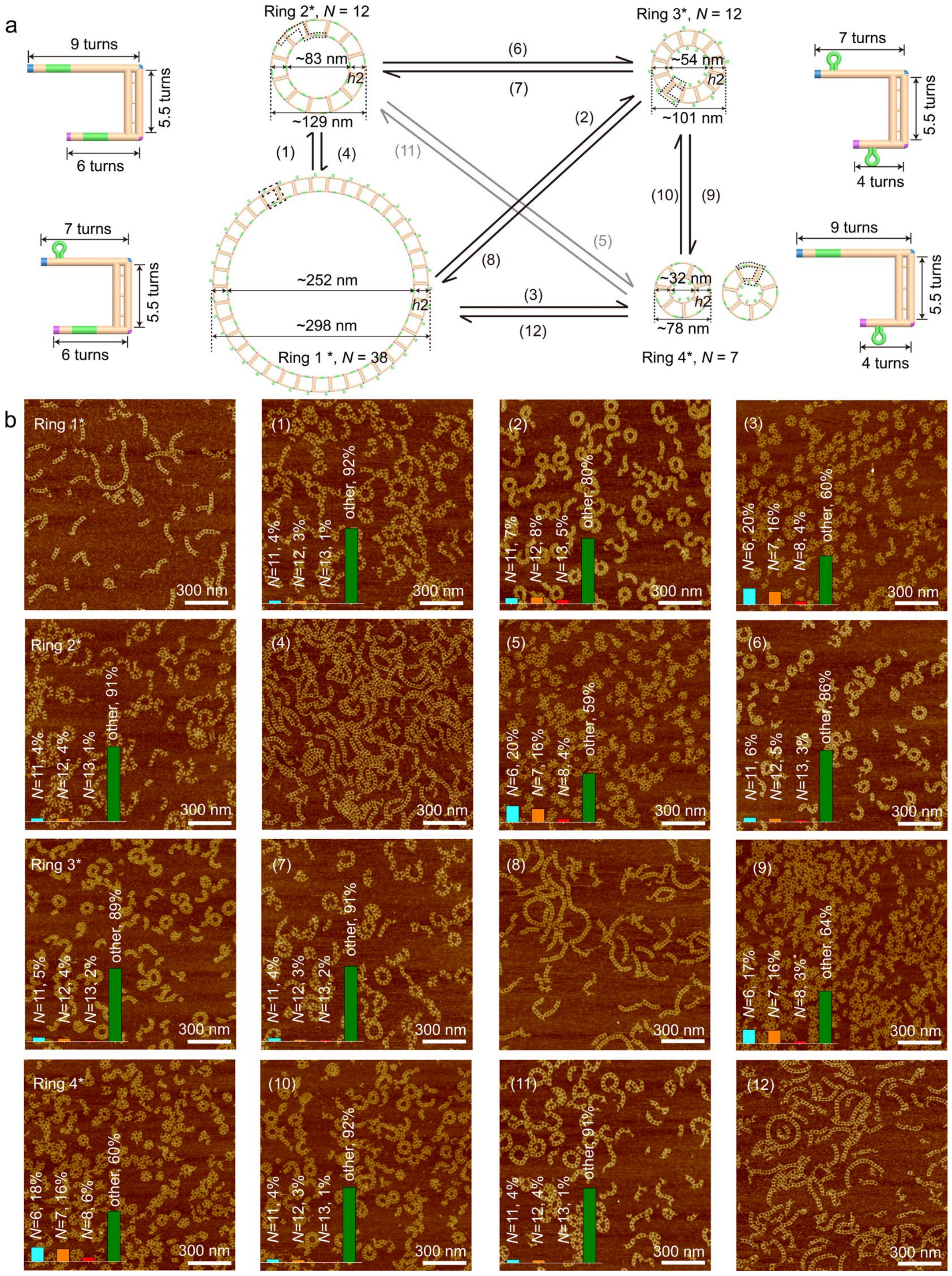

Supplementary Figure 45 | 4-states ring-to-ring reconfiguration based on pair 2 C-shape TC tiles. a) Schematics of the one-to-one reconfiguration process between four rings, through a total of 12 paths. Each path is labeled with a number from (1) to (12), and the arrow shows the reconfiguration direction. b) The AFM images of each nanoring and the reconfiguration product with yield. The first row presents the Ring 1* and its reconfiguration products through path 1, path 2 and path 3, from left to right, respectively. Similarly, the last three rows depict Ring 2*, Ring 3* and Ring 4* and corresponding three paths reconfigured nanorings, from top to bottom, respectively. Source data are provided as a Source Data file.

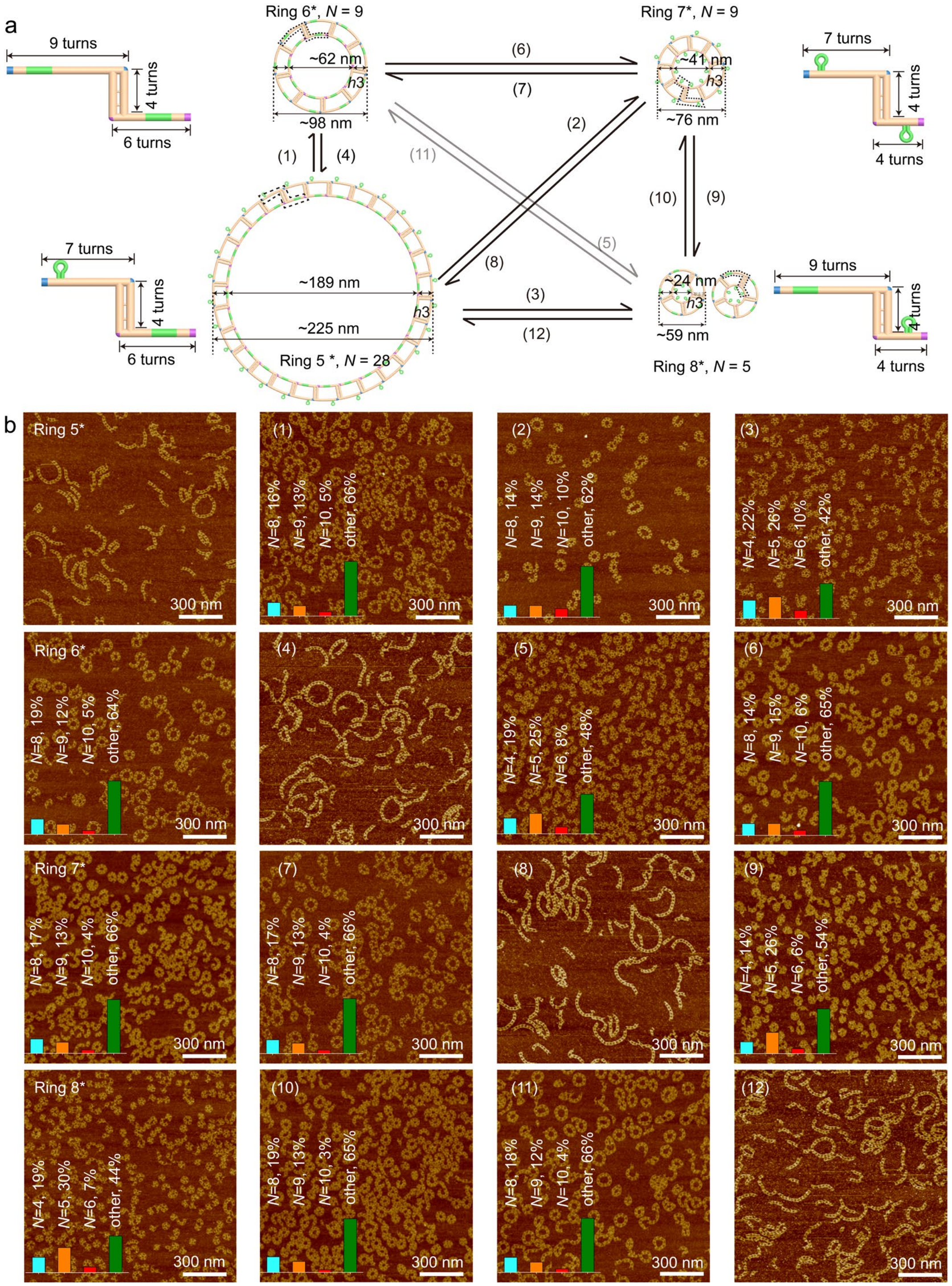

Supplementary Figure 46 | 4-states ring-to-ring reconfiguration based on pair 3 Z-shape TC tiles. a) Schematics of the one-to-one reconfiguration process between four rings, through a total of 12 paths. Each path is labeled with a number from (1) to (12), and the arrow shows the reconfiguration direction. b) The AFM images of each nanoring and the reconfiguration product with yield. The first row presents the Ring 5* and its reconfiguration products through path 1, path 2 and path 3, from left to right, respectively. Similarly, the last three rows depict Ring 6*, Ring 7* and Ring 8* and corresponding three paths reconfigured nanorings, from top to bottom, respectively. Source data are provided as a Source Data file.

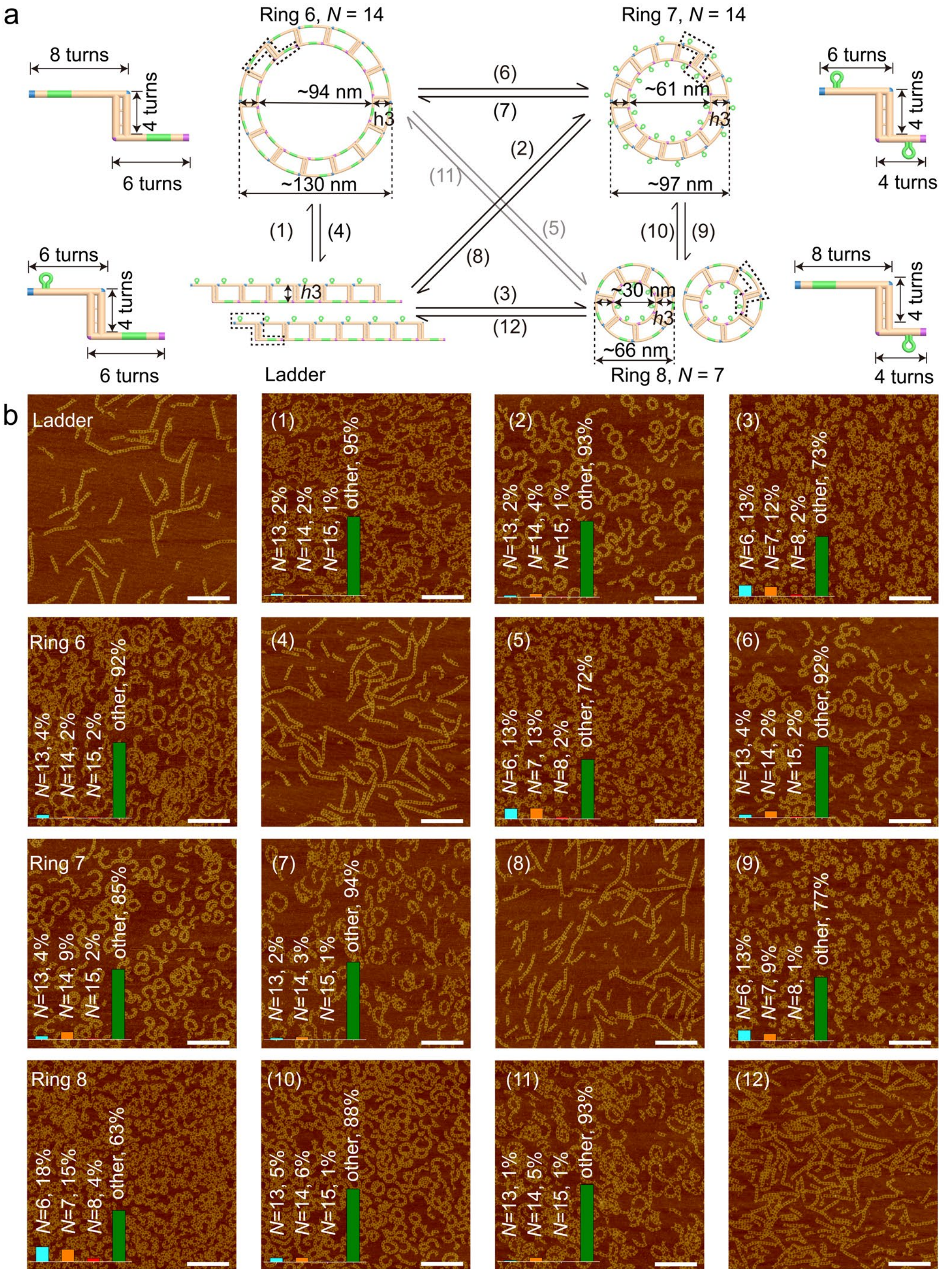

Supplementary Figure 47 | 4-states ring-to-ladder switching based on Z-shape TC tiles. a) Schematics of the one-one switching process between four rings, through a total of twelve paths. The four nanostructures are Ring 6 (N = 14), Ring 7 (N = 14), Ring 8 (N = 7), and a ladder. The four states TC tiles are Z-tile 8-4-6, Z-tile 6-4-4, Z-tile 8-4-4, and Z-tile 6-4-6. The twelve paths are labeled as (1) to (12), and the reconfigured direction are marked by arrow. b) The AFM images of nanorings, ladder, and the corresponding reconfigured nanostructures. Row 1 depicts the ladder reconfigured to three nanorings, from left to right, respectively. Row 2, row 3 and row 4 demonstrate the nine paths that with nanorings (Ring 6, Ring 7 or Ring 8) as initial states and then transfer to three different states, respectively. Image size: 1 μm × 1 μm, scale bar: 200 nm. Source data are provided as a Source Data file.

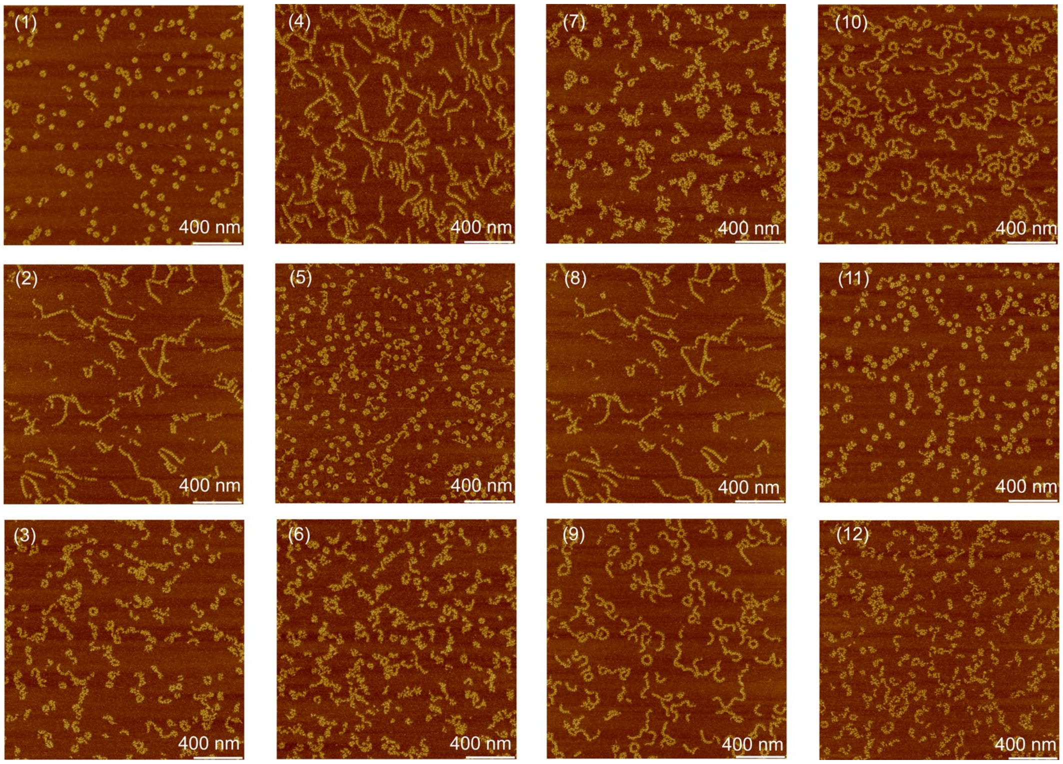

Supplementary Figure 48 | The AFM images of each path intermediates of pair 1 four states nanoring dynamic system. Intermediates formed via 12 paths are tested, including (1) to (12).

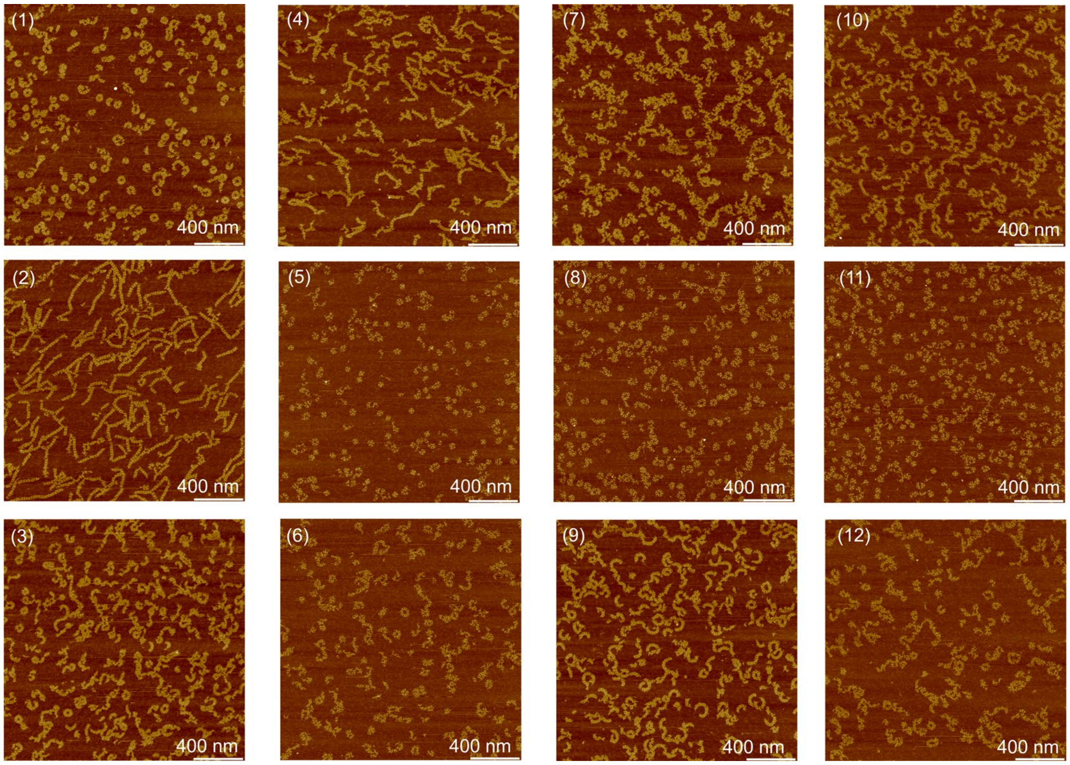

Supplementary Figure 49 | The AFM images of each path intermediates of pair 3 four states nanoring dynamic system. Intermediates formed via 12 paths are tested, including (1) to (12).

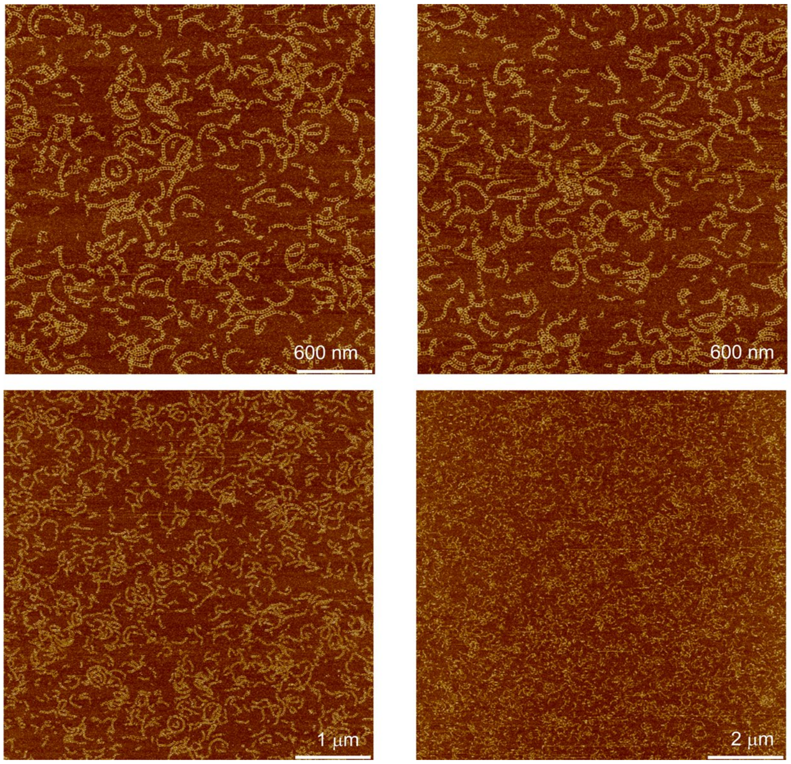

Supplementary Figure 50 | The AFM images of pair 1 ring 1 (N = 25). Monomer tile is TC-C-7-3.5-6. The assembly yield of the tile is not estimated due to the low yield. Source data are provided as a Source Data file.

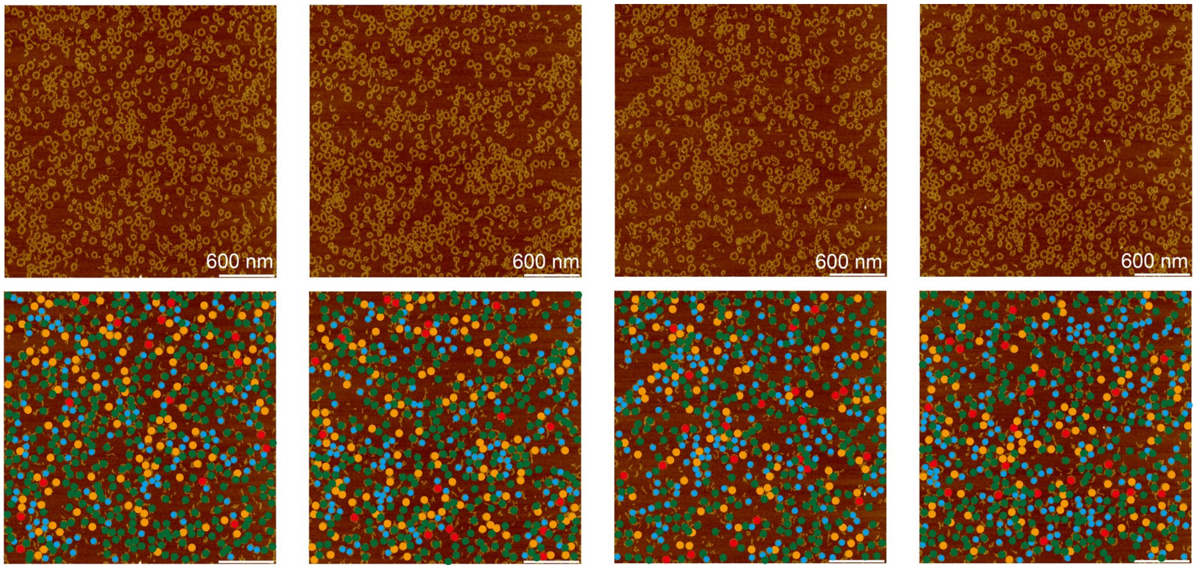

Supplementary Figure 51 | The estimated assembly yield of pair 1 ring 2 (N = 8). The Monomer tile is TC-C-9-3.5-6. Four of 3 μm ×3 μm AFM images are used for yield counting, with 2340 total counted nanorings (blue 625, yellow 400, red 80, green 1235). Source data are provided as a Source Data file.

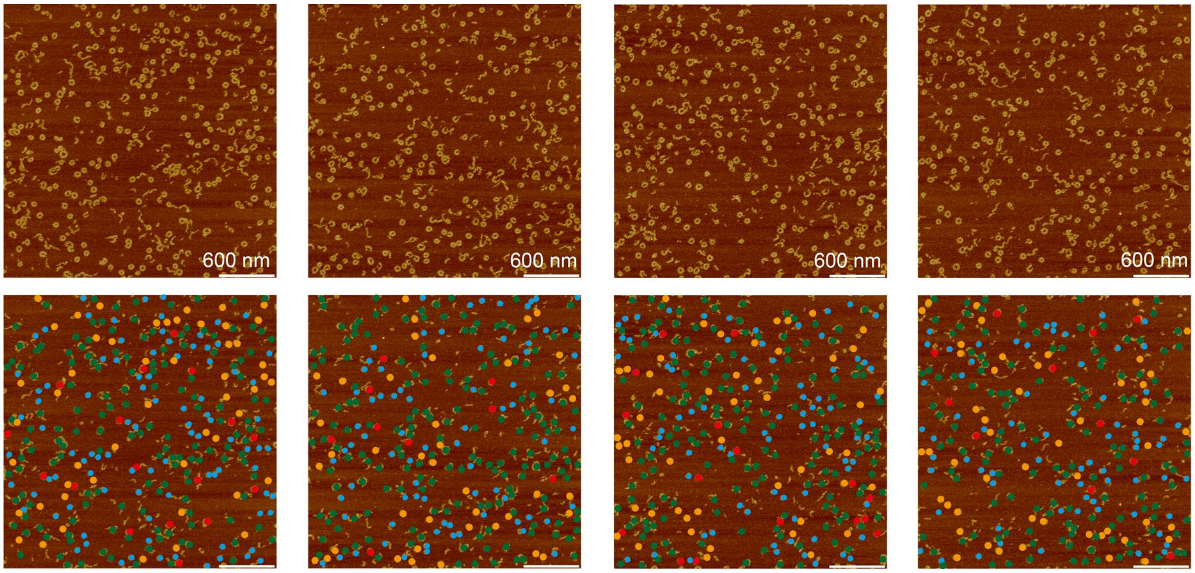

Supplementary Figure 52 | The estimated assembly yield of pair 1 ring 3 (*N* = 8). The Monomer tile is TC-C-7-3.5-4. Four of 3 μm ×3 μm AFM images are used for yield counting, with 1184 total counted nanorings (blue 320, yellow 193, red 53, green 618). Source data are provided as a Source Data file.

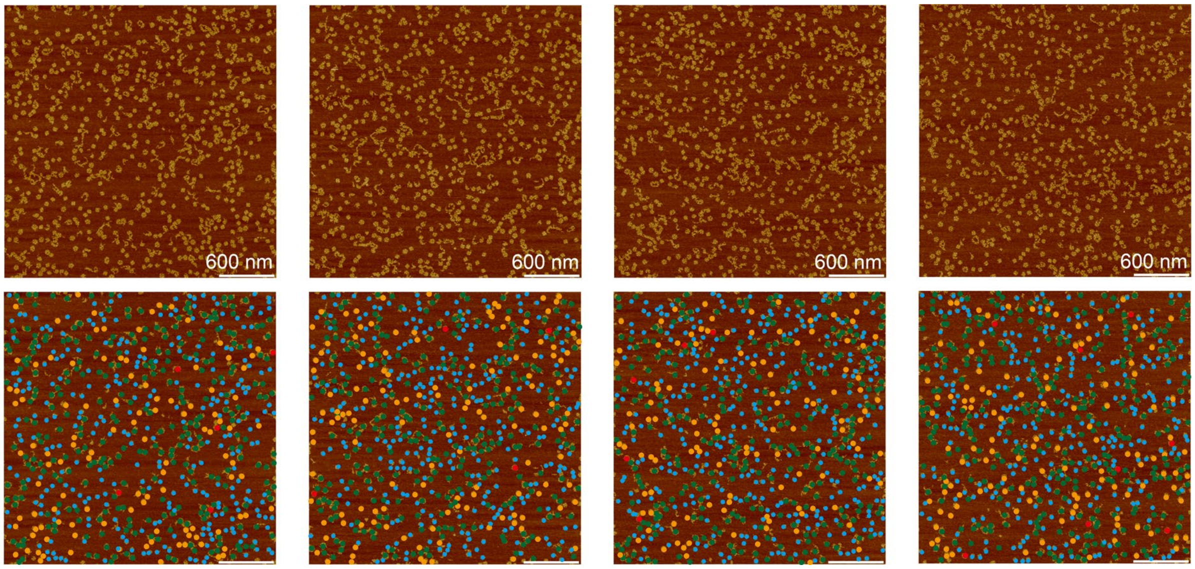

Supplementary Figure 53 | The estimated assembly yield of pair 1 ring 4 (*N* = 5). The Monomer tile is TC-C-9-3.5-4. Four of 3 μm ×3 μm AFM images are used for yield counting, with 2778 total counted nanorings (blue 1145, yellow 482, red 22, green 1129). Source data are provided as a Source Data file.

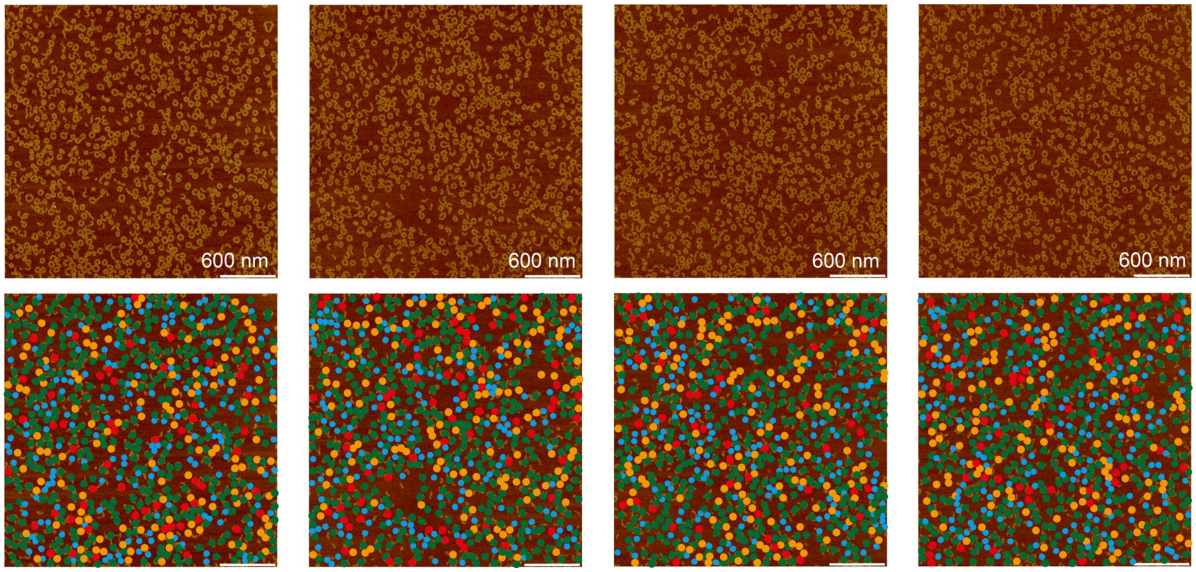

Supplementary Figure 54 | The estimated assembly yield of pair 1 path 1 (N = 8). Four of 3 μm ×3 μm AFM images are used for yield counting, with 3084 total counted nanorings (blue 693, yellow 563, red 216, green 1612). Source data are provided as a Source Data file.

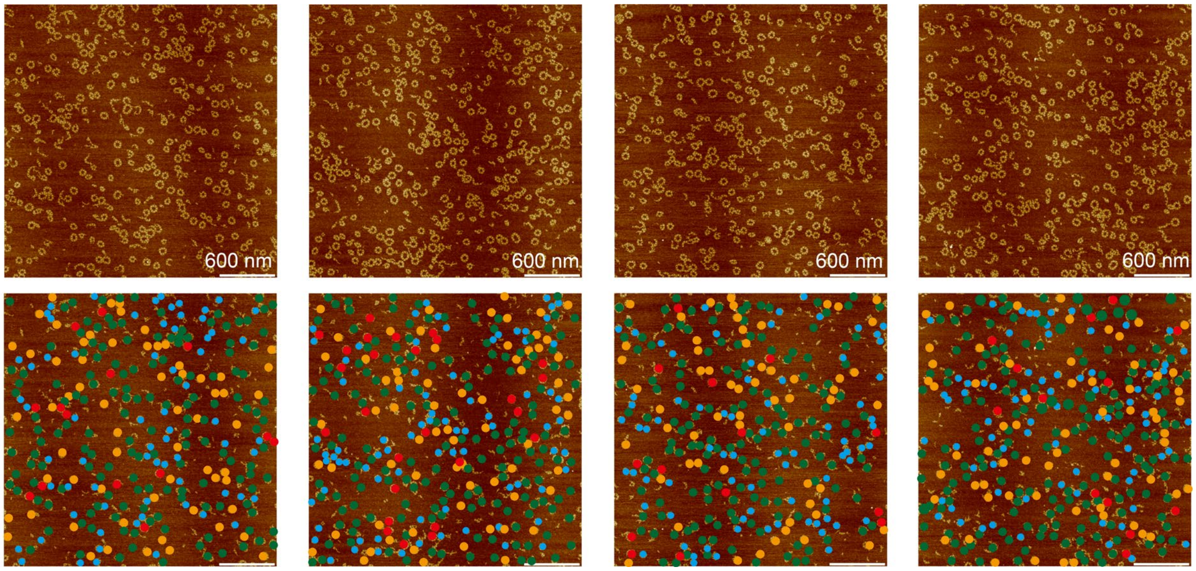

Supplementary Figure 55 | The estimated assembly yield of pair 1 path 2 (N = 8). Four of 3 μm ×3 μm AFM images are used for yield counting, with 2254 total counted nanorings (blue 299, yellow 633, red 67, green 1255). Source data are provided as a Source Data file.

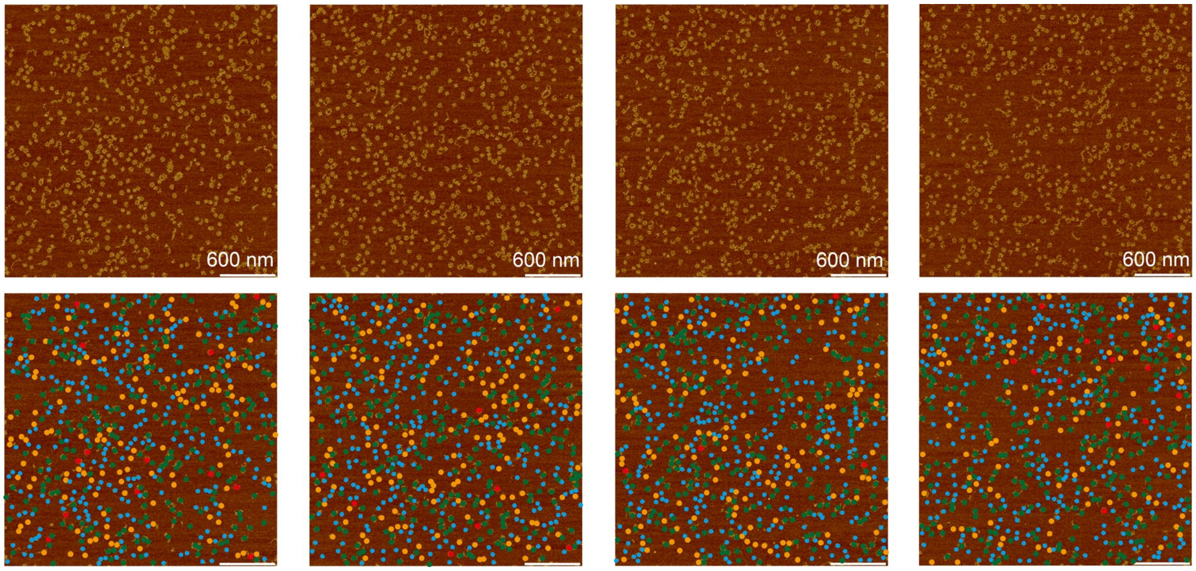

Supplementary Figure 56 | The estimated assembly yield of pair 1 path 3 (N = 5). Four of 3 μm ×3 μm AFM images are used for yield counting, with 2686 total counted nanorings (blue 1124, yellow 532, red 38, green 992). Source data are provided as a Source Data file.

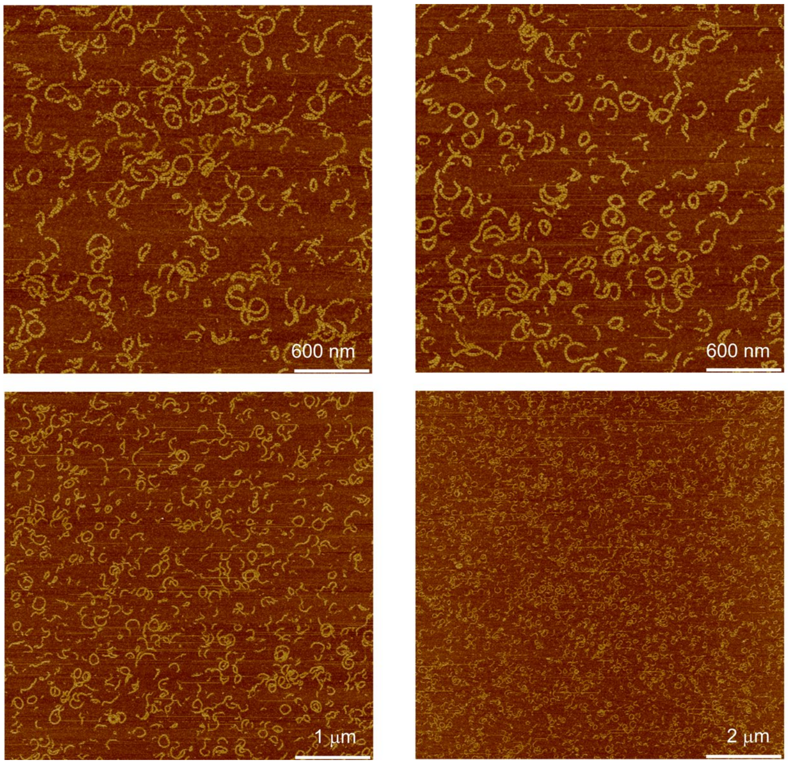

Supplementary Figure 57 | The AFM images of pair 1 path 4 (N = 25). The assembly yield of it is not estimated due to the low yield. Source data are provided as a Source Data file.

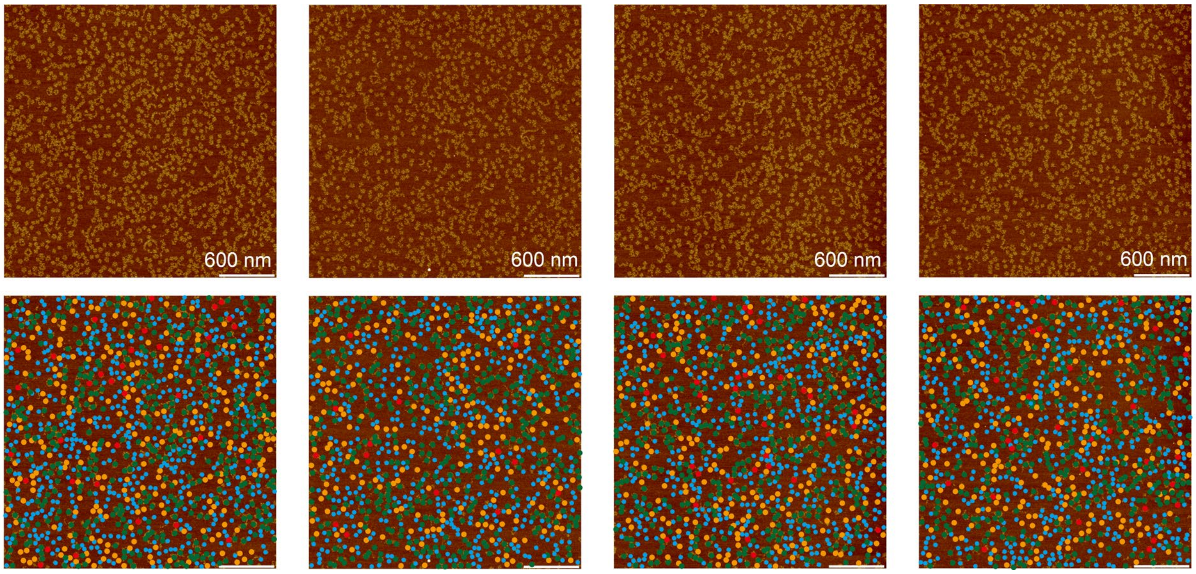

Supplementary Figure 58 | The estimated assembly yield of pair 1 path 5 (N = 5). Four of 3 μm ×3 μm AFM images are used for yield counting, with 4644 total counted nanorings (blue 1990, yellow 887, red 113, green 1654). Source data are provided as a Source Data file.

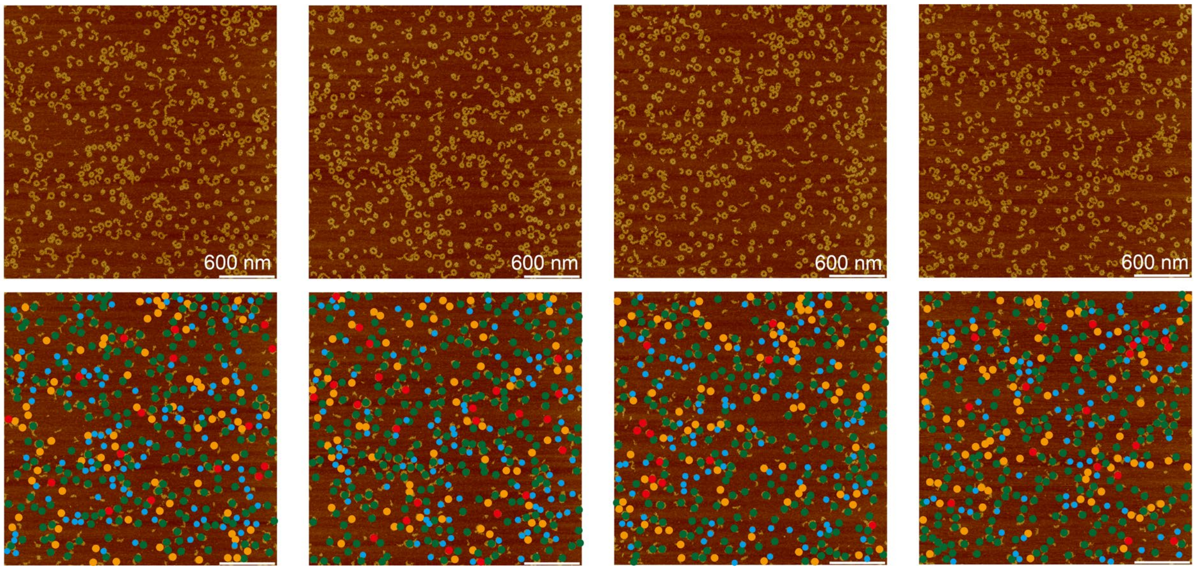

Supplementary Figure 59 | The estimated assembly yield of pair 1 path 6 (N = 8). Four of 3 μm ×3 μm AFM images are used for yield counting, with 1696 total counted nanorings (blue 380, yellow 304, red 71, green 941). Source data are provided as a Source Data file.

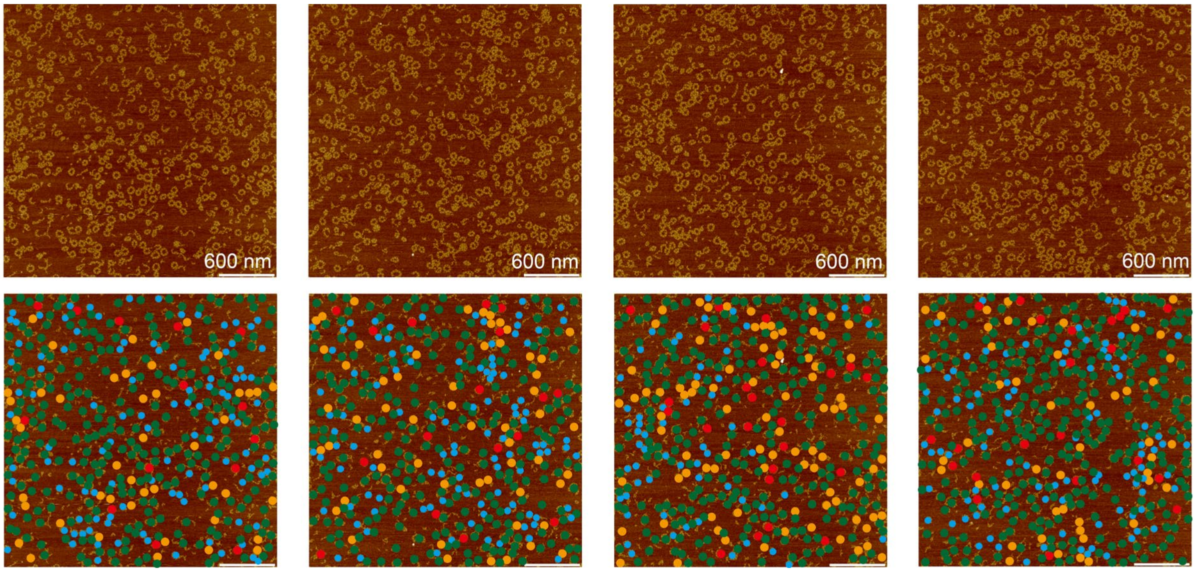

Supplementary Figure 60 | The estimated assembly yield of pair 1 path 7 (N = 8). Four of 3 μm ×3 μm AFM images are used for yield counting, with 1770 total counted nanorings (blue 369, yellow 239, red 77, green 1085). Source data are provided as a Source Data file.

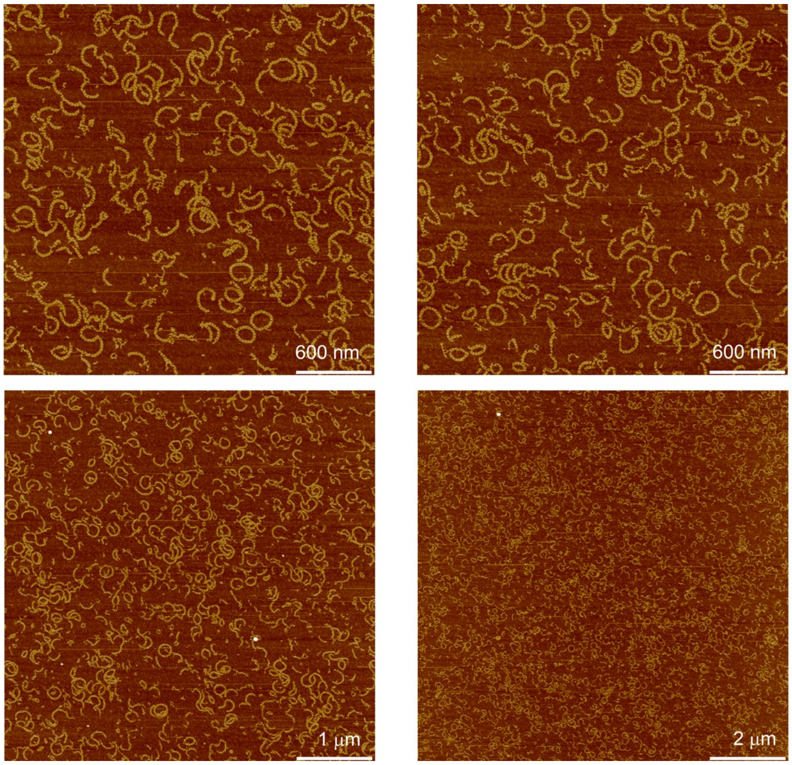

Supplementary Figure 61 | The AFM images of pair 1 path 8 (N = 25). The assembly yield of it is not estimated due to the low yield. Source data are provided as a Source Data file.

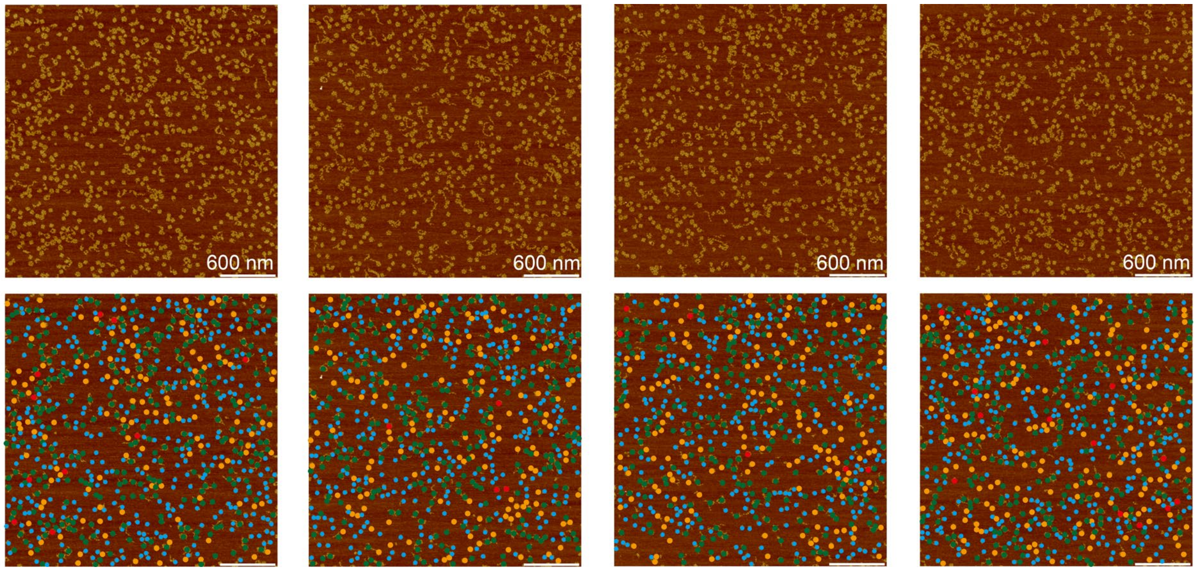

Supplementary Figure 62 | The estimated assembly yield of pair 1 path 9 (N = 5). Four of 3 μm ×3 μm AFM images are used for yield counting, with 2938 total counted nanorings (blue 1251, yellow 580, red 34, green 1073). Source data are provided as a Source Data file.

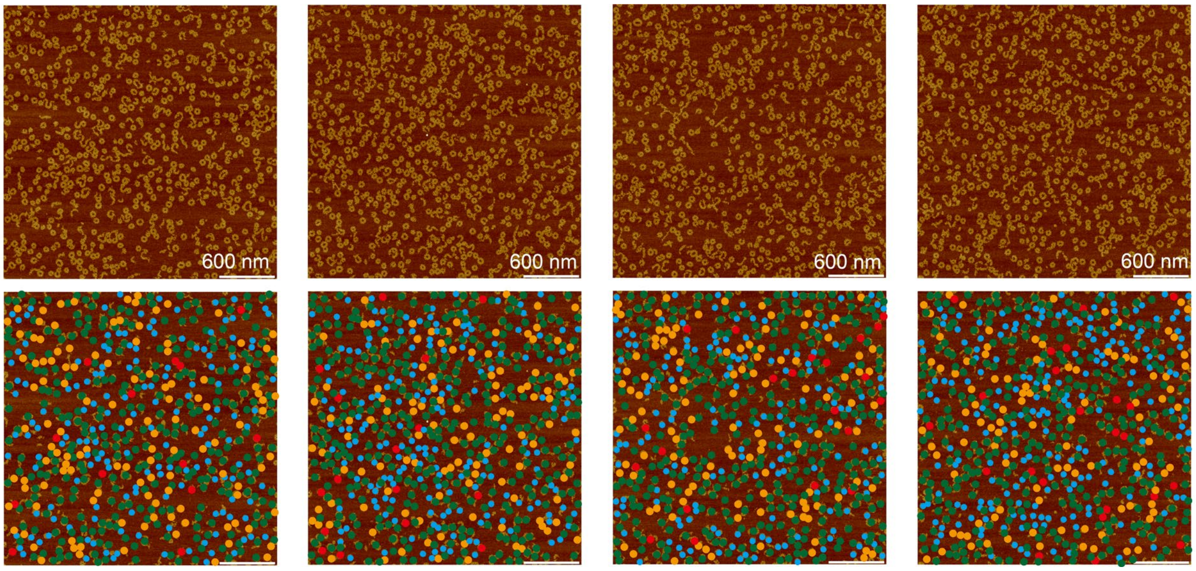

Supplementary Figure 63 | The estimated assembly yield of pair 1 path 10 (N = 8). Four of 3 μm ×3 μm AFM images are used for yield counting, with 2510 total counted nanorings (blue 697, yellow 453, red 73, green 1287). Source data are provided as a Source Data file.

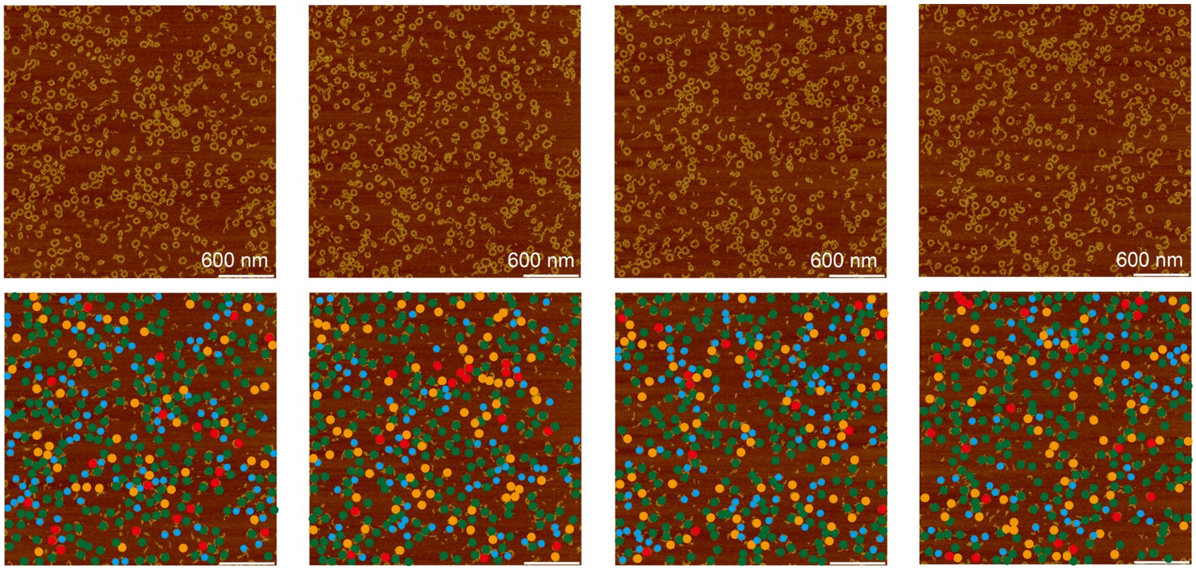

Supplementary Figure 64 | The estimated assembly yield of pair 1 path 11 (N = 8). Four of 3 μm ×3 μm AFM images are used for yield counting, with 1536 total counted nanorings (blue 328, yellow 240, red 75, green 893). Source data are provided as a Source Data file.

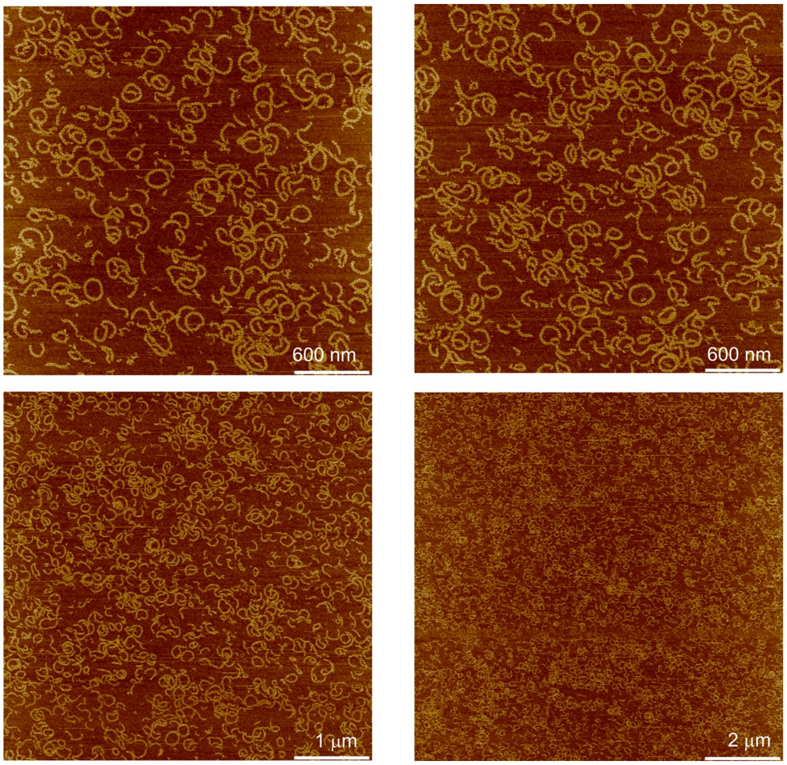

Supplementary Figure 65 | The AFM images of pair 1 path 12 (N = 25). The assembly yield of it is not estimated due to the low yield. Source data are provided as a Source Data file.

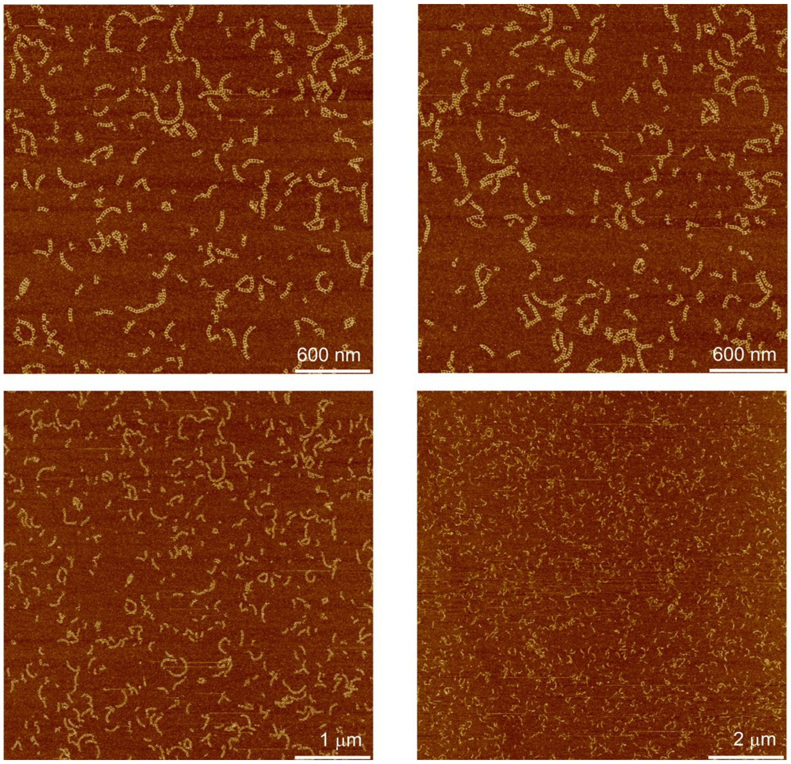

Supplementary Figure 66 | The AFM images of pair 2 ring 1* (N = 38). The monomer tile is TC-C-7-5.5-6. The assembly yield of it is not estimated due to the low yield. Source data are provided as a Source Data file.

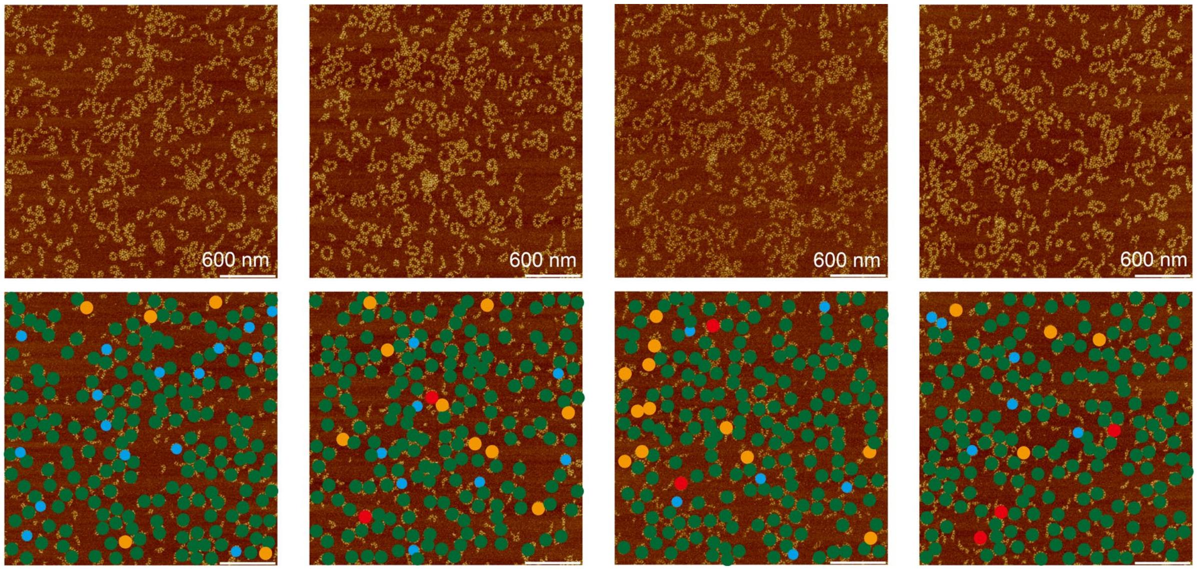

Supplementary Figure 67 | The estimated assembly yield of pair 2 ring 2* (N = 12). The monomer tile is TC-C-9-5.5-6. Four of 3 μm ×3 μm AFM images are used for yield counting, with 790 total counted nanorings (blue 36, yellow 30, red 7, green 717). Source data are provided as a Source Data file.

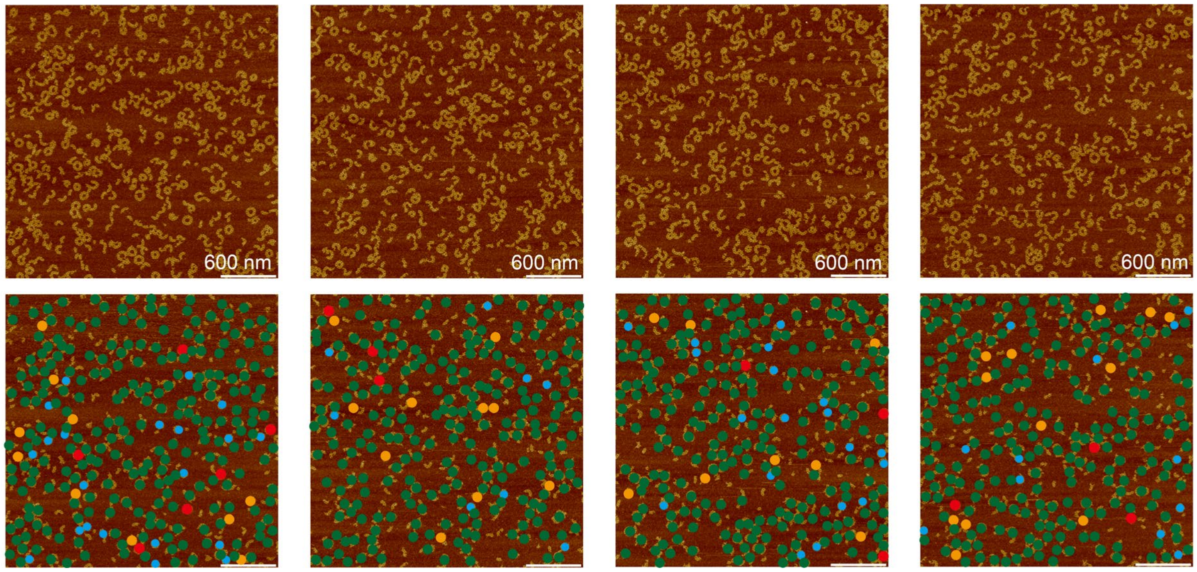

Supplementary Figure 68 | The estimated assembly yield of pair 2 ring 3* (*N* = 12). The monomer tile is TC-C-7-5.5-4. Four of 3 μm ×3 μm AFM images are used for yield counting, with 1040 total counted nanorings (blue 60, yellow 40, red 15, green 925). Source data are provided as a Source Data file.

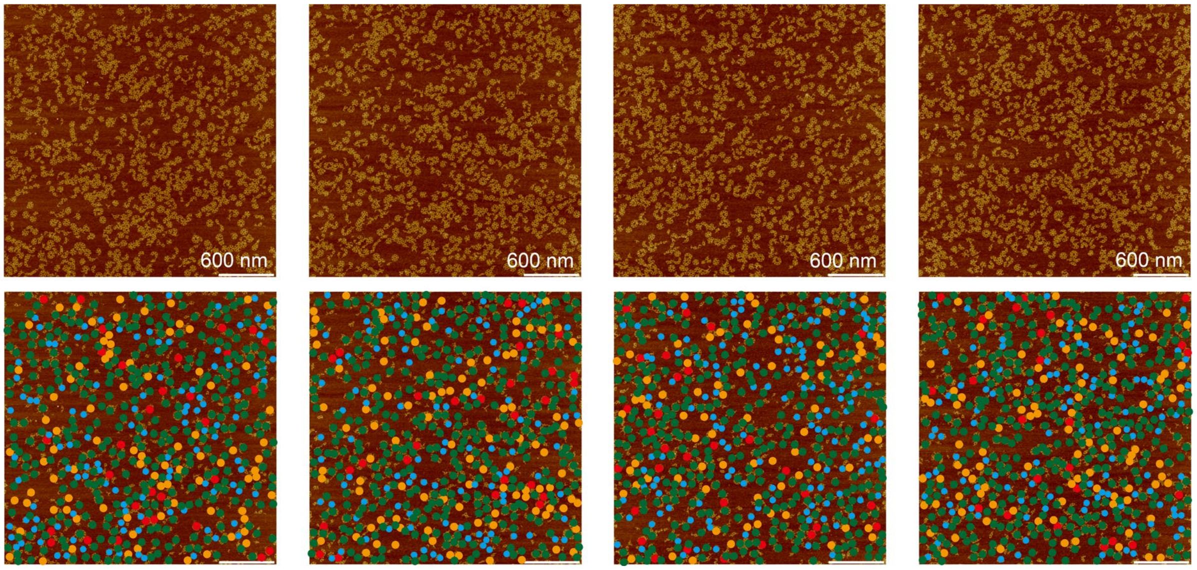

Supplementary Figure 69 | The estimated assembly yield of pair 2 ring 4* (*N* = 7). The monomer tile is TC-C-9-5.5-4. Four of 3 μm ×3 μm AFM images are used for yield counting, with 2335 total counted nanorings (blue 468, yellow 355, red 111, green 1401). Source data are provided as a Source Data file.

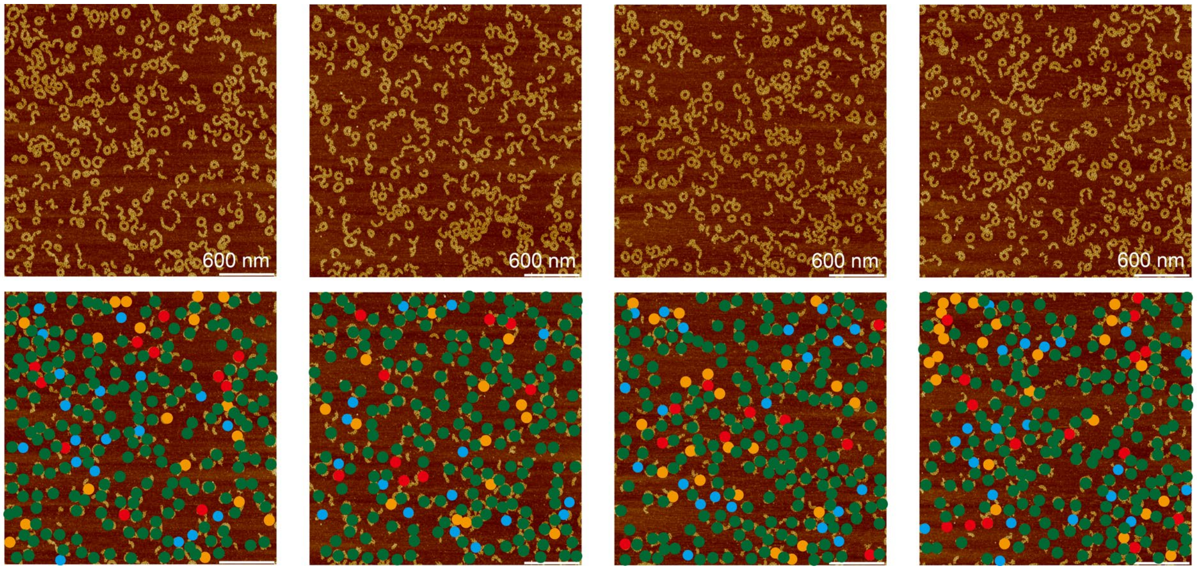

Supplementary Figure 70 | The estimated assembly yield of pair 2 path 1 (*N* = 12). Four of 3 μm ×3 μm AFM images are used for yield counting, with 983 total counted nanorings (blue 77, yellow 79, red 45, green 782). Source data are provided as a Source Data file.

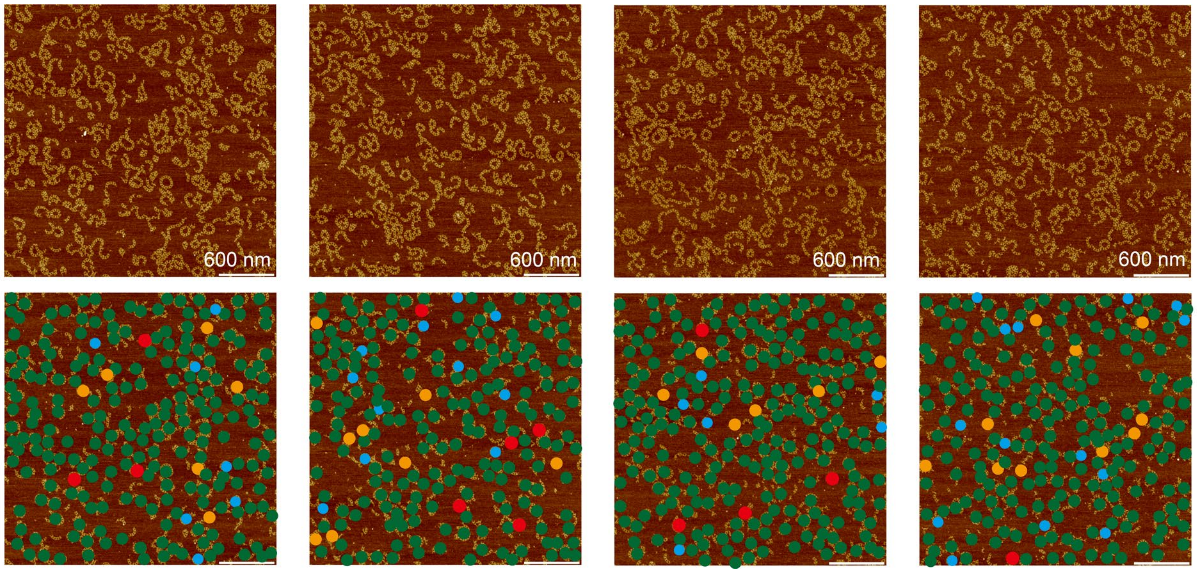

Supplementary Figure 71 | The estimated assembly yield of pair 2 ring 2* (*N* = 12). Four of 3 μm ×3 μm AFM images are used for yield counting, with 911 total counted nanorings (blue 38, yellow 30, red 12, green 831). Source data are provided as a Source Data file.

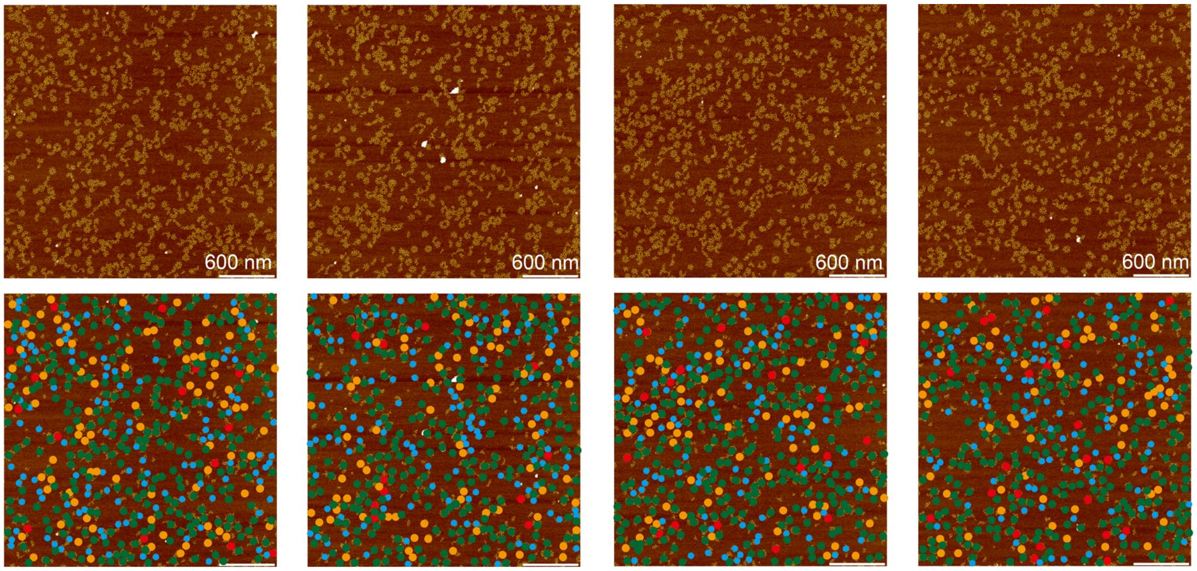

Supplementary Figure 72 | The estimated assembly yield of pair 2 ring 1* (*N* = 7). Four of 3 μm ×3 μm AFM images are used for yield counting, with 1911 total counted nanorings (blue 433, yellow 303, red 68, green 1107). Source data are provided as a Source Data file.

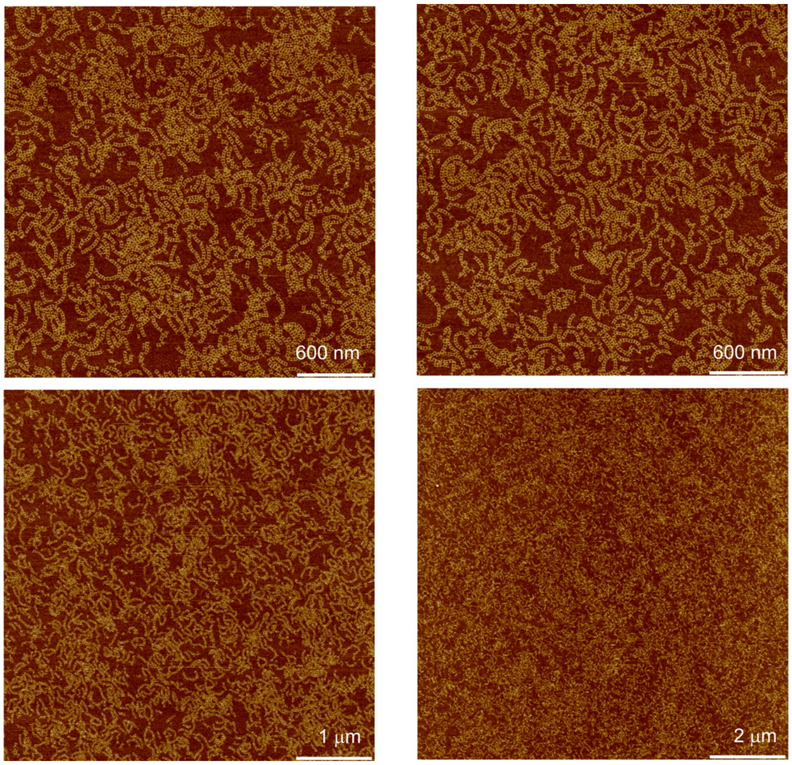

Supplementary Figure 73 | The AFM images of pair 2 path 4 (*N* = 38). The assembly yield of it is not estimated due to the low yield. Source data are provided as a Source Data file.

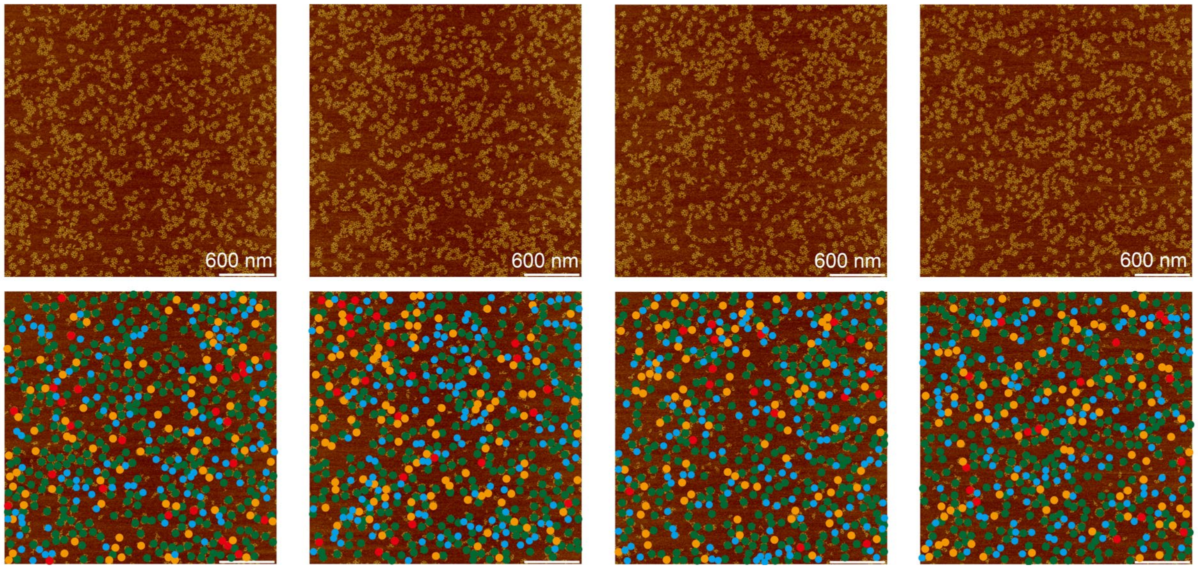

Supplementary Figure 74 | The estimated assembly yield of pair 2 path 5 (N = 7). Four of 3 µm ×3 µm AFM images are used for yield counting, with 2349 total counted nanorings (blue 535, yellow 373, red 83, green 1358). Source data are provided as a Source Data file.

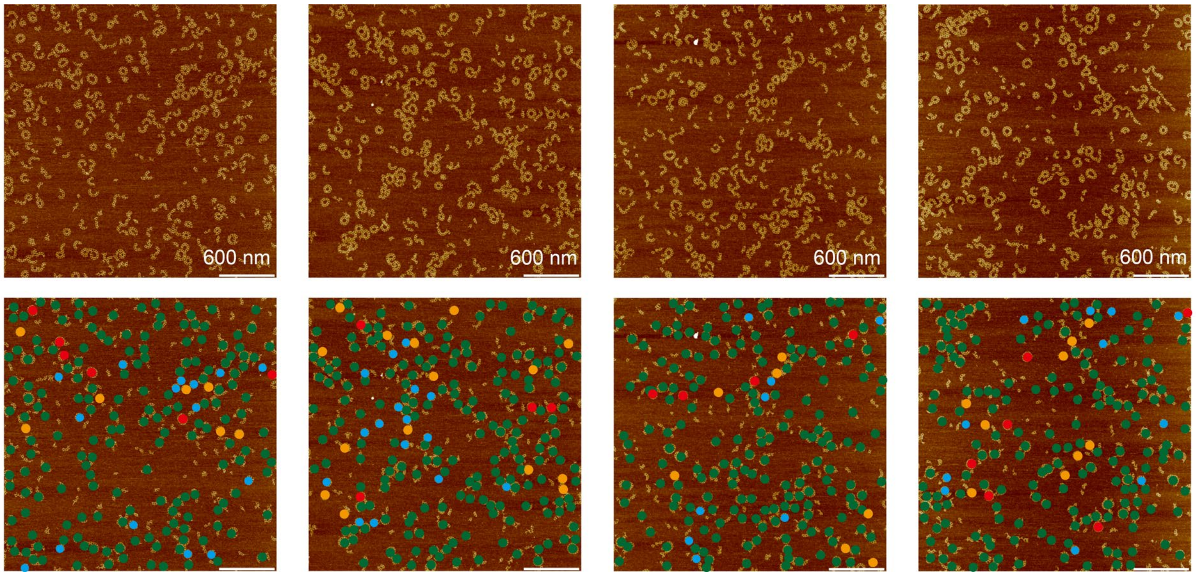

Supplementary Figure 75 | The estimated assembly yield of pair 2 path 6 (N = 12). Four of 3 µm ×3 µm AFM images are used for yield counting, with 830 total counted nanorings (blue 50, yellow 38, red 20, green 722). Source data are provided as a Source Data file.

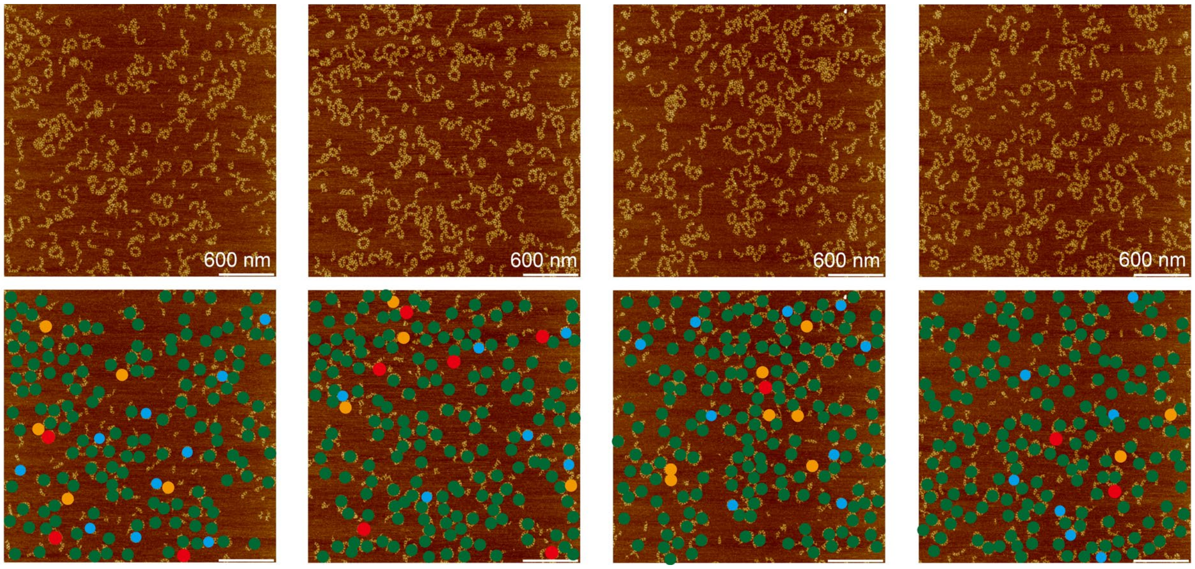

Supplementary Figure 76 | The estimated assembly yield of pair 2 path 7 (N = 12). Four of 3 μm ×3 μm AFM images are used for yield counting, with 714 total counted nanorings (blue 32, yellow 18, red 12, green 652). Source data are provided as a Source Data file.

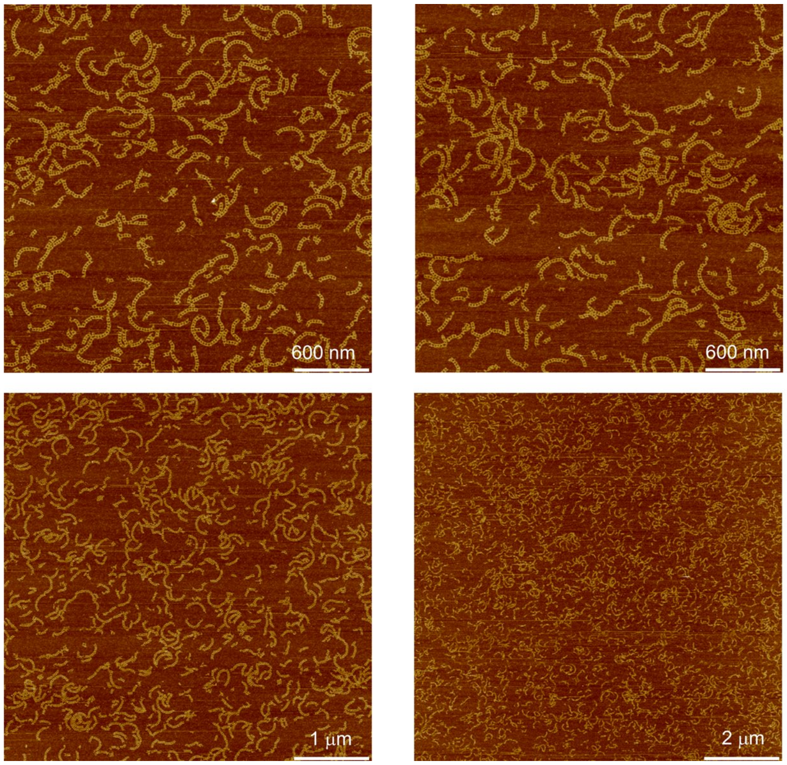

Supplementary Figure 77 | The AFM images of pair 2 path 8 (N = 38). The assembly yield of it is not estimated due to the low yield. Source data are provided as a Source Data file.

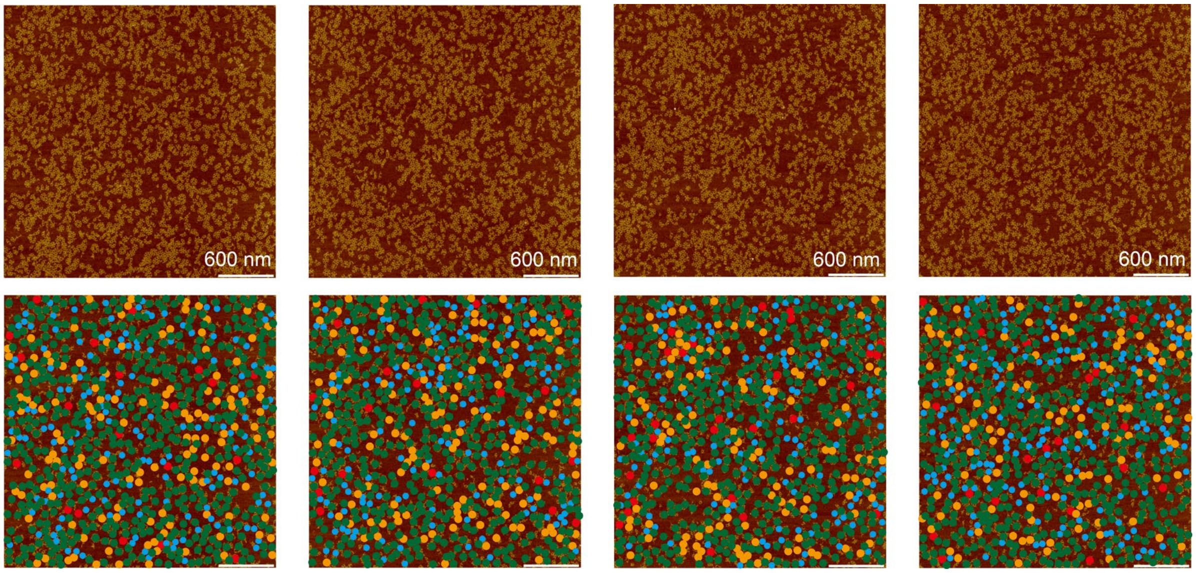

Supplementary Figure 78 | The estimated assembly yield of pair 2 path 9 (*N*=7). Four of 3 μm ×3 μm AFM images are used for yield counting, with 3182 total counted nanorings (blue 610, yellow 504, red 87, green 1981). Source data are provided as a Source Data file.

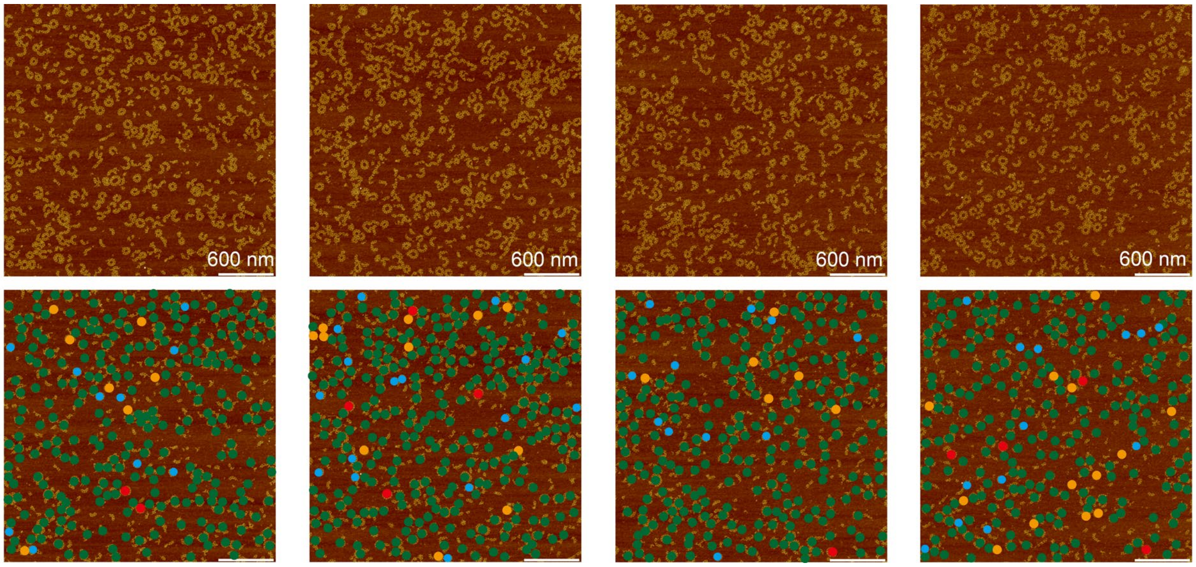

Supplementary Figure 79 | The estimated assembly yield of pair 2 path 10 (*N*=12). Four of 3 μm ×3 μm AFM images are used for yield counting, with 1138 total counted nanorings (blue 50, yellow 37, red 11, green 1040). Source data are provided as a Source Data file.

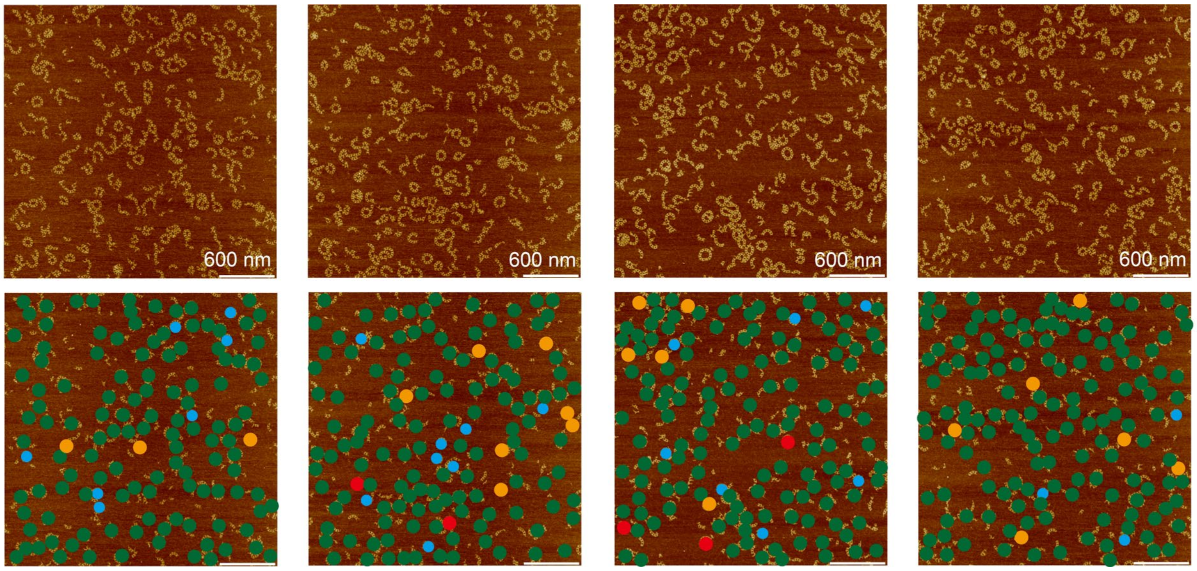

Supplementary Figure 80 | The estimated assembly yield of pair 2 path 11 (*N*=12). Four of 3 µm ×3 µm AFM images are used for yield counting, with 564 total counted nanorings (blue 25, yellow 21, red 5, green 513). Source data are provided as a Source Data file.

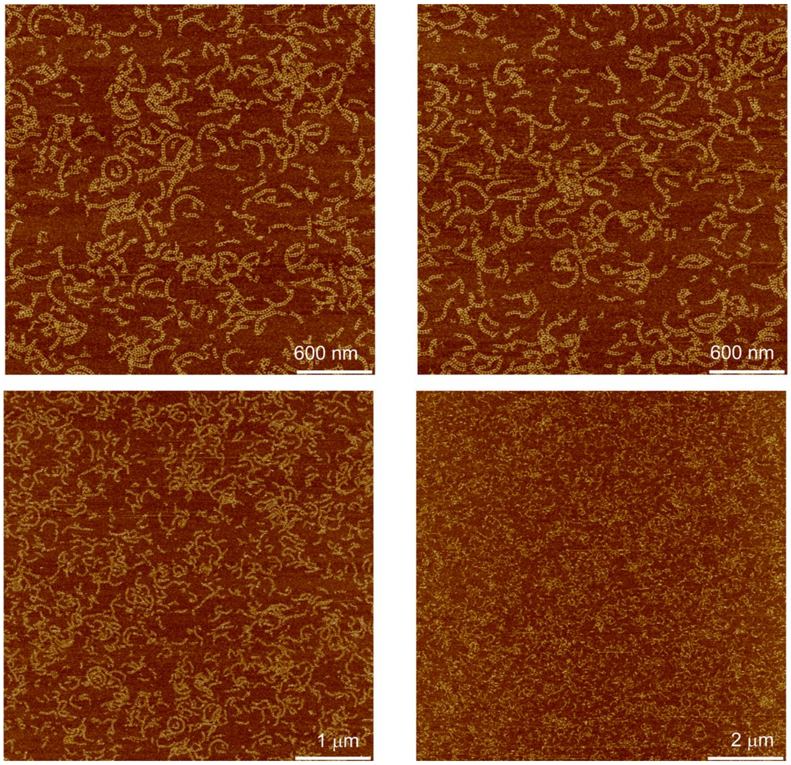

Supplementary Figure 81 | The AFM images of pair 2 path 12 (*N* = 38). The assembly yield of it is not estimated due to the low yield. Source data are provided as a Source Data file.

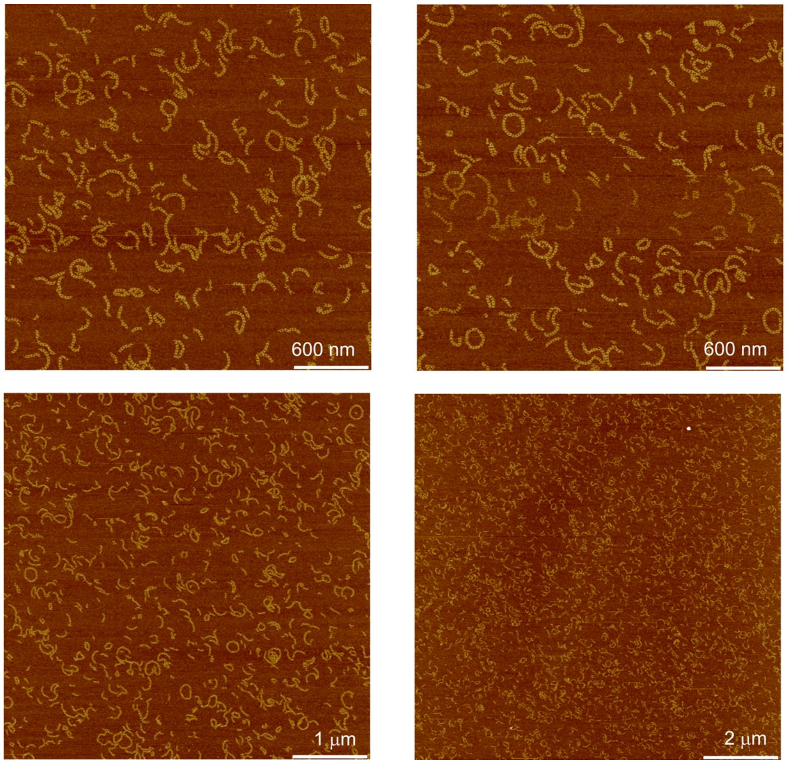

Supplementary Figure 82 | The AFM images of pair 3 ring 5 (N = 28). The monomer tile is TC-Z-7-4-6. The assembly yield of it is not estimated due to the low yield. Source data are provided as a Source Data file.

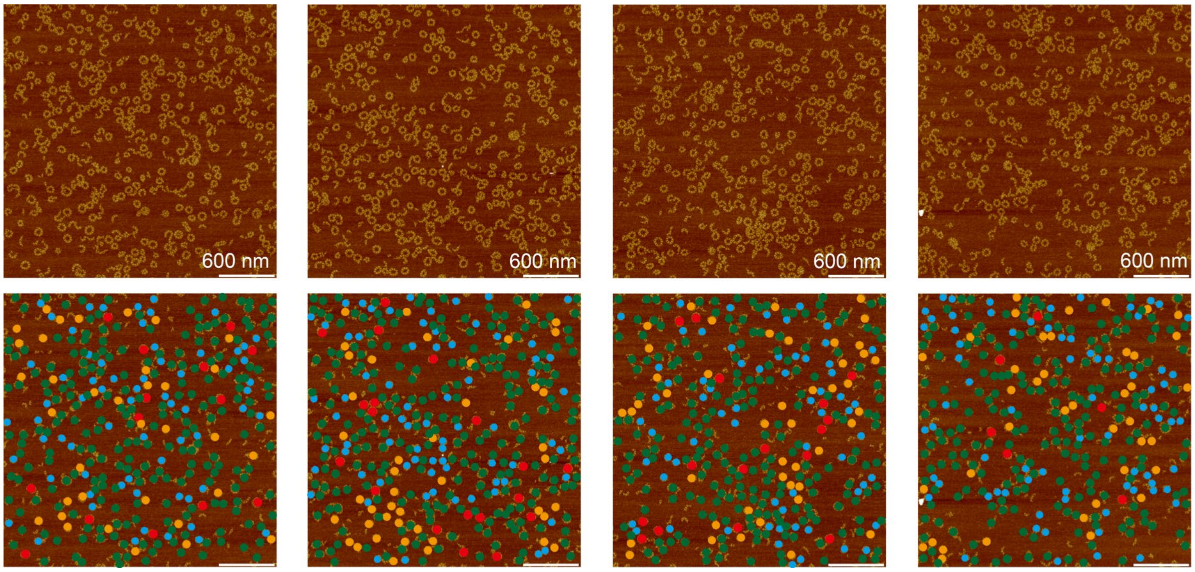

Supplementary Figure 83 | The estimated assembly yield of pair 3 ring 6 (N = 9). The monomer tile is TC-Z-9-4-6. Four of 3 μm ×3 μm AFM images are used for yield counting, with 1371 total counted nanorings (blue 281, yellow 165, red 55, green 870). Source data are provided as a Source Data file.

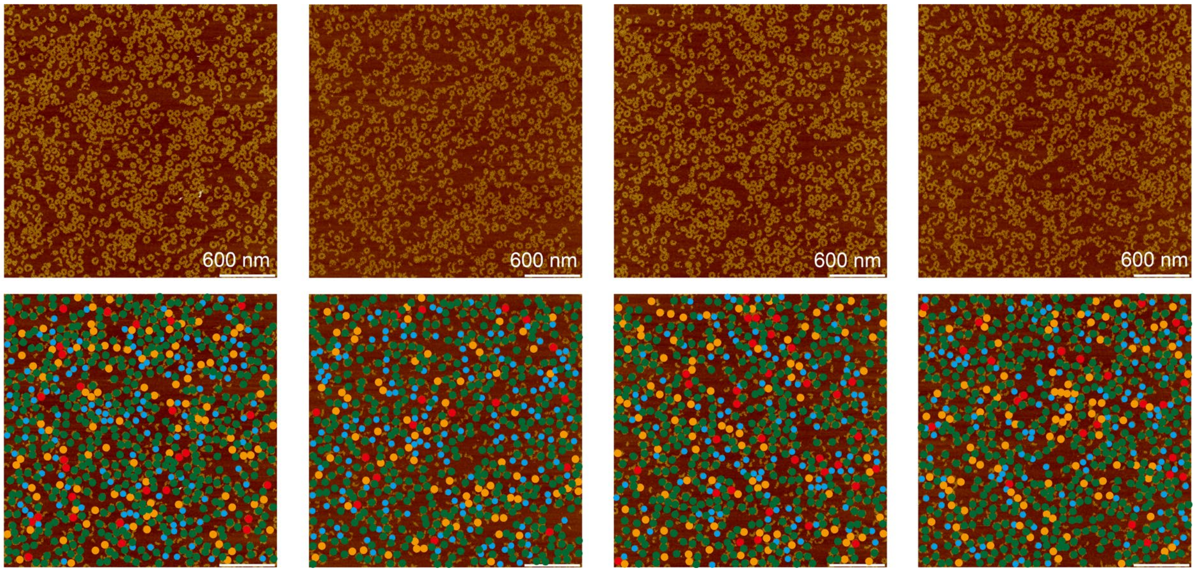

Supplementary Figure 84 | The estimated assembly yield of pair 3 ring 7 (N = 9). The monomer tile is TC-Z-7-4-4. Four of 3 μm ×3 μm AFM images are used for yield counting, with 2780 total counted nanorings (blue 533, yellow 368, red 102, green 1777). Source data are provided as a Source Data file.

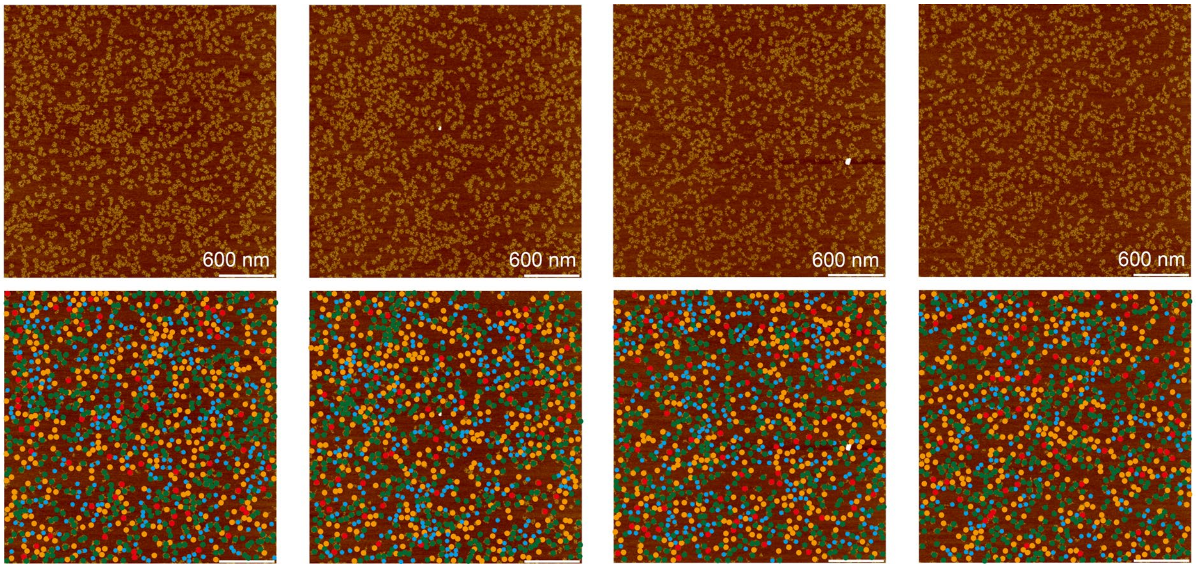

Supplementary Figure 85 | The estimated assembly yield of pair 3 ring 8 (N = 7). The monomer tile is TC-Z-9-4-4. Four of 3 μm ×3 μm AFM images are used for yield counting, with 4554 total counted nanorings (blue 1019, yellow 1333, red 252, green 1950). Source data are provided as a Source Data file.

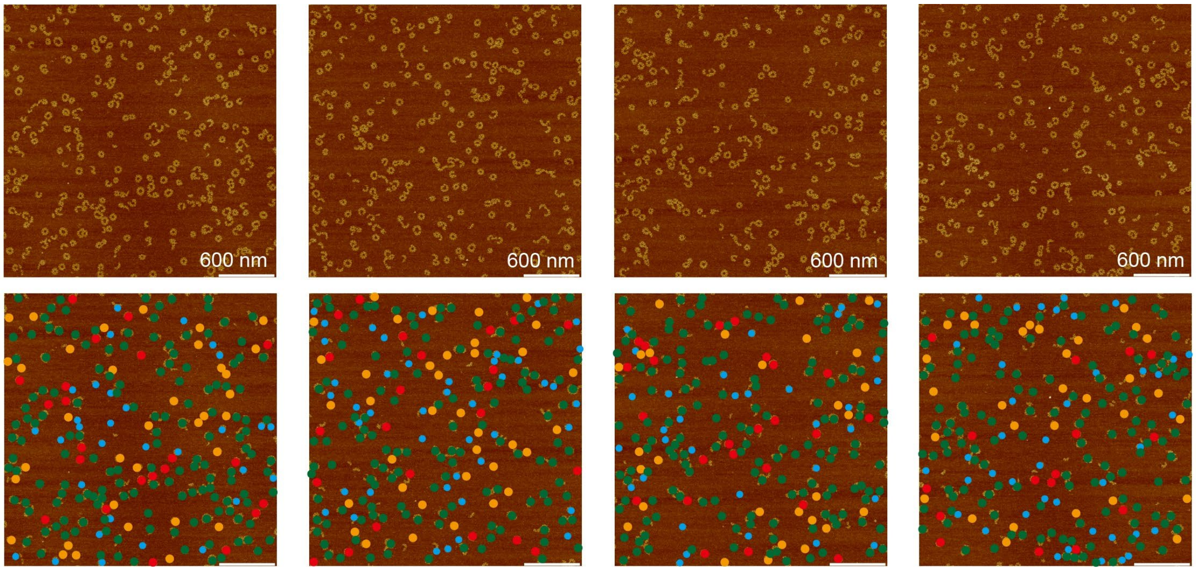

Supplementary Figure 86 | The estimated assembly yield of pair 3 path 1 (N = 9). Four of 3 μm ×3 μm AFM images are used for yield counting, with 934 total counted nanorings (blue 149, yellow 131, red 85, green 569). Source data are provided as a Source Data file.

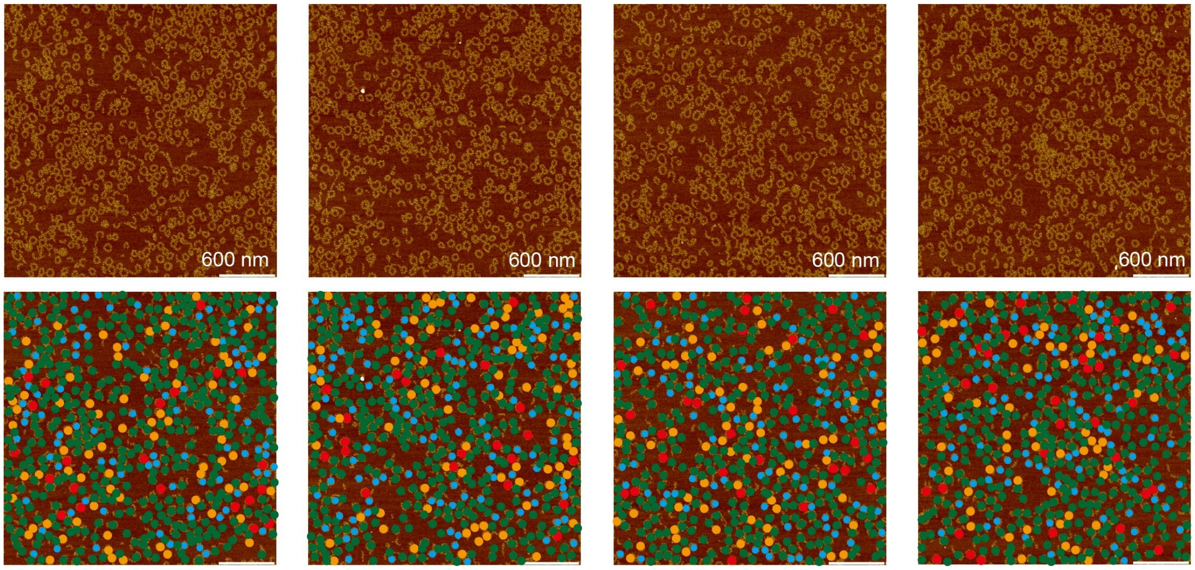

Supplementary Figure 87 | The estimated assembly yield of pair 3 path 2 (N = 9). Four of 3 μm ×3 μm AFM images are used for yield counting, with 2252 total counted nanorings (blue 407, yellow 278, red 104, green 1463). Source data are provided as a Source Data file.

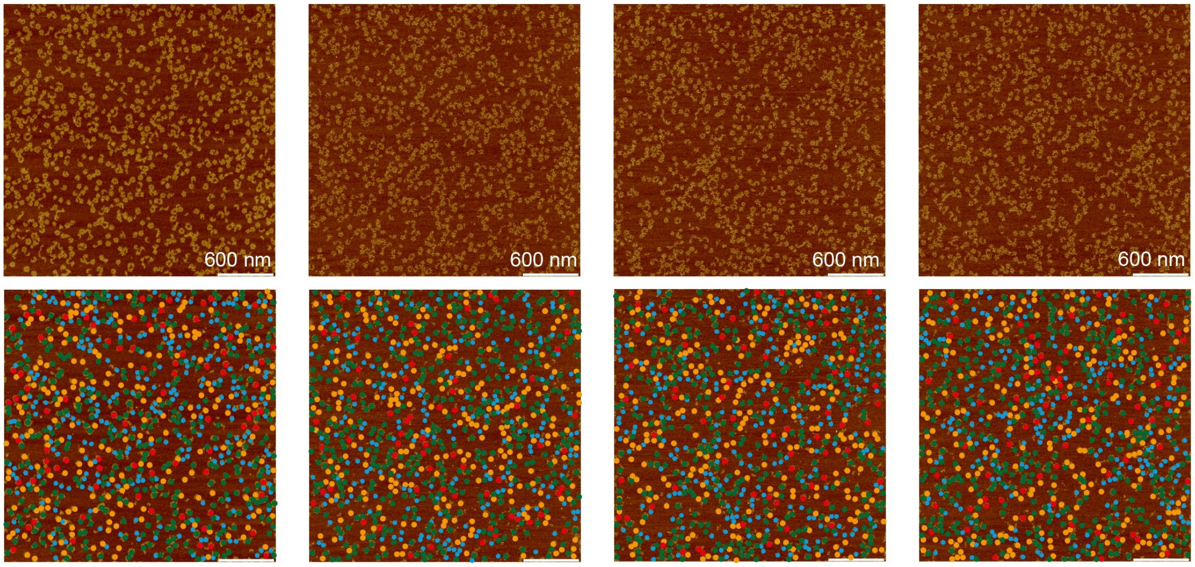

Supplementary Figure 88 | The estimated assembly yield of pair 3 path 3 (N = 7). Four of 3 μm ×3 μm AFM images are used for yield counting, with 3598 total counted nanorings (blue 953, yellow 908, red 279, green 1458). Source data are provided as a Source Data file.

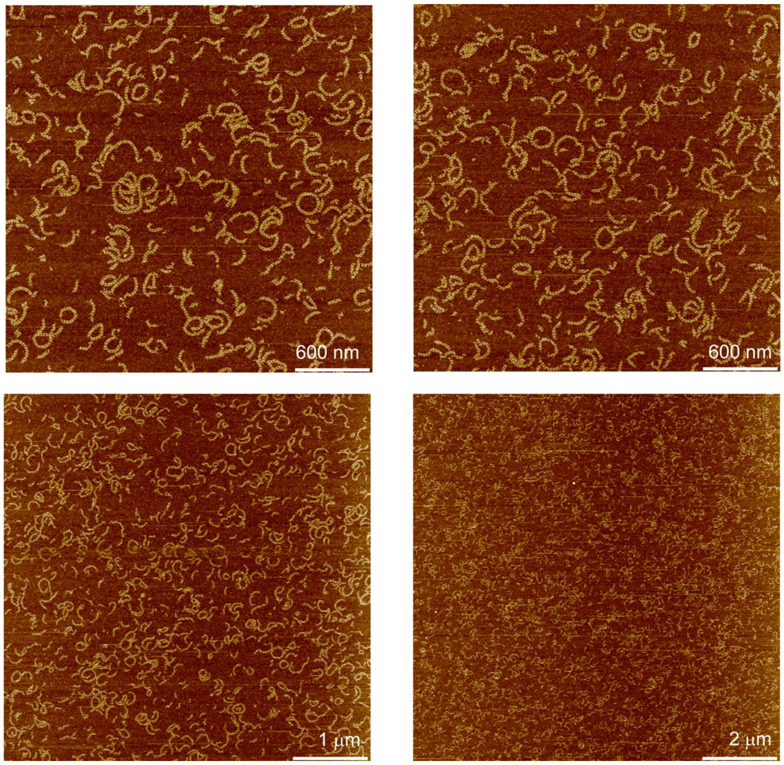

Supplementary Figure 89 | The AFM images of pair 3 path 4 (N = 28). The assembly yield of it is not estimated due to the low yield. Source data are provided as a Source Data file.

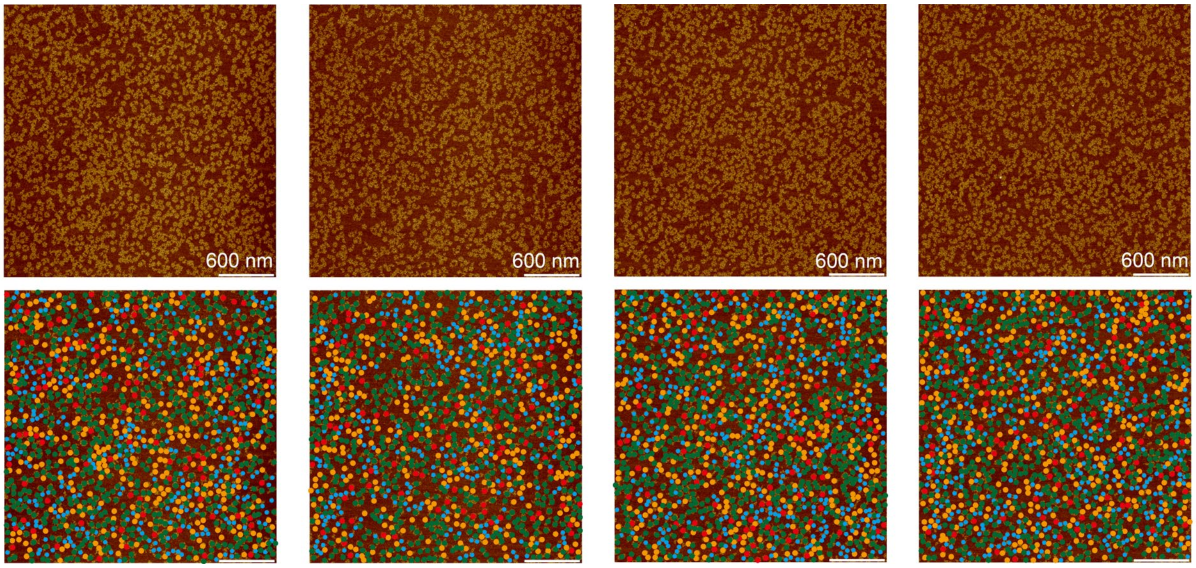

Supplementary Figure 90 | The estimated assembly yield of pair 3 path 5 (N = 5). Four of 3 μm ×3 μm AFM images are used for yield counting, with 5702 total counted nanorings (blue 1305, yellow 1364, red 390, green 2643). Source data are provided as a Source Data file.

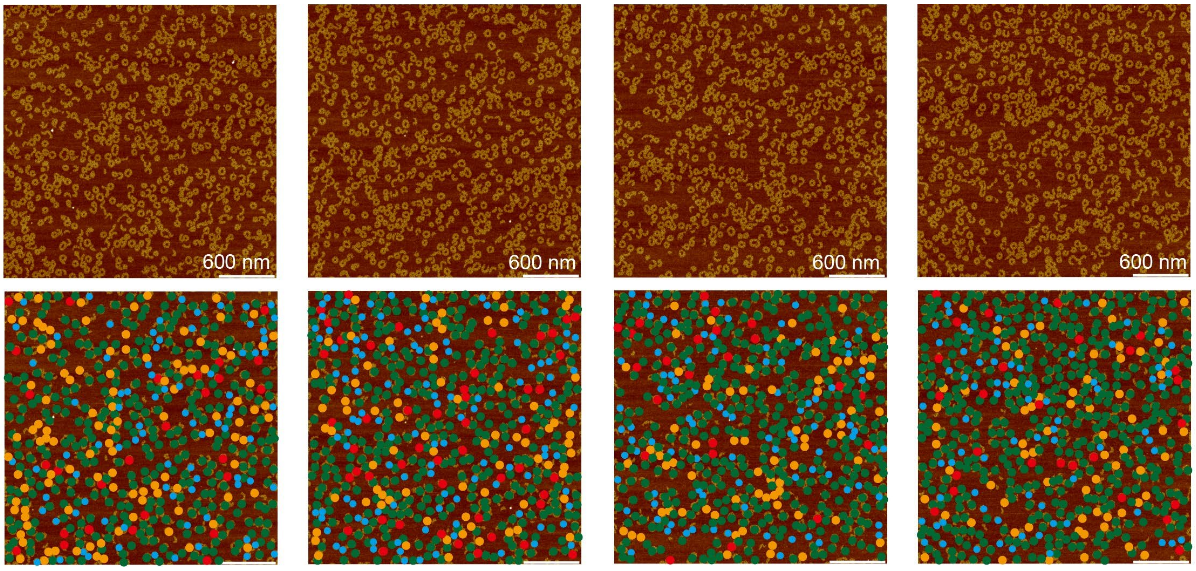

Supplementary Figure 91 | The estimated assembly yield of pair 3 path 6 (N = 9). Four of 3 μm ×3 μm AFM images are used for yield counting, with 2303 total counted nanorings (blue 367, yellow 328, red 126, green 1482). Source data are provided as a Source Data file.

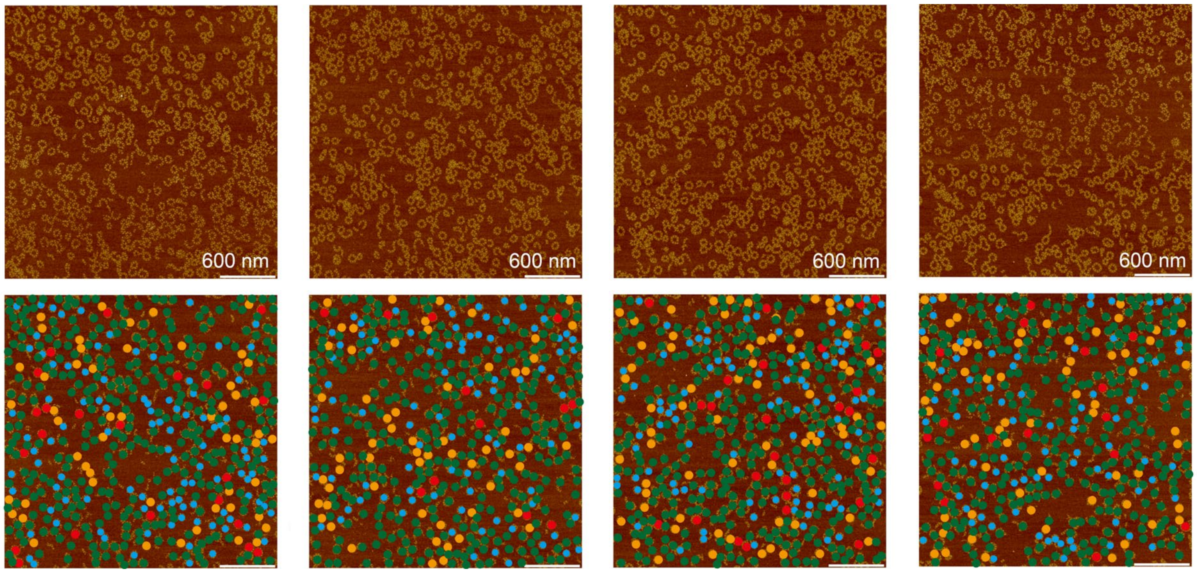

Supplementary Figure 92 | The estimated assembly yield of pair 3 path 7 (N = 9). Four of 3 μm ×3 μm AFM images are used for yield counting, with 1994 total counted nanorings (blue 366, yellow 249, red 77, green 1302). Source data are provided as a Source Data file.

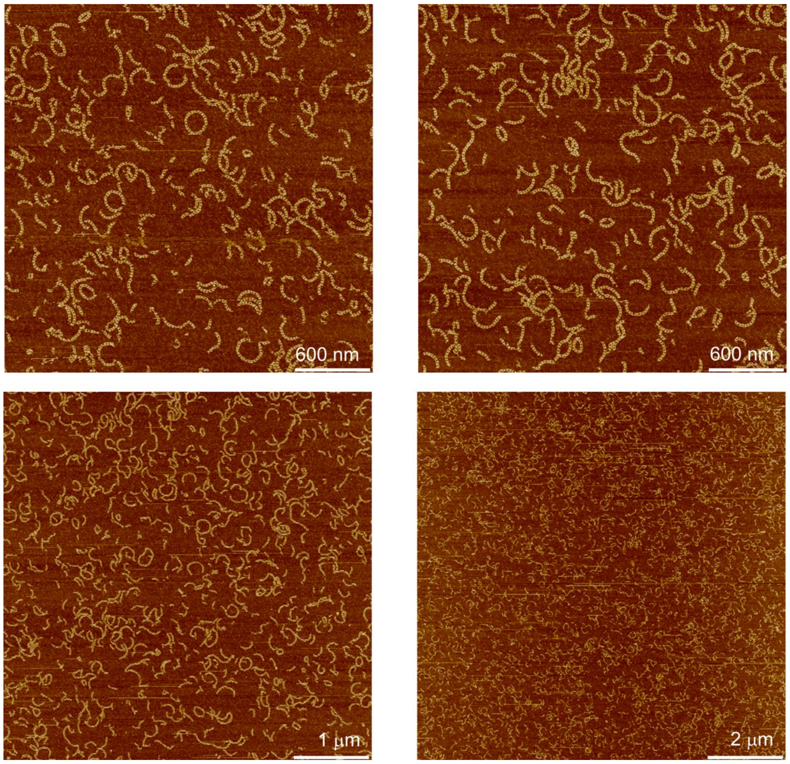

Supplementary Figure 93 | The AFM images of pair 3 path 8 (N = 28). The assembly yield of it is not estimated due to the low yield. Source data are provided as a Source Data file.

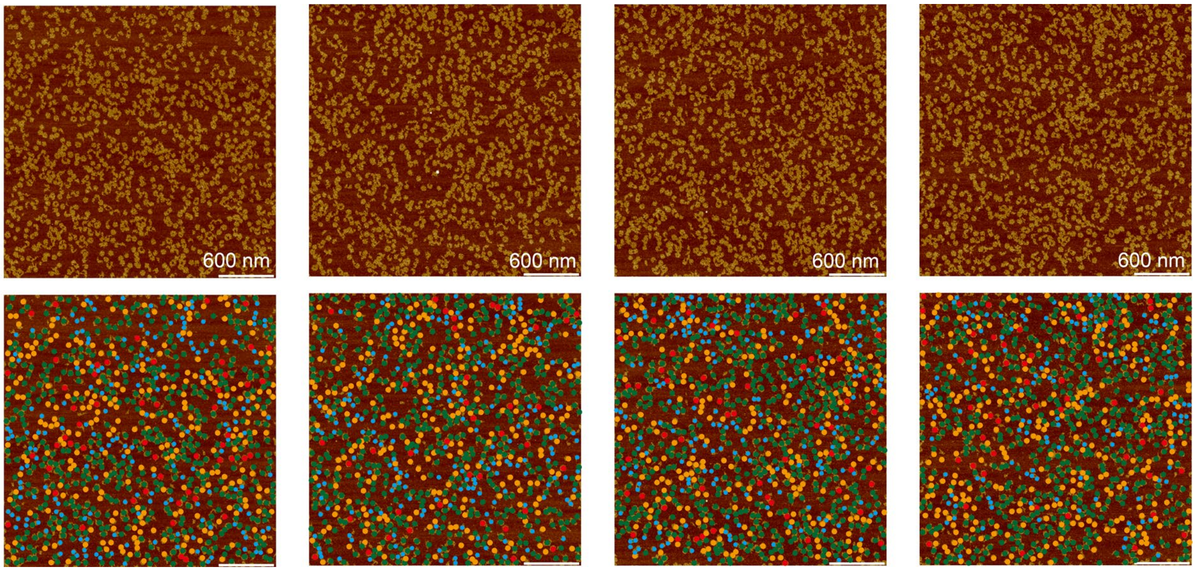

Supplementary Figure 94 | The estimated assembly yield of pair 1 path 9 (N = 5). Four of 3 μm ×3 μm AFM images are used for yield counting, with 4014 total counted nanorings (blue 694, yellow 1007, red 195, green 2118). Source data are provided as a Source Data file.

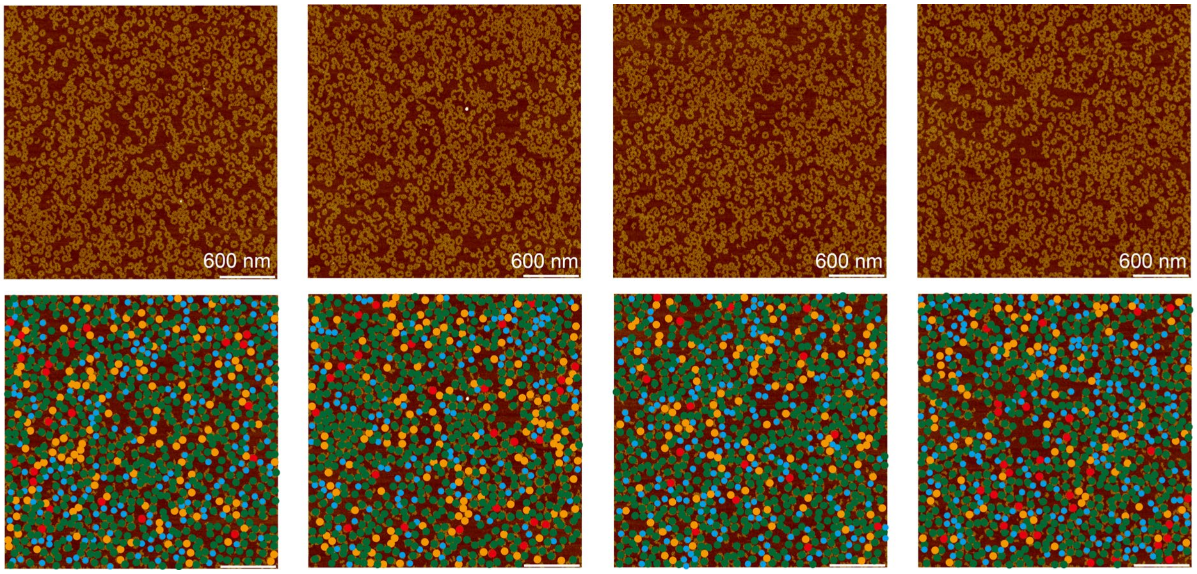

Supplementary Figure 95 | The estimated assembly yield of pair 3 path 10 (N = 9). Four of 3 μm ×3 μm AFM images are used for yield counting, with 3597 total counted nanorings (blue 747, yellow 469, red 98, green 2283). Source data are provided as a Source Data file.

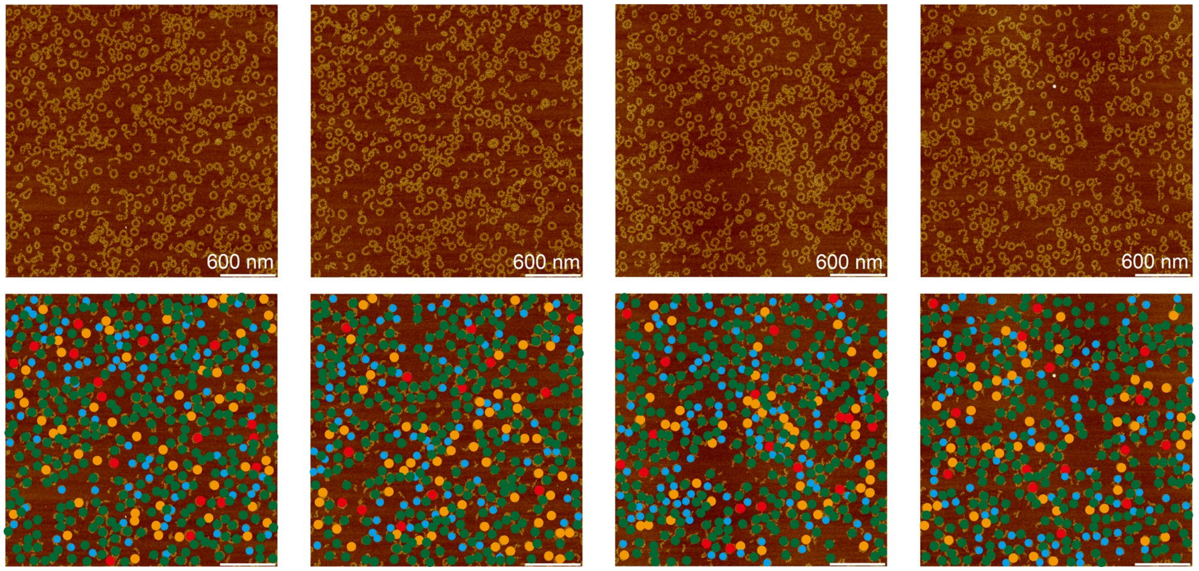

Supplementary Figure 96 | The estimated assembly yield of pair 3 path 11 (*N* = 9). Four of 3 μm ×3 μm AFM images are used for yield counting, with 1764 total counted nanorings (blue 352, yellow 214, red 65, green 1133). Source data are provided as a Source Data file.

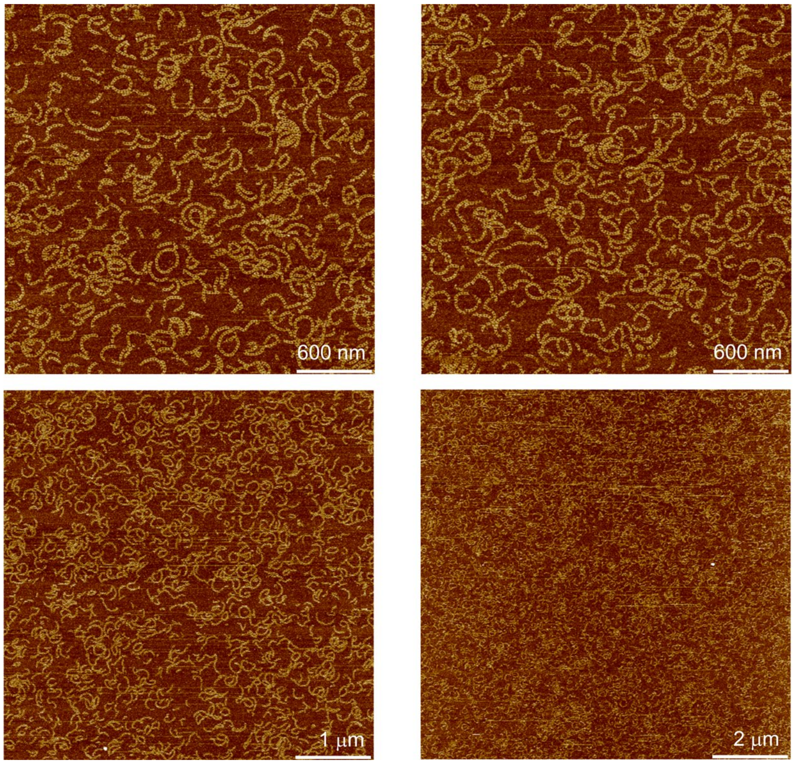

Supplementary Figure 97 | The AFM images of pair 3 path 12 (*N* = 38). The assembly yield of it is not estimated due to the low yield. Source data are provided as a Source Data file.

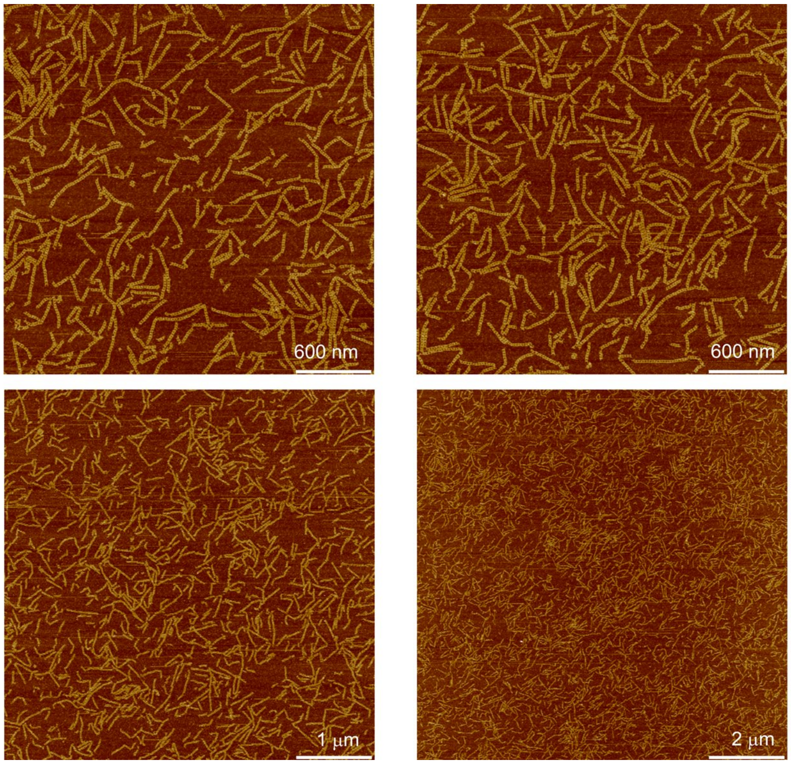

Supplementary Figure 98 | The AFM images of pair 4 ladder. The monomer tile is TC-Z-6-4-6. Source data are provided as a Source Data file.

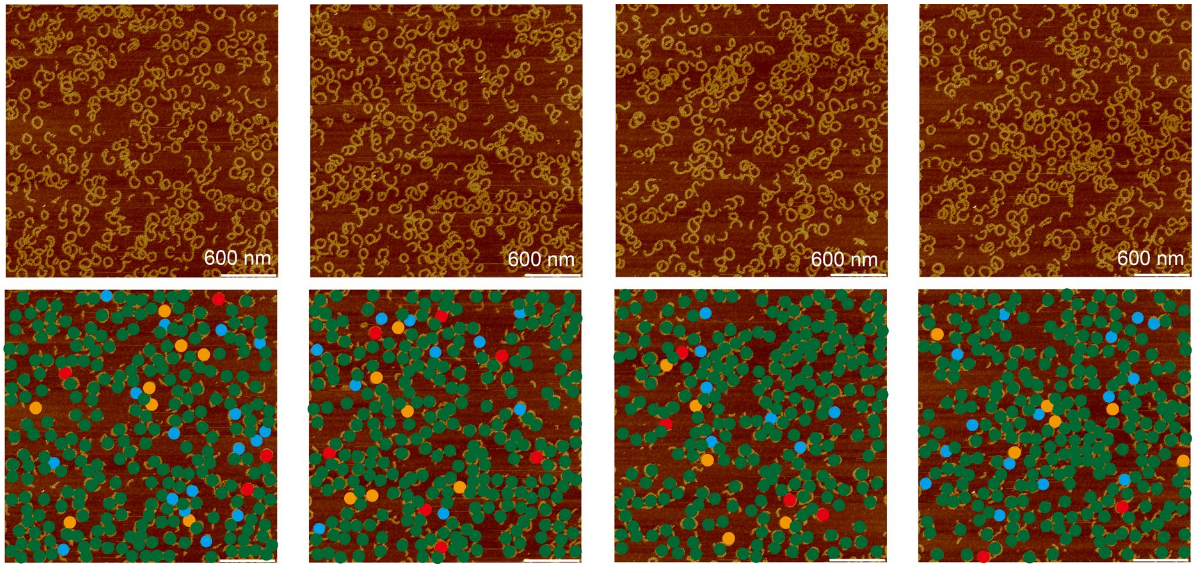

Supplementary Figure 99 | The estimated assembly yield of pair 4 ring 6* (N = 14). The monomer tile is TC-Z-8-4-6. Four of 3 μm ×3 μm AFM images are used for yield counting, with 1064 total counted nanorings (blue 49, yellow 25, red 17, green 973). Source data are provided as a Source Data file.

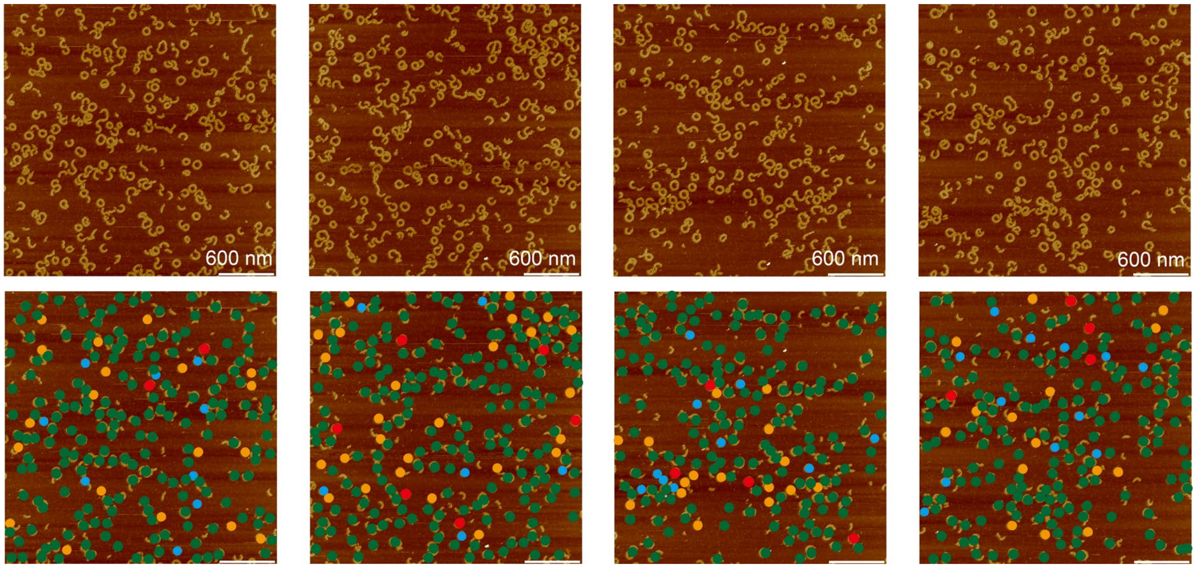

Supplementary Figure 100 | The estimated assembly yield of pair 4 ring 7* (*N* = 14). The monomer tile is TC-Z-6-4-4. Four of 3 μm ×3 μm AFM images are used for yield counting, with 826 total counted nanorings (blue 37, yellow 76, red 12, green 701). Source data are provided as a Source Data file.

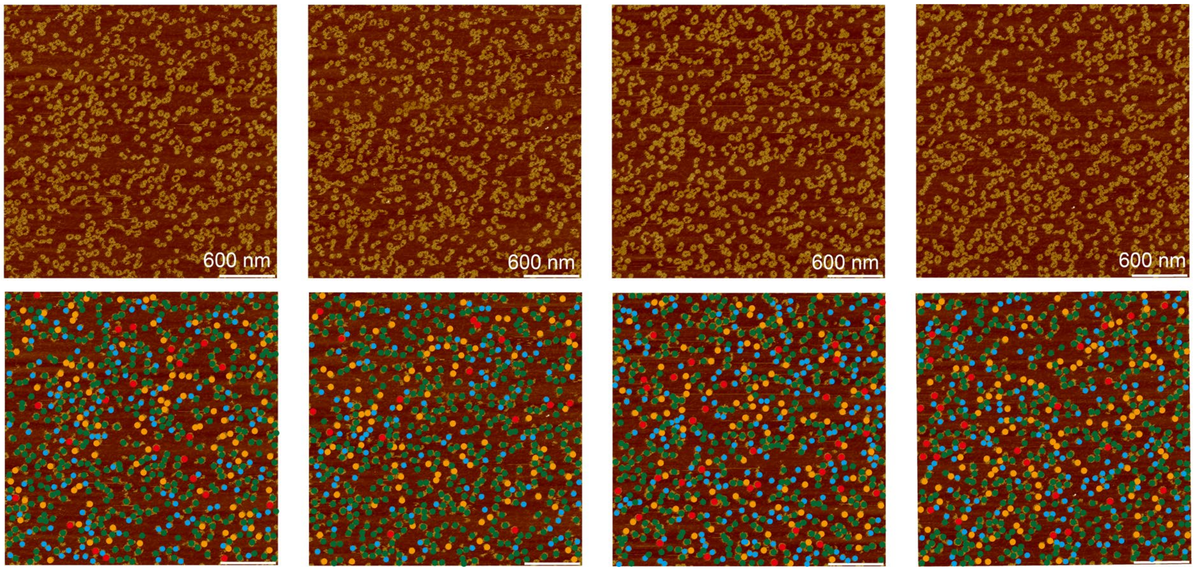

Supplementary Figure 101 | The estimated assembly yield of pair 4 ring 8* (*N* = 7). The monomer tile is TC-Z-8-4-4. Four of 3 μm ×3 μm AFM images are used for yield counting, with 2788 total counted nanorings (blue 576, yellow 414, red 105, green 1693). Source data are provided as a Source Data file.

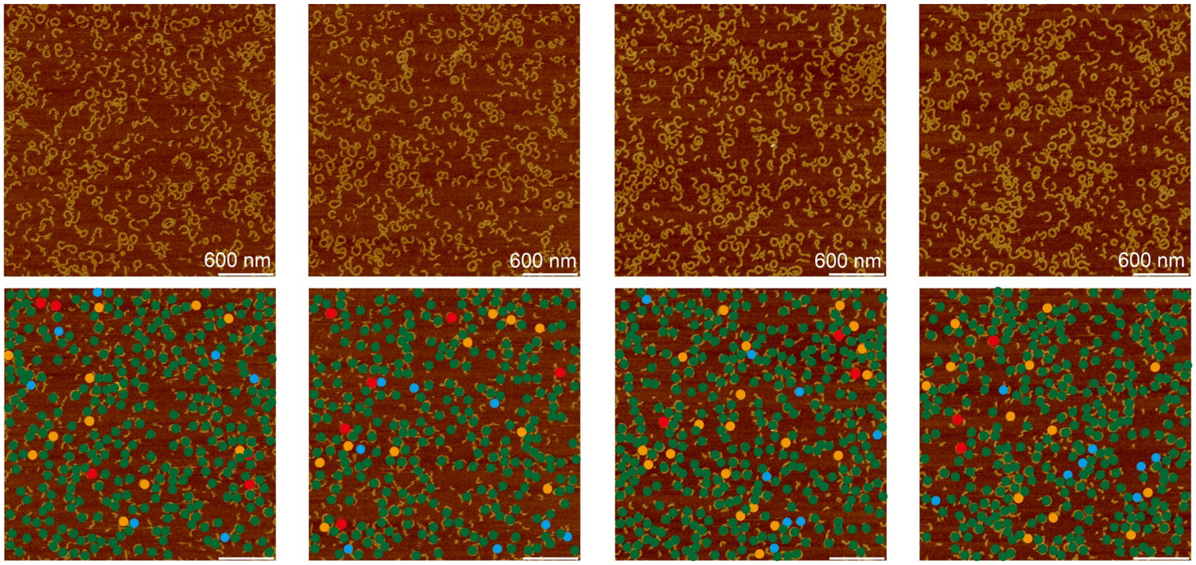

Supplementary Figure 102 | The estimated assembly yield of pair 4 path 1 (N = 14). Four of 3 μm ×3 μm AFM images are used for yield counting, with 1309 total counted nanorings (blue 31, yellow 56, red 17, green 1205). Source data are provided as a Source Data file.

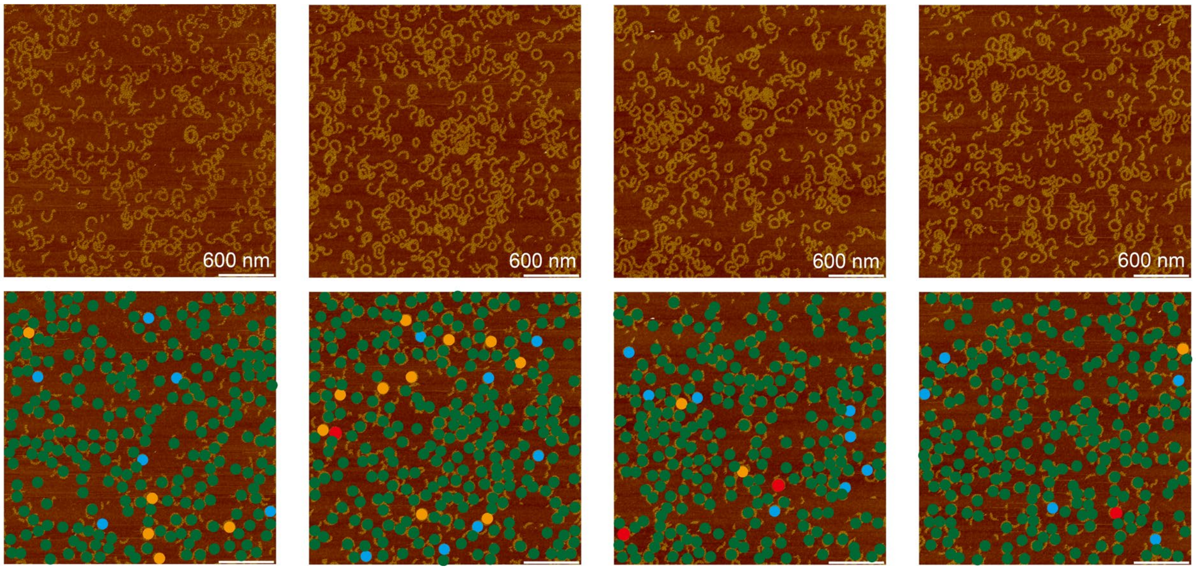

Supplementary Figure 103 | The estimated assembly yield of pair 4 path 2 (N = 14). Four of 3 μm ×3 μm AFM images are used for yield counting, with 1048 total counted nanorings (blue 26, yellow 18, red 4, green 1000). Source data are provided as a Source Data file.

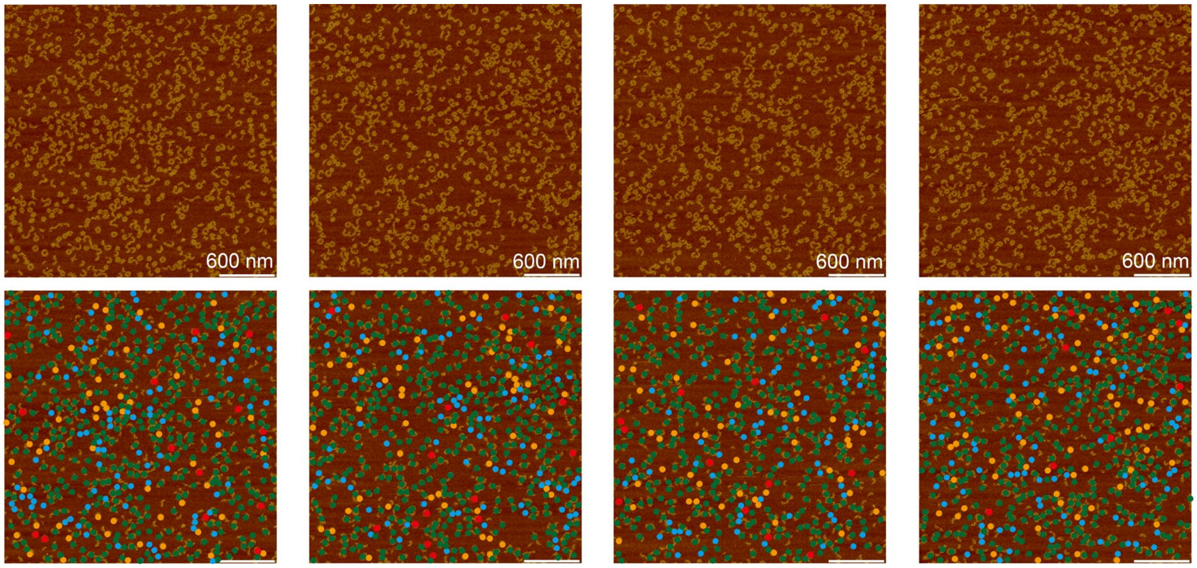

Supplementary Figure 104 | The estimated assembly yield of pair 4 path 3 (N = 7). Four of 3 μm ×3 μm AFM images are used for yield counting, with 2339 total counted nanorings (blue 358, yellow 269, red 50, green 1662). Source data are provided as a Source Data file.

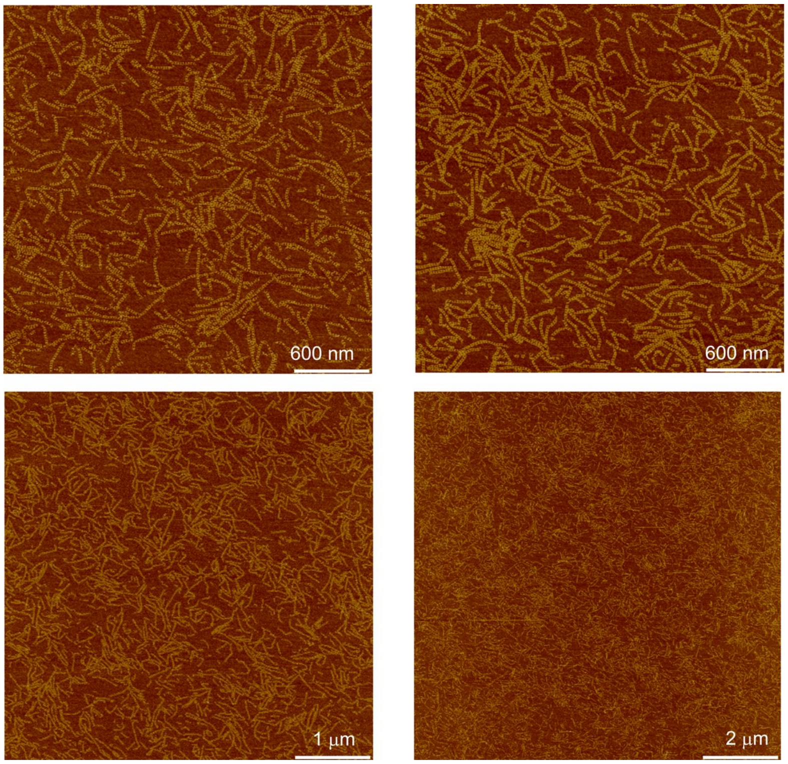

Supplementary Figure 105 | The AFM images of pair4 path 4 (ladder). Source data are provided as a Source Data file.

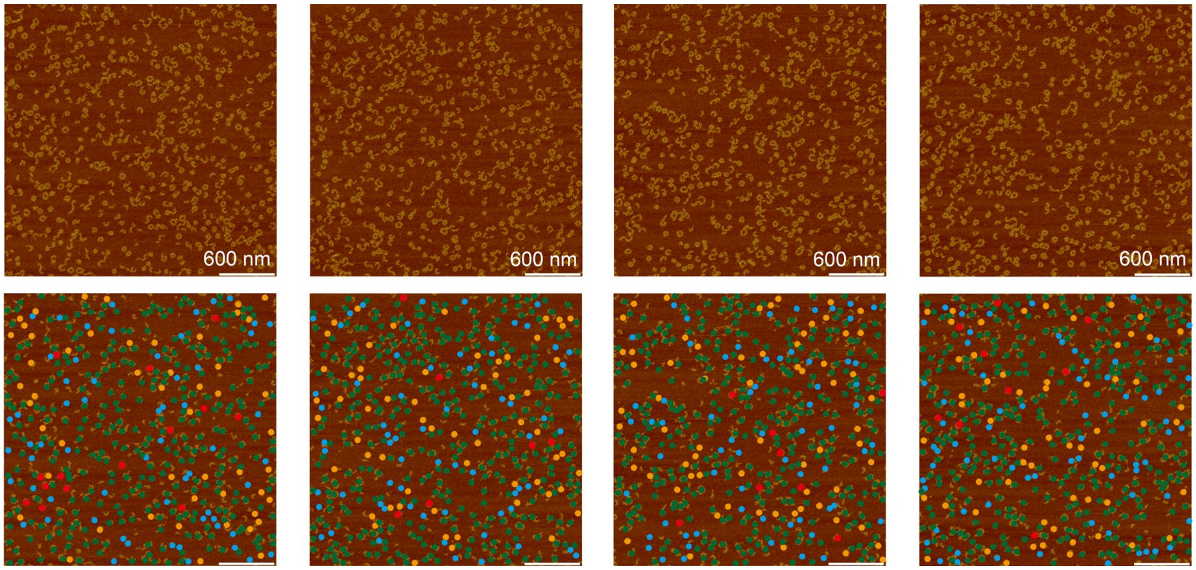

Supplementary Figure 106 | The estimated assembly yield of pair 4 path 5 (N = 14). Four of 3 μm ×3 μm AFM images are used for yield counting, with 1872 total counted nanorings (blue 269, yellow 240, red 38, green 1325). Source data are provided as a Source Data file.

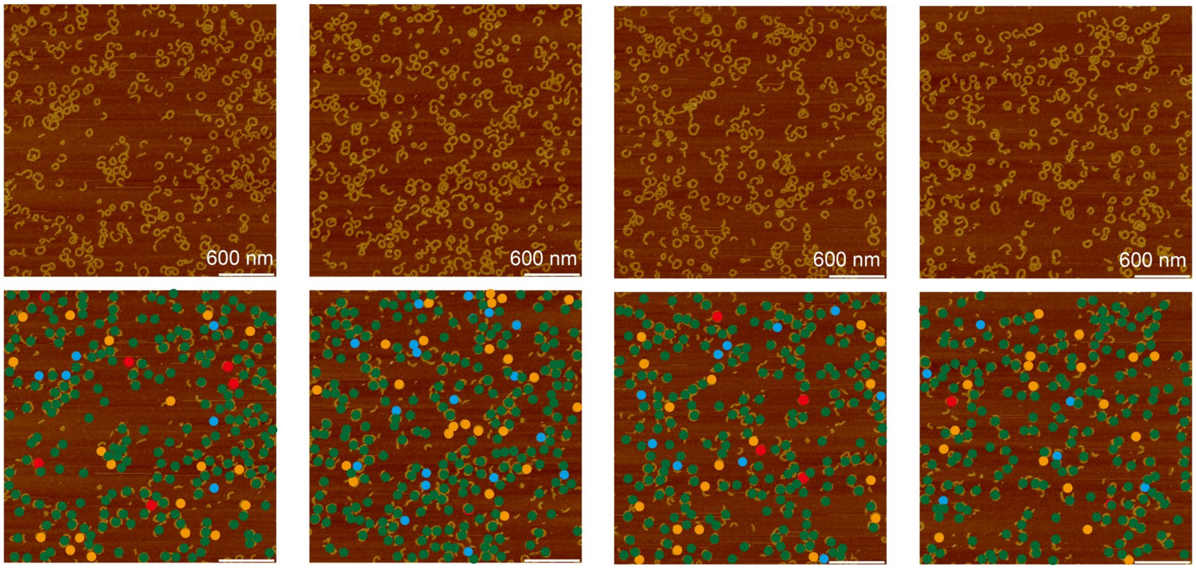

Supplementary Figure 107 | The estimated assembly yield of pair 4 path 6 (N = 14). Four of 3 μm ×3 μm AFM images are used for yield counting, with 971 total counted nanorings (blue 42, yellow 79, red 15, green 835). Source data are provided as a Source Data file.

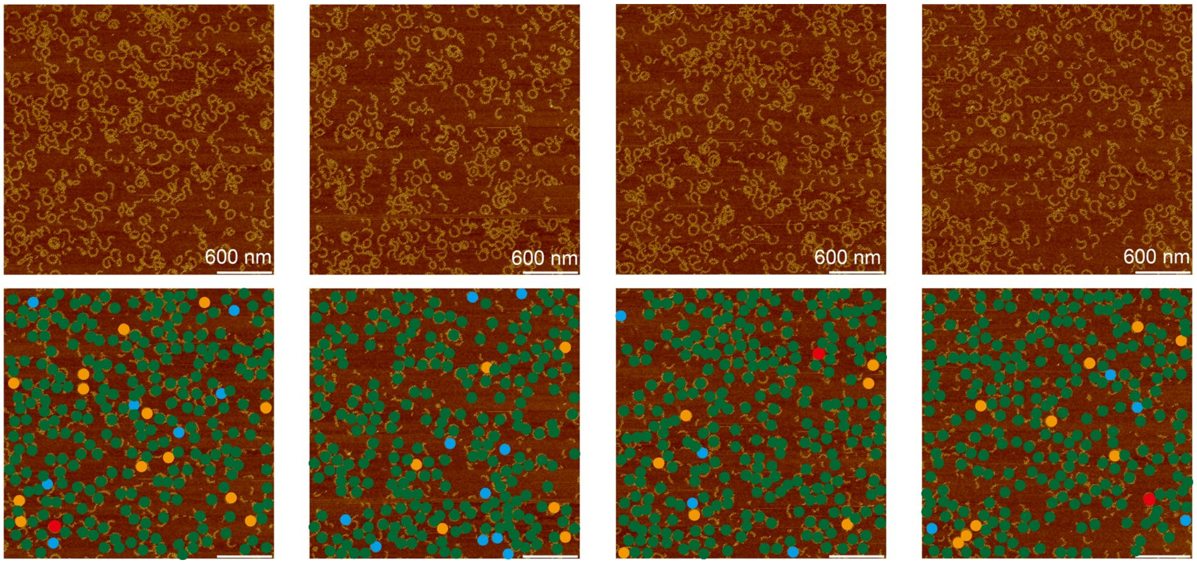

Supplementary Figure 108 | The estimated assembly yield of pair 4 path 7 (N = 14). Four of 3 μm ×3 μm AFM images are used for yield counting, with 1038 total counted nanorings (blue 25, yellow 35, red 3, green 975). Source data are provided as a Source Data file.

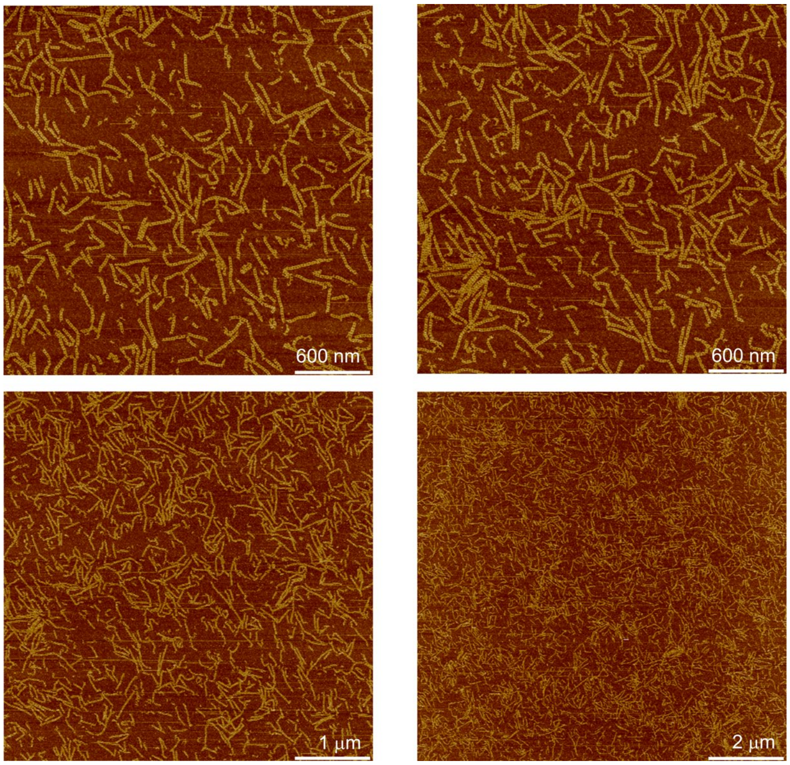

Supplementary Figure 109 | The AFM images of pair 4 path 8 (ladder). Source data are provided as a Source Data file.

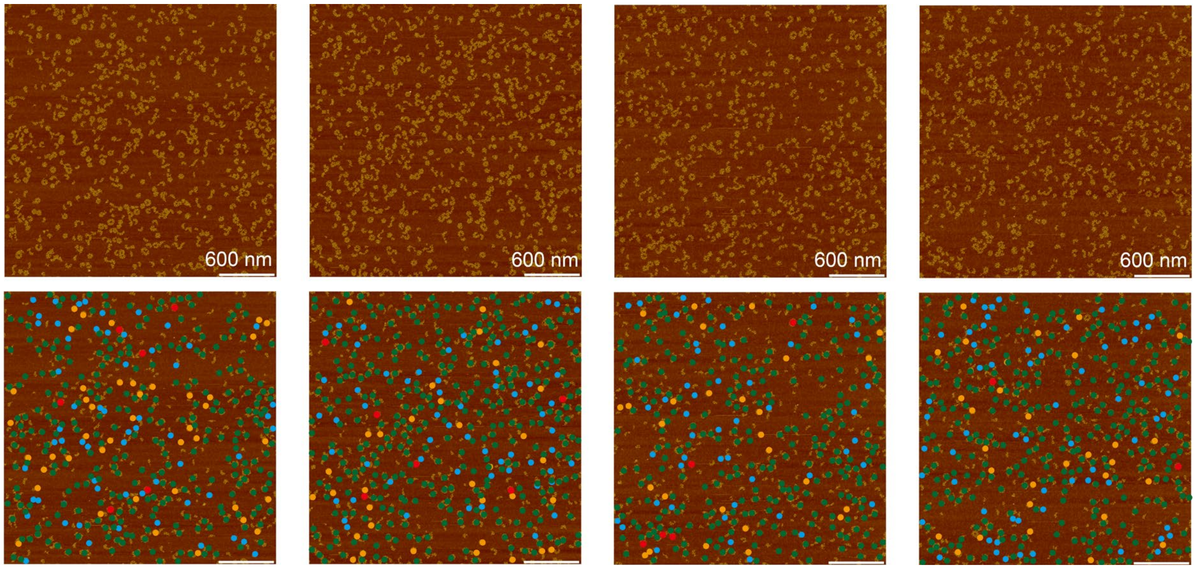

Supplementary Figure 110 | The estimated assembly yield of pair 4 path 9 (N = 14). Four of 3 μm ×3 μm AFM images are used for yield counting, with 1575 total counted nanorings (blue 233, yellow 134, red 19, green 1189). Source data are provided as a Source Data file.

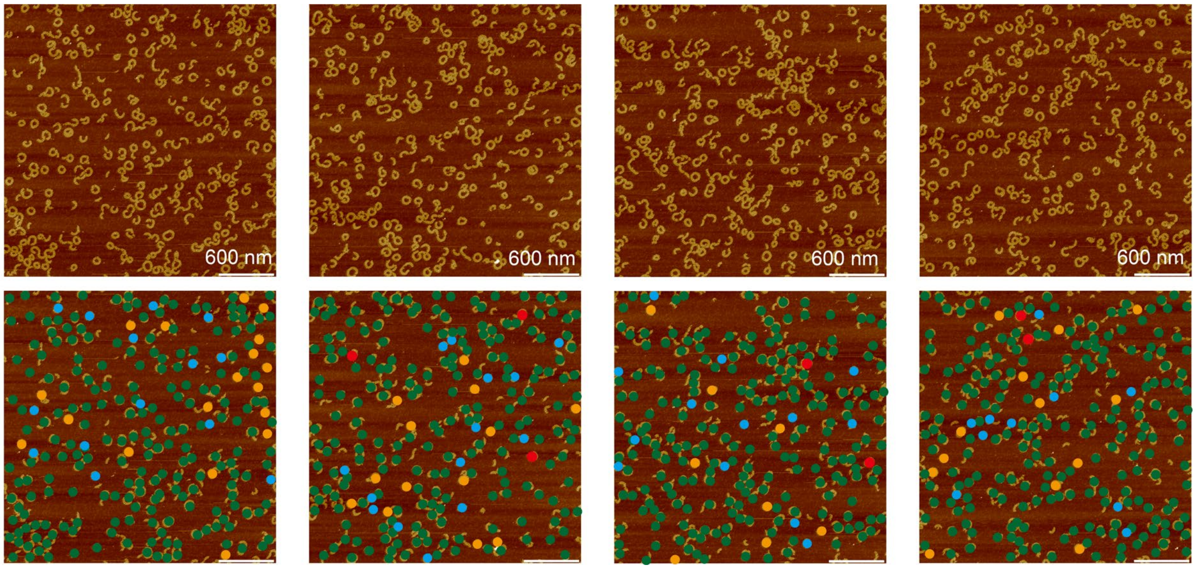

Supplementary Figure111 | The estimated assembly yield of pair 4 path 10 (N = 14). Four of 3 μm ×3 μm AFM images are used for yield counting, with 940 total counted nanorings (blue 51, yellow 52, red 7, green 830). Source data are provided as a Source Data file.

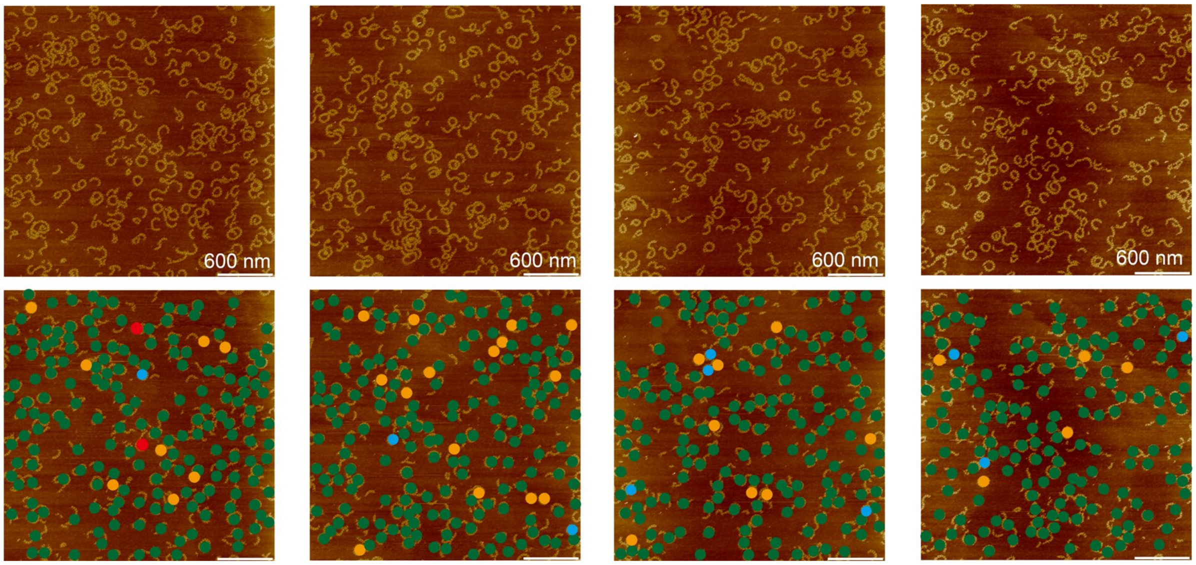

Supplementary Figure 112 | The estimated assembly yield of pair 4 path 11 (N = 14). Four of 3 μm ×3 μm AFM images are used for yield counting, with 723 total counted nanorings (blue 10, yellow 36, red 2, green 675). Source data are provided as a Source Data file.

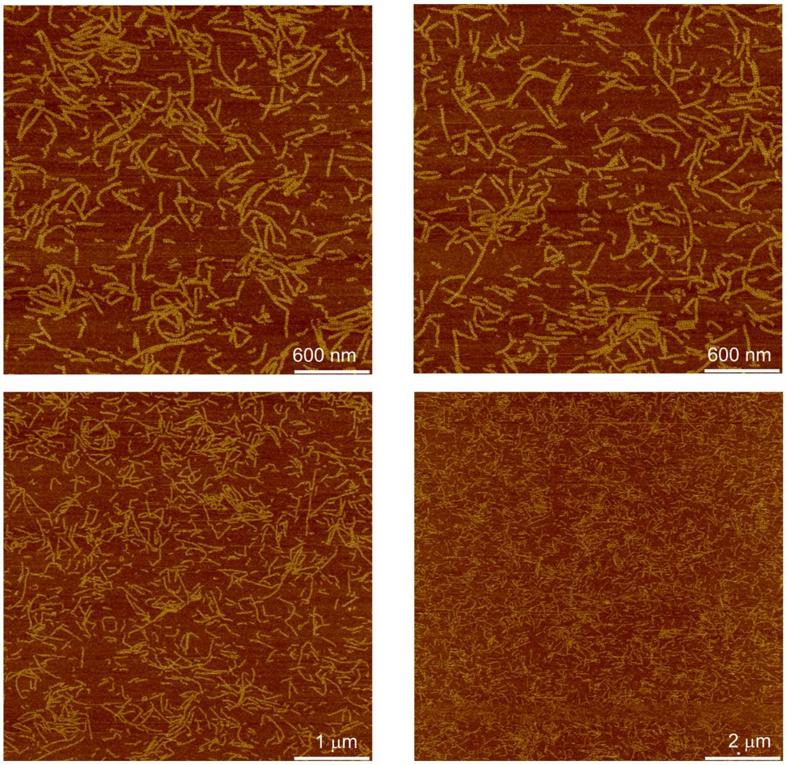

Supplementary Figure 113 | The AFM images of pair 4 path 12 (ladder). Source data are provided as a Source Data file.

Section 3   Supplementary notes

DNA Sequences of each design. The sticky ends are labeled as blue.

TC-1-1

1: CTAGTTGTGTGAGTCCACCGGATGAGATTCCTGAGTGAACT

2: CTCATCAGTTCACTCACCTACACT

3: GACAGTGTAGGACTCAATACT

4: GTCAGTATTGAGTGGAATCGGTGGACTCACACAA

TC-1-2

1: CTAGTTGGGTCATCCCTCCGGAGGTGATTCCTGAATGAATTA

2: GCGCTAATTCATTCACCTACACTGATTGCA

3: ATCAGTGTAGGACTCAATAGT

4: CACCTCACTATTGAGTGGAATCGGAGGGATGACCCAA

TC-2-1

1: CTAGTTGTGTGAGTCGGTACCAAAGCTTCA

2: CTCATCAGTTCACTCACCTACACT

3: GACAGTGTAGGACTCAATACT

4: GTCAGTATTGAGTGGAATCGGTGGACTCACACAA

5: CACCGGATGAGATTCCTGAGTGAACTATGAAGCTTTGGTACC

TC-2-2

1: CTAGTTGTGTCATCCGGTACCAAAGCTTCA

2: GCGCTAATTCATTCACCTACACTGATTGCA

3: ATCAGTGTAGGACTCAATAGT

4: CTCCACACTATTGAGTGGAATCGGAGGGATGACACAA

5: CTCCGGTGGAGATTCCTGAATGAATTATGAAGCTTTGGTACC

TC-3-1

1: ATCCGCCACTCTGATGATAAACACATGCACACTACACCCGTCCGCGGAAGGAGA

2: TGGGAAGCGCTACTTGGTTCCACGGGTGTAGTTCCCATGTGCATGTGTTTATCATCAGAGTG

3: TGAGCGTTTGTGAGCAGCAGGAACCAAGTGCGGATAGCGC

4: CGCTCATCTCCTTCCGCGGTGCTGCTCACAAA

TC-3-2

1: ATCCGCTGTGCTCACTCTGATGATAAACACACGCACACTACACCCGTCCGCGGAAGGAGA

2: TGGGAAGCGCTACTTGGTTCCACGGGTGTAGTTCCCATGTGCGTGTGTTTATCATCAGAGTGAGCACA

3: TGAGCGTTTGTGAGCAGCAGGAACCAAGTGCGGATAGCGC

4: CGCTCATCTCCTTCCGCGGTGCTGCTCACAAA

TC-3-3

1: ATCCGCTGTGCTCACTCTGATGATAAACACACGCACACTACACCCGTCCGCGGAAGGAGA

2: TGGGAAGCGCTACTTGGTTCCACGGGTGTAGTTCCCATGTGCGTGTGTTTATCATCAGAGTGAGCACA

3: TCGTCTCCTTCCGCGGTGCTGCTCACAAACGC

4: CGAGCGTTTGTGAGCAGCAGGAACCAAGTGCGGATAGCGC

TC-4-1

1: AACACCACTGAAGGCC

2: TTCAGTCCACAGCTACGAGAAGAATCCTGAGTGTCTA

3: CTTCTCTAGACCAATCCATCAGGTGTTGTAC

4: ACTGGTGGATTGTAGCTGTGGTGATGGATTG

5: AAGAGACAAGGACCAGTTAAG

6: ACTCACCTTGTCTCTTCTTA

TC-4-2

1: AACCCACTAGTAGGCC

2: TACTAGCATGGGCAGCGAGGTGATTCCTGTAACTATAT

3: CGCGATATAGGCCTCATTACTGGGTTGATC

4: CACCTCTTCTAGTTGTGGAATGCTGCCCATGGTAATGAGGC

5: AAGAGACAAGGACAACTAGAA

6: TTACACCTTGTCTCTTCATG

TC-5-1

1: CTTGTCTCAACCACACTCGG

2: GACATGCTAGCTCTGGTGGAATCGCTTGCAGTATCTGATGCCCACGAT

3: GAAGGGTCTGTGGAGGAGGTGAGGAACAGTGT

4: CAAGCCGAGTGTCCAGAGCTCCCTTCAGCATGTCATCGTCCTCCTCCACAGA

5: TGCAAGACACTGTTCCTCAGGGCATCAGATACCGATTCCAGGTTGAGA

TC-5-2

1: CTTGTCTCAACCACACTCGG

2: GACATGCTAGCTCTGGTGGAATCGCTTGCAGTATCTGATGCCCACGAT

3: GAAGGGTCTCTCAGGGCATCAGATACCGATTCCAGGTTGAGA

4: CAAGCCGAGTGTCCAGAGCTCCCTTCAGCATGTCATCGTCCTCCTCCACAGA

5: TGCAAGGTGGAGGTGAGAGA

TC-5-3

1: GGCTTGTCTCAACCACACTC

2: GACATGCTAGCTCTGGTGGAATCGCTTGCAGTATCTGATGCCCACGAT

3: CAAGCCGAGTGTCCAGAGCTCCCTTCAGCATGTCATCGTCCTCCAC

4: GAAGGGTCTCTCAGGGCATCAGATACCGATTCCAGGTTGAGA

5: TGCAAGGTGGAGGTGAGAGA

TC-6-1

1: TAGCGATGTTCCTAGGTCGACACTCAAGCGTTAC

2: ACTCAGCAGGCACACTAGTCCACTCC

3: CTCTCCACTAGTGTGCCACAAA

4: CATCGAGGTGCTGAGTGTAAC

5: GCTTGAGTGTCGGAGTGGTGATGTGGACCTAGGAACATCGCTACCTAGG

6: TTGACCCGTCACACATCAGGAGAGTTTGTCCTCGATGGCACT

7: TGACGGGTCAAAGTGC

TC-6-2

1: TCACGGATGCTGGTGCTGAGTGTAACC

2: CGTGAAGTGTCGGAGTGGTGATGTGCAGAGACCTTTCAGAGTC

3: CATTGCACATCAGGAGAGTTTGTCCAGCATC

4: CTCTCCACTAGTGTGCCACAAA

5: GGATCCGACTCTGAAAGGTCTCTGGACACT

6: CAATGGGTTACACTCAGCAGGCACACTAGTCCACTCC

TC-7-1

1: GGATCCGTTGAACACACCTTTGCTGAGACGTC

2: TACTGTCTGGACCTGAACCGATCACCTACCTGCTACCTGAGGACCTGTGTTCAAC

3: ATCATAAGTGCTCTCACTGTTGTGGTCCTCAGGTAGTCAGGTCCAG

4: TCAGCAAAGGACAACAGTGAGAGACAGTAAGCTCCCAGGACAGT

5: ACTCTGGTCTAAGGTGGGAGCT

6: TGAGCACTGTCCACAACGTCT

7: CAATGAGACGTTGTCCTTAGACCAGAGTCACTTATGATCAGGTAGGACCAGCT

8: CATTGAGCTGGTGGGTGTAAC

9: GCTCAGTTACACCCTGATCGGT

TC-7-2

1: GGATCCGTTGAACACACCTTTGCTGAGACGTC

2: TACTGTCTGGACCTGAACCGATCACCTACCTGCTACCTGAGGACCTGTGTTCAAC

3: ATCATAAGTGCTCTCACTGTTGTGGTCCTCAGGTAGTCAGGTCCAG

4: TCAGCAAAGGACAACAGTGAGAGACAGTAAGCTCCCAGGACAGT

5: ACTCTGGTCTAAGGTGGGAGCT

6: TGAGCACTGTCCACAACGTCT

7: CAATGAGACGTTGTCCTTAGACCAGAGTCACTTATGATCAGGTAGGACCAAGCAGAACGA

8: CATTGTGAGTCAGATCCTGATCGGT

9: GCTCATCGTTCTGCTTGGTGGATCTGACTCA

TC-8-1

1: CACCTCACTATTGAGTGGAATCGGAGGGATGACACAA

2: GCGCTAATTCATTCACCTACACTGATTGCA

3: ATCAGTGTAGGACTCAATAGT

4: AAAATTGTGTCATCCCTCCGGGGAGGATTCCTGAATGAATTA

5: TACTAGCATGGGCAGCGAGGTGATTCCTGTAACTATAT

6: CCTCCCTTCTAGTTGTGGAATGCTGCCCATGCTAGTAAAAA

7: CGCGATATAGTTACACCTTGTCTCTTCATG

8: AAGAGACAAGGACAACTAGAA

TC-8-2

1: CACCTCACTATTGAGTGGAATCGGAGGGATGACACAA

2: GCGCTAATTCATTCACCTACACTGATTGCA

3: ATCAGTGTAGGACTCAATAGT

4: CTAGTTGTGTCATCCCTCCGGGGAGGATTCCTGAATGAATTA

5: TACTAGCATGGGCAGCGAGGTGATTCCTGTAACTATAT

6: CCTCCCTTCTAGTTGTGGAATGCTGCCCATGCTAGTAAAAA

7: CGCGATATAGTTACACCTTGTCTCTTCATG

8: AAGAGACAAGGACAACTAGAA

TC- 8-3

1: CACCTCACTATTGAGTGGAATCGGAGGGATGACACAA

2: GCGCTAATTCATTCACCTACACTGATTGCA

3: ATCAGTGTAGGACTCAATAGT

4: CTAGTTGTGTCATCCCTCCGGGGAGGATTCCTGAATGAATTA

5: TACTAGCATGGGCAGCGAGGTGATTCCTGTAACTATAT

6: CCTCCCTTCTAGTTGTGGAATGCTGCCCATGCTAGTAGGCC

7: CGCGATATAGTTACACCTTGTCTCTTCATG

8: AAGAGACAAGGACAACTAGAA

TC-8-4

1: TACTAGCATGGGCAGCGAGGTGATTCCTGTAACTATACA

2: GCTGTATAGTTACACCTTGTCTCTGAAT

3: TCAGAGACAAGGACAACTAGAA

4: CCTCCCTTCTAGTTGTGGAATGCTGCCCATGCTAGTATCGA

5: CACCTCACTATTGAGTGGAATCGGAGGGATGACACAA

6: CTAGTTGTGTCATCCCTCCGGGGAGGATTCCTGAATGAATGAA

7: CGTTCATTCATTCACCTACACTGACTTA

8: AGTCAGTGTAGGACTCAATAGT

TC-9-1

1: GCAGCGAGGTGATTCCTGTAACTATATAGAAGTGAGTAGTAA

2: TACTAGCATGGTTACTACTCACTTCT

3: CCTCCCTTCTAGTTGTGGAATGCTGCCCATGCTAGTAAAAA

4: CTCCGGGGAGGATTCCTGAATGAATTATGAAGCTTTGGTACC

5: AAAATTGTGTCATCCGGTACCAAAGCTTCA

6: CACCTCACTATTGAGTGGAATCGGAGGGATGACACAA

7: ATCAGTGTAGGACTCAATAGT

8: GCGCTAATTCATTCACCTACACTGATTGCA

9: AAGAGACAAGGACAACTAGAA

10: CGCGATATAGTTACACCTTGTCTCTTCATG

TC- 9-2

1: CACCTCACTATTGAGTGGAATCGGAGGGATGACACAA

2: ATCAGTGTAGGACTCAATAGT

3: GCAGCGAGGTGATTCCTGTAACTATATAGAAGTGAGTAGTAA

4: CTCCGGGGAGGATTCCTGAATGAATTATGAAGCTTTGGTACC

5: CTAGTTGTGTCATCCGGTACCAAAGCTTCA

6: CCTCCCTTCTAGTTGTGGAATGCTGCCCATGCTAGTAAAAA

7: TACTAGCATGGTTACTACTCACTTCT

8: GCGCTAATTCATTCACCTACACTGATTGCA

9: AAGAGACAAGGACAACTAGAA

10: CGCGATATAGTTACACCTTGTCTCTTCATG

TC-9-3

1: CACCTCACTATTGAGTGGAATCGGAGGGATGACACAA

2: ATCAGTGTAGGACTCAATAGT

3: GCAGCGAGGTGATTCCTGTAACTATATAGAAGTGAGTAGTAA

4: CTCCGGGGAGGATTCCTGAATGAATTATGAAGCTTTGGTACC

5: CTAGTTGTGTCATCCGGTACCAAAGCTTCA

6: CCTCCCTTCTAGTTGTGGAATGCTGCCCATGCTAGTAGGCC

7: TACTAGCATGGTTACTACTCACTTCT

8: GCGCTAATTCATTCACCTACACTGATTGCA

9: AAGAGACAAGGACAACTAGAA

10: CGCGATATAGTTACACCTTGTCTCTTCATG

Defined ring without SDR

1:
ATCCGCAGTGTCGGCATCTGAAGAAGTCTGTGTCGAGTGGCACCGTCCACCTTAAAGGAG
CTTCCAGCAGTCACC

AAC

2:
ACCGCACCCCAGCTAAGCACCAGCTCCTTTATGCGGTAGGTGGACGGTGCCACTCGACAC
AGACTTCTTCAGAT

GCCGACACT

3: ATACAACTACTCGACAGGTGCTTAGCGCGGATTGGGG

4:
CAGCTGCGTCCTTAATCTCGAGAGTAATGGGGTTCTGCCAGCCACATATGTGGTTGGTGAC
TGCTGGA

5: TGTCGAGTAGTTGTATCGAAGTATGGGCTTTTGTAACGTAAACTCCGCAGCTGTGACC

6: GTTACAAAAGCCCATACTTCGCACATATGTGGCTGGCAGAAC

7: GGCCTGGGTCACGGAGTTTACCCCATTACTCCAGGCCTCGAGATTAAGGACG

Invader and setting strands for 4-state nanorings transformation.

Invader strand 1: GCTATTGGTGATGTCCTGTTTTTACGGACATTTCAACGTTAAATCCAAGG

Invader strand 2: ACAGACACAGAGACACTTTTTTGGTCTCAACAAGCATCATCTCTAGGTT

Invader strand 3: GCTATTGGTGATCAACGTTAAAGTGATGG

Invader strand 4: ACAGACACACAAGCATCATCTACAGTTG

setting strand 1: CCATCACTTTAACGTTGATCACCAATAGC

setting strand 2: CAACTGTAGATGATGCTTGTG TGTCTGT

setting strand 3: CCTTGGATTTAACGTTGAAATGTCCGTAAAAACAGGACATCACCAATAGC

setting strand 4: AACCTAGAGATGATGCTTGTTGAGACCAAAAAAGTGTCTCTGTGTCTGT

TC-C-9-3.5-4

1:
CAGCTCGCTATTGGTGATGTCCTGTTTTTACGGACATTTCAACGTTAAGATCAGGAAGGTCA
GTCAGGTCTCTCAG

CGTCAGGTCCGTGC

2: TTCTCATGGTGCATCTGATTCAGTGAGCTGCTACC

3:
ATCCGCACAGACACAGAGACACTTTTTTGGTCTCAACAAGCATCATCCACCTTAAACGACGT
CC

4: GGCGTCGGTAGACTGAATCAGATGCAGGAGACAAGCAGGAGCTTAGCGCGGATTGGGG

5: TGCTTGTCTCCACCTCTCGCGG

6:
GCGAGAGGTCCATGAGAAGCACGGGACGCCACCTGACGCTGAGAGACCTGACTGACCTT
CCTGATC

7: ACCGCACCCCAGCTAAGCTCCACGTCGTTTATGCGGTAGGTG

8: CCTTGGATTTAACGTTGAAATGTCCGTAAAAACAGGACATCACCAATAGC

9: CAACTGTAGATGATGCTTGTGTGTCTGT

TC-C-9-3.5-6

1:
CAGCTCGCTATTGGTGATGTCCTGTTTTTACGGACATTTCAACGTTAAGATCAGGAAGGTCA
GTCAGGTCTCTCAGCGTCAGGTCCGTGC

2: TTCTCATGGTGCATCTGATTCAGTGAGCTGCTACC

3:
ATCCGCACAGACACAGAGACACTTTTTTGGTCTCAACAAGCATCATCCACCTTAAACGACGT
CC

4: GGCGTCGGTAGACTGAATCAGATGCAGGAGACAAGCAGGAGCTTAGCGCGGATTGGGG

5: TGCTTGTCTCCACCTCTCGCGG

6:
GCGAGAGGTCCATGAGAAGCACGGGACGCCACCTGACGCTGAGAGACCTGACTGACCTT
CCTGATC

7: ACCGCACCCCAGCTAAGCTCCACGTCGTTTATGCGGTAGGTG

8: CCTTGGATTTAACGTTGAAATGTCCGTAAAAACAGGACATCACCAATAGC

9: AACCTAGAGATGATGCTTGTTGAGACCAAAAAAGTGTCTCTGTGTCTGT

TC-C-7-3.5-4

1:
CAGCTCGCTATTGGTGATGTCCTGTTTTTACGGACATTTCAACGTTAAGATCAGGAAGGTCA
GTCAGGTCTCTCAG

CGTCAGGTCCGTGC

2: TTCTCATGGTGCATCTGATTCAGTGAGCTGCTACC

3:
ATCCGCACAGACACAGAGACACTTTTTTGGTCTCAACAAGCATCATCCACCTTAAACGACGT
CC

4: GGCGTCGGTAGACTGAATCAGATGCAGGAGACAAGCAGGAGCTTAGCGCGGATTGGGG

5: TGCTTGTCTCCACCTCTCGCGG

6:
GCGAGAGGTCCATGAGAAGCACGGGACGCCACCTGACGCTGAGAGACCTGACTGACCTT
CCTGATC

7: ACCGCACCCCAGCTAAGCTCCACGTCGTTTATGCGGTAGGTG

8: CCATCACTTTAACGTTGATCACCAATAGC

9: CAACTGTAGATGATGCTTGTGTGTCTGT

TC-C-7-3.5-6

1:
CAGCTCGCTATTGGTGATGTCCTGTTTTTACGGACATTTCAACGTTAAGATCAGGAAGGTCA
GTCAGGTCTCTCAGCGTCAGGTCCGTGC

2: TTCTCATGGTGCATCTGATTCAGTGAGCTGCTACC

3:
ATCCGCACAGACACAGAGACACTTTTTTGGTCTCAACAAGCATCATCCACCTTAAACGACGT
CC

4: GGCGTCGGTAGACTGAATCAGATGCAGGAGACAAGCAGGAGCTTAGCGCGGATTGGGG

5: TGCTTGTCTCCACCTCTCGCGG

6:
GCGAGAGGTCCATGAGAAGCACGGGACGCCACCTGACGCTGAGAGACCTGACTGACCTT
CCTGATC

7: ACCGCACCCCAGCTAAGCTCCACGTCGTTTATGCGGTAGGTG

8: CCATCACTTTAACGTTGATCACCAATAGC

9: AACCTAGAGATGATGCTTGTTGAGACCAAAAAAGTGTCTCTGTGTCTGT

TC-C-9-5.5-4

1:
CAGCTCGCTATTGGTGATGTCCTGTTTTTACGGACATTTCAACGTTAAGATCAGGAAGGTCA
GTCAGGTCTCTCAG

CGTCAGGTCCGTGC

2:
ACGATCACTGTCCTTTTCTGGTCCATGAGAAGCACGGGACGCCACCTGACGCTGAGAGAC
CTGACTGACCTTCCT

GATC

3:
GGCGTCGGTAGACTGAATCAGATGCAGGAGACAGACAGGCAGTTTCTACTAGTGCTCAGGA
GCTTAGCGCGGAT

TGGGG

4: TTCTCATGGTGCATCTGATTCAGTGAGCTGCTACC

5: TGTCTGTCTCCACCAGAAAAGG

6: TGAGCACTAGTAGAAACTGCCACAGTGATCGTGACTGCTTGG

7:
ATCCGCACAGACACAGAGACACTTTTTTGGTCTCAACAAGCATCATCCACCTTAAACGACGT
CCAAGCAGTC

8: ACCGCACCCCAGCTAAGCTCCACGTCGTTTATGCGGTAGGTG

9: CCTTGGATTTAACGTTGAAATGTCCGTAAAAACAGGACATCACCAATAGC

10: CAACTGTAGATGATGCTTGTGTGTCTGT

TC-C-9-5.5-6

1:
CAGCTCGCTATTGGTGATGTCCTGTTTTTACGGACATTTCAACGTTAAGATCAGGAAGGTCA
GTCAGGTCTCTCAG

CGTCAGGTCCGTGC

2:
ACGATCACTGTCCTTTTCTGGTCCATGAGAAGCACGGGACGCCACCTGACGCTGAGAGAC
CTGACTGACCTTCC

TGATC

3:
GGCGTCGGTAGACTGAATCAGATGCAGGAGACAGACAGGCAGTTTCTACTAGTGCTCAGGA
GCTTAGCGCGGA

TTGGGG

4: TTCTCATGGTGCATCTGATTCAGTGAGCTGCTACC

5: TGTCTGTCTCCACCAGAAAAGG

6: TGAGCACTAGTAGAAACTGCCACAGTGATCGTGACTGCTTGG

7:
ATCCGCACAGACACAGAGACACTTTTTTGGTCTCAACAAGCATCATCCACCTTAAACGACGT
CCAAGCAGTC

8: ACCGCACCCCAGCTAAGCTCCACGTCGTTTATGCGGTAGGTG

9: CCTTGGATTTAACGTTGAAATGTCCGTAAAAACAGGACATCACCAATAGC

10: AACCTAGAGATGATGCTTGTTGAGACCAAAAAAGTGTCTCTGTGTCTGT

TC-C-7-5.5-4

1:
CAGCTCGCTATTGGTGATGTCCTGTTTTTACGGACATTTCAACGTTAAGATCAGGAAGGTCA
GTCAGGTCTCTCAG

CGTCAGGTCCGTGC

2:
ACGATCACTGTCCTTTTCTGGTCCATGAGAAGCACGGGACGCCACCTGACGCTGAGAGAC
CTGACTGACCTTCC

TGATC

3:
GGCGTCGGTAGACTGAATCAGATGCAGGAGACAGACAGGCAGTTTCTACTAGTGCTCAGGA
GCTTAGCGCGGA

TTGGGG

4: TTCTCATGGTGCATCTGATTCAGTGAGCTGCTACC

5: TGTCTGTCTCCACCAGAAAAGG

6: TGAGCACTAGTAGAAACTGCCACAGTGATCGTGACTGCTTGG

7:
ATCCGCACAGACACAGAGACACTTTTTTGGTCTCAACAAGCATCATCCACCTTAAACGACGT
CCAAGCAGTC

8: ACCGCACCCCAGCTAAGCTCCACGTCGTTTATGCGGTAGGTG

9: CCATCACTTTAACGTTGATCACCAATAGC

10: CAACTGTAGATGATGCTTGTGTGTCTGT

TC-C-7-5.5-6

1:
CAGCTCGCTATTGGTGATGTCCTGTTTTTACGGACATTTCAACGTTAAGATCAGGAAGGTCA
GTCAGGTCTCTCAG

CGTCAGGTCCGTGC

2:
ACGATCACTGTCCTTTTCTGGTCCATGAGAAGCACGGGACGCCACCTGACGCTGAGAGAC
CTGACTGACCTTCC

TGATC

3:
GGCGTCGGTAGACTGAATCAGATGCAGGAGACAGACAGGCAGTTTCTACTAGTGCTCAGGA
GCTTAGCGCGGA

TTGGGG

4: TTCTCATGGTGCATCTGATTCAGTGAGCTGCTACC

5: TGTCTGTCTCCACCAGAAAAGG

6: TGAGCACTAGTAGAAACTGCCACAGTGATCGTGACTGCTTGG

7:
ATCCGCACAGACACAGAGACACTTTTTTGGTCTCAACAAGCATCATCCACCTTAAACGACGT
CCAAGCAGTC

8: ACCGCACCCCAGCTAAGCTCCACGTCGTTTATGCGGTAGGTG

9: CCATCACTTTAACGTTGATCACCAATAGC

10: AACCTAGAGATGATGCTTGTTGAGACCAAAAAAGTGTCTCTGTGTCTGT

TC-Z-9-4-4

1:
CAGCTCGCTATTGGTGATGTCCTGTTTTTACGGACATTTCAACGTTAAGATCAGGAAGGTCA
GTCAGGTCTCTCAG

CGTCAGGTCCGTGC

2:
TGTGTCCTTTTCTGGTCCATGAGAAGCACGGGACGCCACCTGACGCTGAGAGACCTGACT
GACCTTCCTGATC

3: GGCGTCGGTAGACTGAATCAGATGCATGTACAGACTGCTTGG

4: TTCTCATGGTGCATCTGATTCAGTGAGCTGCTACC

5: TGTACAACCAGAAAAGGACACAGGAGCTTAGCGCGGATTGGGG

6:
ATCCGCACAGACACAGAGACACTTTTTTGGTCTCAACAAGCATCATCCACCTTAAACGACGT
CCAAGCAGTC

7: ACCGCACCCCAGCTAAGCTCCACGTCGTTTATGCGGTAGGTG

8: CCTTGGATTTAACGTTGAAATGTCCGTAAAAACAGGACATCACCAATAGC

9: CAACTGTAGATGATGCTTGTGTGTCTGT

TC-Z-9-4-6

1:
CAGCTCGCTATTGGTGATGTCCTGTTTTTACGGACATTTCAACGTTAAGATCAGGAAGGTCA
GTCAGGTCTCTCAG

CGTCAGGTCCGTGC

2:
TGTGTCCTTTTCTGGTCCATGAGAAGCACGGGACGCCACCTGACGCTGAGAGACCTGACT
GACCTTCCTGATC

3: GGCGTCGGTAGACTGAATCAGATGCATGTACAGACTGCTTGG

4: TTCTCATGGTGCATCTGATTCAGTGAGCTGCTACC

5: TGTACAACCAGAAAAGGACACAGGAGCTTAGCGCGGATTGGGG

6:
ATCCGCACAGACACAGAGACACTTTTTTGGTCTCAACAAGCATCATCCACCTTAAACGACGT
CCAAGCAGTC

7: ACCGCACCCCAGCTAAGCTCCACGTCGTTTATGCGGTAGGTG

8: CCTTGGATTTAACGTTGAAATGTCCGTAAAAACAGGACATCACCAATAGC

9: AACCTAGAGATGATGCTTGTTGAGACCAAAAAAGTGTCTCTGTGTCTGT

TC-Z-7-4-4

1:
CAGCTCGCTATTGGTGATGTCCTGTTTTTACGGACATTTCAACGTTAAGATCAGGAAGGTCA
GTCAGGTCTCTCAG

CGTCAGGTCCGTGC

2:
TGTGTCCTTTTCTGGTCCATGAGAAGCACGGGACGCCACCTGACGCTGAGAGACCTGACT
GACCTTCCTGATC

3: GGCGTCGGTAGACTGAATCAGATGCATGTACAGACTGCTTGG

4: TTCTCATGGTGCATCTGATTCAGTGAGCTGCTACC

5: TGTACAACCAGAAAAGGACACAGGAGCTTAGCGCGGATTGGGG

6:
ATCCGCACAGACACAGAGACACTTTTTTGGTCTCAACAAGCATCATCCACCTTAAACGACGT
CCAAGCAGTC

7: ACCGCACCCCAGCTAAGCTCCACGTCGTTTATGCGGTAGGTG

8: CCATCACTTTAACGTTGATCACCAATAGC

9: CAACTGTAGATGATGCTTGTGTGTCTGT

TC-Z-7-4-6

1:
CAGCTCGCTATTGGTGATGTCCTGTTTTTACGGACATTTCAACGTTAAGATCAGGAAGGTCA
GTCAGGTCTCTCAG

CGTCAGGTCCGTGC

2:
TGTGTCCTTTTCTGGTCCATGAGAAGCACGGGACGCCACCTGACGCTGAGAGACCTGACT
GACCTTCCTGATC

3: GGCGTCGGTAGACTGAATCAGATGCATGTACAGACTGCTTGG

4: TTCTCATGGTGCATCTGATTCAGTGAGCTGCTACC

5: TGTACAACCAGAAAAGGACACAGGAGCTTAGCGCGGATTGGGG

6:
ATCCGCACAGACACAGAGACACTTTTTTGGTCTCAACAAGCATCATCCACCTTAAACGACGT
CCAAGCAGTC

7: ACCGCACCCCAGCTAAGCTCCACGTCGTTTATGCGGTAGGTG

8: CCATCACTTTAACGTTGATCACCAATAGC

9: AACCTAGAGATGATGCTTGTTGAGACCAAAAAAGTGTCTCTGTGTCTGT

TC-Z-8-4-4

1:
CAGCTCGCTATTGGTGATGTCCTGTTTTTACGGACATTTCAACGTTAATCTGTCACCACTCTC
ACCCTGTCTCCGTCGC

2:
TGTGTCCTTTTCTGGTCCATGAGAAGCGACGCCAGCCGAGACAGGGTGAGAGTGGTGACA
GA

3: GGCTGGGGTAGACTGAATCAGATGCATGTACAGACTGCTTGG

4: TTCTCATGGTGCATCTGATTCAGTGAGCTGCTACC

5: TGTACAACCAGAAAAGGACACAGGAGCTTAGCGCGGATTGGGG

6:
ATCCGCACAGACACAGAGACACTTTTTTGGTCTCAACAAGCATCATCCACCTTAAACGACGT
CCAAGCAGTC

7: ACCGCACCCCAGCTAAGCTCCACGTCGTTTATGCGGTAGGTG

8: CCTTGGATTTAACGTTGAAATGTCCGTAAAAACAGGACATCACCAATAGC

9: CAACTGTAGATGATGCTTGTGTGTCTGT

TC-Z-8-4-6

1:
CAGCTCGCTATTGGTGATGTCCTGTTTTTACGGACATTTCAACGTTAATCTGTCACCACTCTC
ACCCTGTCTCCGTCGC

2:
TGTGTCCTTTTCTGGTCCATGAGAAGCGACGCCAGCCGAGACAGGGTGAGAGTGGTGACA
GA

3: GGCTGGGGTAGACTGAATCAGATGCATGTACAGACTGCTTGG

4: TTCTCATGGTGCATCTGATTCAGTGAGCTGCTACC

5: TGTACAACCAGAAAAGGACACAGGAGCTTAGCGCGGATTGGGG

6:
ATCCGCACAGACACAGAGACACTTTTTTGGTCTCAACAAGCATCATCCACCTTAAACGACGT
CCAAGCAGTC

7: ACCGCACCCCAGCTAAGCTCCACGTCGTTTATGCGGTAGGTG

8: CCTTGGATTTAACGTTGAAATGTCCGTAAAAACAGGACATCACCAATAGC

9: AACCTAGAGATGATGCTTGTTGAGACCAAAAAAGTGTCTCTGTGTCTGT

TC-Z-6-4-4

1:
CAGCTCGCTATTGGTGATGTCCTGTTTTTACGGACATTTCAACGTTAATCTGTCACCACTCTC
ACCCTGTCTCCGTCGC

2:
TGTGTCCTTTTCTGGTCCATGAGAAGCGACGCCAGCCGAGACAGGGTGAGAGTGGTGACA
GA

3: GGCTGGGGTAGACTGAATCAGATGCATGTACAGACTGCTTGG

4: TTCTCATGGTGCATCTGATTCAGTGAGCTGCTACC

5: TGTACAACCAGAAAAGGACACAGGAGCTTAGCGCGGATTGGGG

6:
ATCCGCACAGACACAGAGACACTTTTTTGGTCTCAACAAGCATCATCCACCTTAAACGACGT
CCAAGCAGTC

7: ACCGCACCCCAGCTAAGCTCCACGTCGTTTATGCGGTAGGTG

8: CCATCACTTTAACGTTGATCACCAATAGC

9: CAACTGTAGATGATGCTTGTGTGTCTGT

TC-Z-6-4-6

1:
CAGCTCGCTATTGGTGATGTCCTGTTTTTACGGACATTTCAACGTTAATCTGTCACCACTCTC
ACCCTGTCTCCGTCGC

2:
TGTGTCCTTTTCTGGTCCATGAGAAGCGACGCCAGCCGAGACAGGGTGAGAGTGGTGACA
GA

3: GGCTGGGGTAGACTGAATCAGATGCATGTACAGACTGCTTGG

4: TTCTCATGGTGCATCTGATTCAGTGAGCTGCTACC

5: TGTACAACCAGAAAAGGACACAGGAGCTTAGCGCGGATTGGGG

6:
ATCCGCACAGACACAGAGACACTTTTTTGGTCTCAACAAGCATCATCCACCTTAAACGACGT
CCAAGCAGTC

7: ACCGCACCCCAGCTAAGCTCCACGTCGTTTATGCGGTAGGTG

8: CCATCACTTTAACGTTGATCACCAATAGC

9: AACCTAGAGATGATGCTTGTTGAGACCAAAAAAGTGTCTCTGTGTCTGT

Invader and setting strands for SST ring transformation.

Small ring to big ring

Invader strand 1*: ACAGACACAGAGACACTTTTTTGGTCTCAACAAGCATCATCTCTAGGTT

setting strand 1*: CAACTCTAGATGATGCTTGTGTGTCTGT

Big ring to small ring

Invader strand 2*: ACAGACACACAAGCATCATCTAGAGTTG

setting strand 2*: AACCTAGAGATGATGCTTGTTGAGACCAAAAAAGTGTCTCTGTGTCTGT

SST ring 4-2.5-3 with toehold

1: GGCTACAGTCGAAACGTGCACGGATGTCATGGACAACTAGACGCTCTGGGCAAGCAGT

2: CGAGCACGTGAGAGACCAGTCACCATGACATCCTGCTCGGTGCACGTTTCGACT

3: TTGCCCTCTCACTAGCGCTTTTAACCTGACTGGTCTCACGGCTTCACG

4: TAGTTGTGGTTAAAAGCGCTAGCGGTCTTGAGA

5: GCTCCTACAGACACAGAGACACTTTTTTGGTCTCAACAAGCATCATCACTGCAGAGCGTC

6: CAACTCTAGATGATGCTTGTGTGTCTGT

7: CAGCTCGTCAGGACGCTACAGTTCTAATTGGGACTGCGTGACGGAGAATAGACTGGGT

8: ACCGAGAGCATTCGAGAAGTCACCCAATTAGAACTCGGTCTGTAGCGTCCTGAC

9: GTCTATCGTGAGACTGACTTCATGCCTGACTTCTCGAGTAGCCATGCT

10: ACGCAGTGGCATGAAGTCAGTCAGGAGCTCACG

11:
CGCCTAACAGACACAGAGACACTTTTTTGGTCTCAACAAGCATCATCACCCATCTCCGTC

12: CAACTCTAGATGATGCTTGTGTGTCTGT

13: ACTGGCCTCGTATCGCTACCCTCTGTCTCCGTCCTCAGTCTGTAGTCATGTGTGTTCG

14: TCACAACGGAGAGAGCGACGCCACGGAGACAGATTGTGAGGGTAGCGATACGAG

15: ACACATTCGAGTTCTTAGACATGACAGGCGTCGCTCTGAGCTGCTCCG

16: ACTGAGGTGTCATGTCTAAGAATAGGCGCTCGA

17:
GCAGGTACAGACACAGAGACACTTTTTTGGTCTCAACAAGCATCATCCGAACGACTACAG

18: CAACTCTAGATGATGCTTGTGTGTCTGT

19: CTAGGGCGTATCAAGTAACAGGCTACTATCCAGGTCTCTTCGAGGTTGCAATCAGTCT

20: GTCGCATCGATAGTCAACTTGGTGGATAGTAGCTGCGACCTGTTACTTGATACG

21: GATTGCCGCCCAGATTGTCAGTACGTCCAAGTTGACTGCCAGTATCGA

22: AGAGACCACGTACTGACAATCTACCTGCGGGCG

23:
TGGCGAACAGACACAGAGACACTTTTTTGGTCTCAACAAGCATCATCAGACTAACCTCGA

24: CAACTCTAGATGATGCTTGTGTGTCTGT

25: TCACGCCACTGTAGAATTCAAACTGCATTTCATGACTGACGTAGGTGCTCTGAGTGTG

26: GAACGAGACGTATAGGTGTAGGTGAAATGCAGTTCGTTCTTGAATTCTACAGTG

27: TCAGAGACTCACTCTTCAAGTTTTGGCCTACACCTATCCCTAGACGTC

28: TCAGTCACCAAAACTTGAAGAGTCGCCATGAGT

29:
GGATCGACAGACACAGAGACACTTTTTTGGTCTCAACAAGCATCATCCACACCACCTACG

30: CAACTCTAGATGATGCTTGTGTGTCTGT

31: AGCCGTACGTGTCAAAGCCCCTGTATAGCTCCTGGAACTCTCGTGTCCTAACATGTCT

32: ATCGGTCTGGAGTACTGAAGGTGGAGCTATACAACCGATGGGGCTTTGACACGT

33: TGTTAGAGGGTAGTCGAGCTCAGAGGACCTTCAGTACGCGTGATCCAG

34: AGTTCCACCTCTGAGCTCGACTCGATCCACCCT

35:
AGACCGACAGACACAGAGACACTTTTTTGGTCTCAACAAGCATCATCAGACAGACACGAG

36: CAACTCTAGATGATGCTTGTGTGTCTGT

SST ring 6-2.5-3 with toehold

1: GGCTACAGTCGAAACGTGCACGGATGTCATGGACAACTAGACGCTCTGGGCAAGCAGT

2: CGAGCACGTGAGAGACCAGTCACCATGACATCCTGCTCGGTGCACGTTTCGACT

3: TTGCCCTCTCACTAGCGCTTTTAACCTGACTGGTCTCACGGCTTCACG

4: TAGTTGTGGTTAAAAGCGCTAGCGGTCTTGAGA

5: GCTCCTACAGACACAGAGACACTTTTTTGGTCTCAACAAGCATCATCACTGCAGAGCGTC

6: AACCTAGAGATGATGCTTGTTGAGACCAAAAAAGTGTCTCTGTGTCTGT

7: CAGCTCGTCAGGACGCTACAGTTCTAATTGGGACTGCGTGACGGAGAATAGACTGGGT

8: ACCGAGAGCATTCGAGAAGTCACCCAATTAGAACTCGGTCTGTAGCGTCCTGAC

9: GTCTATCGTGAGACTGACTTCATGCCTGACTTCTCGAGTAGCCATGCT

10: ACGCAGTGGCATGAAGTCAGTCAGGAGCTCACG

11:
CGCCTAACAGACACAGAGACACTTTTTTGGTCTCAACAAGCATCATCACCCATCTCCGTC

12: AACCTAGAGATGATGCTTGTTGAGACCAAAAAAGTGTCTCTGTGTCTGT

13: ACTGGCCTTCACTATCTACCCTCTGTCTCTGTCCTCAGTCTGTAGTCATGTGTGTTCG

14: GTCGACAGGAGAGAGCGTATCCACAGAGACAGAGTCGACGGGTAGATAGTGAAG

15: ACACATTCGAGTTCTTAGACATGACAGGATACGCTCTGAGCTGCTCCT

16: ACTGAGGTGTCATGTCTAAGAATAGGCGCTCGA

17:
GCAGGTACAGACACAGAGACACTTTTTTGGTCTCAACAAGCATCATCCGAACGACTACAG

18: AACCTAGAGATGATGCTTGTTGAGACCAAAAAAGTGTCTCTGTGTCTGT

19: CTAGGGCGTATCAAGTAACAGGCTACTATCCAGGTCTCTTCGAGGTTGCAATCAGTCT

20: GTCGCATCGATAGTCAACTTGGTGGATAGTAGCTGCGACCTGTTACTTGATACG

21: GATTGCCGCCCAGATTGTCAGTACGTCCAAGTTGACTGCCAGTATCGA

22: AGAGACCACGTACTGACAATCTACCTGCGGGCG

23:
TGGCGAACAGACACAGAGACACTTTTTTGGTCTCAACAAGCATCATCAGACTAACCTCGA

24: AACCTAGAGATGATGCTTGTTGAGACCAAAAAAGTGTCTCTGTGTCTGT

25: TCACGCCACTGTAGAATTCAAACTGCATTTCATGACTGACGTAGGTGCTCTGAGTGTG

26: GAACGAGACGTATAGGTGTAGGTGAAATGCAGTTCGTTCTTGAATTCTACAGTG

27: TCAGAGACTCACTCTTCAAGTTTTGGCCTACACCTATCCCTAGACGTC

28: TCAGTCACCAAAACTTGAAGAGTCGCCATGAGT

29:
GGATCGACAGACACAGAGACACTTTTTTGGTCTCAACAAGCATCATCCACACCACCTACG

30: AACCTAGAGATGATGCTTGTTGAGACCAAAAAAGTGTCTCTGTGTCTGT

31: AGCCGTACGTGTCAAAGCCCCTGTATAGCTCCTGGAACTCTCGTGTCCTAACATGTCT

32: ATCGGTCTGGAGTACTGAAGGTGGAGCTATACAACCGATGGGGCTTTGACACGT

33: TGTTAGAGGGTAGTCGAGCTCAGAGGACCTTCAGTACGCGTGATCCAG

34: AGTTCCACCTCTGAGCTCGACTCGATCCACCCT

35:
AGACCGACAGACACAGAGACACTTTTTTGGTCTCAACAAGCATCATCAGACAGACACGAG

36: AACCTAGAGATGATGCTTGTTGAGACCAAAAAAGTGTCTCTGTGTCTGT

SST ring 4-2.5-3 without toehold

1: GGCTACAGTCGAAACGTGCACGGATGTCATGGACAACTAGACGCTCTGGGCAAGCAGT

2: CGAGCACGTGAGAGACCAGTCACCATGACATCCTGCTCGGTGCACGTTTCGACT

3: TTGCCCTCTCACTAGCGCTTTTAACCTGACTGGTCTCACGGCTTCACG

4: TAGTTGTGGTTAAAAGCGCTAGCGGTCTTGAGA

5: GCTCCTACAGACACAGAGACACTTTTTTGGTCTCAACAAGCATCATCACTGCAGAGCGTC

6: GATGATGCTTGTGTGTCTGT

7: CAGCTCGTCAGGACGCTACAGTTCTAATTGGGACTGCGTGACGGAGAATAGACTGGGT

8: ACCGAGAGCATTCGAGAAGTCACCCAATTAGAACTCGGTCTGTAGCGTCCTGAC

9: GTCTATCGTGAGACTGACTTCATGCCTGACTTCTCGAGTAGCCATGCT

10: ACGCAGTGGCATGAAGTCAGTCAGGAGCTCACG

11:
CGCCTAACAGACACAGAGACACTTTTTTGGTCTCAACAAGCATCATCACCCATCTCCGTC

12: GATGATGCTTGTGTGTCTGT

13: ACTGGCCTTCACTATCTACCCTCTGTCTCTGTCCTCAGTCTGTAGTCATGTGTGTTCG

14: GTCGACAGGAGAGAGCGTATCCACAGAGACAGAGTCGACGGGTAGATAGTGAAG

15: ACACATTCGAGTTCTTAGACATGACAGGATACGCTCTGAGCTGCTCCT

16: ACTGAGGTGTCATGTCTAAGAATAGGCGCTCGA

17:
GCAGGTACAGACACAGAGACACTTTTTTGGTCTCAACAAGCATCATCCGAACGACTACAG

18: GATGATGCTTGTGTGTCTGT

19: CTAGGGCGTATCAAGTAACAGGCTACTATCCAGGTCTCTTCGAGGTTGCAATCAGTCT

20: GTCGCATCGATAGTCAACTTGGTGGATAGTAGCTGCGACCTGTTACTTGATACG

21: GATTGCCGCCCAGATTGTCAGTACGTCCAAGTTGACTGCCAGTATCGA

22: AGAGACCACGTACTGACAATCTACCTGCGGGCG

23:
TGGCGAACAGACACAGAGACACTTTTTTGGTCTCAACAAGCATCATCAGACTAACCTCGA

24: GATGATGCTTGTGTGTCTGT

25: TCACGCCACTGTAGAATTCAAACTGCATTTCATGACTGACGTAGGTGCTCTGAGTGTG

26: GAACGAGACGTATAGGTGTAGGTGAAATGCAGTTCGTTCTTGAATTCTACAGTG

27: TCAGAGACTCACTCTTCAAGTTTTGGCCTACACCTATCCCTAGACGTC

28: TCAGTCACCAAAACTTGAAGAGTCGCCATGAGT

29:
GGATCGACAGACACAGAGACACTTTTTTGGTCTCAACAAGCATCATCCACACCACCTACG

30: GATGATGCTTGTGTGTCTGT

31: AGCCGTACGTGTCAAAGCCCCTGTATAGCTCCTGGAACTCTCGTGTCCTAACATGTCT

32: ATCGGTCTGGAGTACTGAAGGTGGAGCTATACAACCGATGGGGCTTTGACACGT

33: TGTTAGAGGGTAGTCGAGCTCAGAGGACCTTCAGTACGCGTGATCCAG

34: AGTTCCACCTCTGAGCTCGACTCGATCCACCCT

35:
AGACCGACAGACACAGAGACACTTTTTTGGTCTCAACAAGCATCATCAGACAGACACGAG

36: GATGATGCTTGTGTGTCTGT

SST ring 6-2.5-3 without toehold

1: GGCTACAGTCGAAACGTGCACGGATGTCATGGACAACTAGACGCTCTGGGCAAGCAGT

2: CGAGCACGTGAGAGACCAGTCACCATGACATCCTGCTCGGTGCACGTTTCGACT

3: TTGCCCTCTCACTAGCGCTTTTAACCTGACTGGTCTCACGGCTTCACG

4: TAGTTGTGGTTAAAAGCGCTAGCGGTCTTGAGA

5: GCTCCTACAGACACAGAGACACTTTTTTGGTCTCAACAAGCATCATCACTGCAGAGCGTC

6: GATGATGCTTGTTGAGACCAAAAAAGTGTCTCTGTGTCTGT

7: CAGCTCGTCAGGACGCTACAGTTCTAATTGGGACTGCGTGACGGAGAATAGACTGGGT

8: ACCGAGAGCATTCGAGAAGTCACCCAATTAGAACTCGGTCTGTAGCGTCCTGAC

9: GTCTATCGTGAGACTGACTTCATGCCTGACTTCTCGAGTAGCCATGCT

10: ACGCAGTGGCATGAAGTCAGTCAGGAGCTCACG

11:
CGCCTAACAGACACAGAGACACTTTTTTGGTCTCAACAAGCATCATCACCCATCTCCGTC

12: GATGATGCTTGTTGAGACCAAAAAAGTGTCTCTGTGTCTGT

13: ACTGGCCTTCACTATCTACCCTCTGTCTCTGTCCTCAGTCTGTAGTCATGTGTGTTCG

14: GTCGACAGGAGAGAGCGTATCCACAGAGACAGAGTCGACGGGTAGATAGTGAAG

15: ACACATTCGAGTTCTTAGACATGACAGGATACGCTCTGAGCTGCTCCT

16: ACTGAGGTGTCATGTCTAAGAATAGGCGCTCGA

17:
GCAGGTACAGACACAGAGACACTTTTTTGGTCTCAACAAGCATCATCCGAACGACTACAG

18: GATGATGCTTGTTGAGACCAAAAAAGTGTCTCTGTGTCTGT

19: CTAGGGCGTATCAAGTAACAGGCTACTATCCAGGTCTCTTCGAGGTTGCAATCAGTCT

20: GTCGCATCGATAGTCAACTTGGTGGATAGTAGCTGCGACCTGTTACTTGATACG

21: GATTGCCGCCCAGATTGTCAGTACGTCCAAGTTGACTGCCAGTATCGA

22: AGAGACCACGTACTGACAATCTACCTGCGGGCG

23:
TGGCGAACAGACACAGAGACACTTTTTTGGTCTCAACAAGCATCATCAGACTAACCTCGA

24: GATGATGCTTGTTGAGACCAAAAAAGTGTCTCTGTGTCTGT

25: TCACGCCACTGTAGAATTCAAACTGCATTTCATGACTGACGTAGGTGCTCTGAGTGTG

26: GAACGAGACGTATAGGTGTAGGTGAAATGCAGTTCGTTCTTGAATTCTACAGTG

27: TCAGAGACTCACTCTTCAAGTTTTGGCCTACACCTATCCCTAGACGTC

28: TCAGTCACCAAAACTTGAAGAGTCGCCATGAGT

29:
GGATCGACAGACACAGAGACACTTTTTTGGTCTCAACAAGCATCATCCACACCACCTACG

30: GATGATGCTTGTTGAGACCAAAAAAGTGTCTCTGTGTCTGT

31: AGCCGTACGTGTCAAAGCCCCTGTATAGCTCCTGGAACTCTCGTGTCCTAACATGTCT

32: ATCGGTCTGGAGTACTGAAGGTGGAGCTATACAACCGATGGGGCTTTGACACGT

33: TGTTAGAGGGTAGTCGAGCTCAGAGGACCTTCAGTACGCGTGATCCAG

34: AGTTCCACCTCTGAGCTCGACTCGATCCACCCT

35:
AGACCGACAGACACAGAGACACTTTTTTGGTCTCAACAAGCATCATCAGACAGACACGAG

36: GATGATGCTTGTTGAGACCAAAAAAGTGTCTCTGTGTCTGT

Section 4 Supplementary tables

Supplementary Table 1 | The calculated N value of four pairs of 4-state monomers

Upper swing arm (turns)	lower swing arm (turns)	support arm (turns)	L2 (nm)	R (nm)	L1 (nm)	r1 (nm)	C1 (nm)	N	Inner D(nm)	outer D(nm)
9	4.00	5.50	31.37	21.17	13.94	16.94	106.37	7.63	31.88	78.22
9	6.00	5.50	31.37	21.17	20.92	42.35	265.93	12.71	82.69	129.04
7	4.00	5.50	24.40	21.17	13.94	28.23	177.29	12.71	54.46	100.81
7	6.00	5.50	24.40	21.17	20.92	127.04	797.80	38.14	252.08	298.42
9	4.00	3.50	31.37	14.20	13.94	11.36	71.35	5.12	20.72	53.12
9	6.00	3.50	31.37	14.20	20.92	28.40	178.36	8.53	54.80	87.21
7	4.00	3.50	24.40	14.20	13.94	18.93	118.91	8.53	35.87	68.27
7	6.00	3.50	24.40	14.20	20.92	85.21	535.09	25.58	168.41	200.81
9	4.00	4.00	31.37	15.94	13.94	12.76	80.10	5.74	23.51	59.40
9	6.00	4.00	31.37	15.94	20.92	31.89	200.26	9.57	61.78	97.66
7	4.00	4.00	24.40	15.94	13.94	21.26	133.50	9.57	40.52	76.41
7	6.00	4.00	24.40	15.94	20.92	95.66	600.77	28.72	189.33	225.22
8	4.00	4.00	27.89	15.94	13.94	15.94	100.13	7.18	29.89	65.78
8	6.00	4.00	27.89	15.94	20.92	47.83	300.38	14.36	93.66	129.55
6	4.00	4.00	20.92	15.94	13.94	31.89	200.26	14.36	61.78	97.66
6	6.00	4.00	20.92	15.94	20.92					

Supplementary Table 2 | The calculated hybridization energy of sticky ends of TC 1-1 and TC 1-2.

Tile's name	sticky end sequence	$\Delta G_{initiation}$	$\Delta G_{symmetry}$	$\Delta G_{stacking}$	ΔG_{ATterm}	ΔG kcal mol^{-1}
TC-1-1	GTC	1.96	0	-2.74	0	-0.78
TC-1-1	GAC	1.96	0	-2.74	0	-0.78
TC-1-1	CTCATC	1.96	0	-6.61	0	-4.65
TC-1-1	GATGAG	1.96	0	-6.21	0	-4.25
TC-1-1, TC-1-2	CTAG	1.96	0.43	-3.14	0	-0.75
TC-1-2	GAGGTG	1.96	0	-7.31	0	-5.35
TC-1-2	GCGC	1.96	0.43	-6.65	0	-4.26
TC-1-2	TGCA	1.96	0.43	-5.14	0.05	-2.7
TC-1-2	CACCTC	1.96	0	-7.31	0	-5.35

Supplementary Table 3 | The calculated hybridization energy of sticky ends of TC 2-1 and TC 2-2.

Tile's name	sticky end sequence	$\Delta G_{initiation}$	$\Delta G_{symmetry}$	$\Delta G_{stacking}$	ΔG_{ATterm}	ΔG kcal mol^{-1}
TC-2-1	GTC	1.96	0	-2.74	0	-0.78
TC-2-1	GAC	1.96	0	-2.74	0	-0.78
TC-2-1	CTCATC	1.96	0	-6.61	0	-4.65
TC-2-1	GATGAG	1.96	0	-6.21	0	-4.25
TC-2-1, TC-2-2	CTAG	1.96	0.43	-3.14	0	-0.75
TC-2-2	CTCCAC	1.96	0	-7.31	0	-5.35
TC-2-2	GCGC	1.96	0.43	-6.65	0	-4.26
TC-2-2	TGCA	1.96	0.43	-5.14	0.1	-2.65
TC-2-2	GTGGAG	1.96	0	-7.31	0	-5.35

Supplementary Table 4 | The calculated hybridization energy of sticky ends of TC 3-1, TC 3-2 and TC 3-3.

Tile's name	sticky end sequence	$\Delta G_{initiation}$	$\Delta G_{symmetry}$	$\Delta G_{stacking}$	ΔG_{ATterm}	ΔG kcal mol^{-1}
TC-3-1, TC-3-2	TGAGCG	1.96	0	-8.44	0.05	-6.43
TC-3-1, TC-3-2	CGCTCA	1.96	0	-8.44	0.05	-6.43
TC-3-1, TC-3-2, TC-3-3	ATCCGC	1.96	0	-8.43	0.05	-6.42
TC-3-1, TC-3-2, TC-3-3	GCGGAT	1.96	0	-8.43	0.05	-6.42
TC-3-3	TCG	1.96	0	-3.47	0.05	-1.46
TC-3-3	CGA	1.96	0	-3.47	0.05	-1.46

Supplementary Table 5 | The calculated hybridization energy of sticky ends of TC 4-1 and TC 4-2.

Tile's name	sticky end sequence	$\Delta G_{initiation}$	$\Delta G_{symmetry}$	$\Delta G_{stacking}$	ΔG_{ATterm}	ΔG kcal mol^{-1}
TC-4-1	CTTCTC	1.96	0	-6.16	0	-4.2
TC-4-1	GTAC	1.96	0.43	-3.46	0	-1.07
TC-4-1	GAGAAG	1.96	0	-6.16	0	-4.2
TC-4-1	CTTA	1.96	0	-2.86	0.05	-0.85
TC-4-1	TAAG	1.96	0	-2.86	0.05	-0.85
TC-4-1/TC-4-2	GGCC	1.96	0.43	-5.92	0	-3.53
TC-4-2	GAGGTG	1.96	0	-7.31	0	-5.35
TC-4-2	CGCG	1.96	0.43	-6.58	0	-4.19
TC-4-2	GATC	1.96	0.43	-3.48	0	-1.09
TC-4-2	CACCTC	1.96	0	-7.31	0	-5.35
TC-4-2	CATG	1.96	0.43	-3.61	0	-1.22

Supplementary Table 6 | The calculated hybridization energy of sticky ends of TC 5-1, TC 5-2, TC 5-3 and TC 5-4.

Tile's name	sticky end sequence	$\Delta G_{initiation}$	$\Delta G_{symmetry}$	$\Delta G_{stacking}$	ΔG_{ATterm}	ΔG kcal mol^{-1}
TC-5-1, TC-5-2	CTTG	1.96	0	-3.73	0	-1.77
TC-5-1, TC-5-2	CAAG	1.96	0	-3.73	0	-1.77
TC-5-1, TC-5-2, TC-5-3, TC-5-4	CTTGCA	1.96	0	-7.42	0.05	-5.41
TC-5-1, TC-5-2, TC-5-3, TC-5-4	TGCAAG	1.96	0	-7.42	0.05	-5.41
TC-5-1, TC-5-2, TC-5-3, TC-5-4	GAAGGG	1.96	0	-7.26	0	-5.3
TC-5-1, TC-5-2, TC-5-3, TC-5-4	CCCTTC	1.96	0	-7.26	0	-5.3
TC-5-3, TC-5-4	CAAGCC	1.96	0	-7.81	0	-5.85
TC-5-3, TC-5-4	GGCTTG	1.96	0	-7.81	0	-5.85

Supplementary Table 7 | The calculated hybridization energy of sticky ends of TC 6-1 and TC 6-2.

Tile's name	sticky end sequence	$\Delta G_{initiation}$	$\Delta G_{symmetry}$	$\Delta G_{stacking}$	ΔG_{ATterm}	ΔG kcal mol^{-1}
TC-6-1	CCTAGG	1.96	0.43	-6.82	0	-4.43
TC-6-1	GTAAC	1.96	0	-4.46	0	-2.5
TC-6-1	GTTAC	1.96	0	-4.46	0	-2.5
TC-6-1	GCACT	1.96	0	-6.41	0.05	-4.4
TC-6-1	AGTGC	1.96	0	-6.41	0.05	-4.4
TC-6-2	TCACG	1.96	0	-6.36	0.05	-4.35
TC-6-2	CAATG	1.96	0	-4.78	0	-2.82
TC-6-2	CATTG	1.96	0	-4.78	0	-2.82
TC-6-2	CGTGA	1.96	0	-6.36	0.05	-4.35
TC-6-2	GGATCC	1.96	0.43	-7.16	0	-4.77

Supplementary Table 8 | The calculated hybridization energy of sticky ends of TC 7-1 and TC 7-2.

Tile's name	sticky end sequence	$\Delta G_{initiation}$	$\Delta G_{symmetry}$	$\Delta G_{stacking}$	ΔG_{ATterm}	ΔG kcal mol^{-1}
TC-7-1, TC-7-2	GGATCC	1.96	0.43	-7.16	0	-4.77
TC-7-1, TC-7-2	GACGTC	1.96	0.43	-7.65	0	-5.26
TC-7-1, TC-7-2	TGAGC	1.96	0	-6.27	0.05	-4.26
TC-7-1, TC-7-2	CAATG	1.96	0	-4.78	0	-2.82
TC-7-1, TC-7-2	CATTG	1.96	0	-4.78	0	-2.82
TC-7-1, TC-7-2	GCTCA	1.96	0	-5.27	0.05	-3.26

Supplementary Table 9 | The calculated hybridization energy of sticky ends of TC 8-1, TC 8-2, TC 8-3, and TC 8-4.

Tile's name	sticky end sequence	$\Delta G_{initiation}$	$\Delta G_{symmetry}$	$\Delta G_{stacking}$	ΔG_{ATterm}	ΔG kcal mol^{-1}
TC-8-1, TC-8-2, TC-8-3	GCGC	1.96	0.43	-6.65	0	-4.26
TC-8-1, TC-8-2, TC-8-3	TGCA	1.96	0.43	-5.14	0.1	-2.65
TC-8-1, TC-8-2, TC-8-3	CGCG	1.96	0.43	-6.58	0	-4.19
TC-8-1, TC-8-2, TC-8-3	CATG	1.96	0.43	-3.78	0	-1.39
TC-8-2, TC-8-3, TC-8-4	CTAG	1.96	0.43	-3.44	0	-1.05
TC-8-3	GGCC	1.96	0.43	-5.92	0	-3.53
TC-8-4	GC	1.96	0.43	-2.24	0	0.15
TC-8-4	AT	1.96	0.43	-0.88	0.05	1.56
TC-8-4	TCGA	1.96	0.43	-4.77	0.1	-2.28
TC-8-4	CG	1.96	0.43	-2.17	0	0.22
TC-8-4	TA	1.96	0.43	-0.58	0.1	1.91

Supplementary Table 10 | The calculated hybridization energy of sticky ends of TC 9-1, TC 9-2, and TC 9-3.

Tile's name	sticky end sequence	$\Delta G_{initiation}$	$\Delta G_{symmetry}$	$\Delta G_{stacking}$	ΔG_{ATterm}	ΔG kcal mol^{-1}
TC-9-1, TC-9-2, TC-9-3	GCGC	1.96	0.43	-6.65	0	-4.26
TC-9-1, TC-9-2, TC-9-3	TGCA	1.96	0.43	-5.14	0.1	-2.65
TC-9-1, TC-9-2, TC-9-3	CGCG	1.96	0.43	-6.58	0	-4.19
TC-9-1, TC-9-2, TC-9-3	CATG	1.96	0.43	-3.78	0	-1.39
TC-9-2, TC-9-3	CTAG	1.96	0.43	-3.14	0	-0.75
TC-9-3	GGCC	1.96	0.43	-5.92	0	-3.53

Supplementary Table 11 | The calculated hybridization energy of sticky ends of defined ring.

Tile's name	sticky end sequence	$\Delta G_{initiation}$	$\Delta G_{symmetry}$	$\Delta G_{stacking}$	ΔG_{ATterm}	ΔG kcal mol^{-1}
Defined ring	ATCCGC	1.96	0	-8.43	0.05	-6.42
Defined ring	GCGGAT	1.96	0	-8.43	0.05	-6.42
Defined ring	CAGCTG	1.96	0	-7.7	0	-5.74
Defined ring	CAGCTG	1.96	0	-7.7	0	-5.74

Supplementary Table 12 | The calculated hybridization energy of sticky ends of dynamic ring. Note the four pairs of dynamic nanorings share the same sticky ends.

Tile's name	sticky end sequence	$\Delta G_{initiation}$	$\Delta G_{symmetry}$	$\Delta G_{stacking}$	ΔG_{ATterm}	ΔG kcal mol^{-1}
Dynamic ring	CAGCTC	1.96	0	-7.55	0	-5.59
Dynamic ring	GAGCTG	1.96	0	-7.55	0	-5.59
Dynamic ring	GCGGAT	1.96	0	-8.43	0.05	-6.42
Dynamic ring	ATCCGC	1.96	0	-8.43	0.05	-6.42

Supplementary Table 12 | The calculated hybridization energy of sticky ends of SST ring.

Tile's name	sticky end sequence	$\Delta G_{initiation}$	$\Delta G_{symmetry}$	$\Delta G_{stacking}$	ΔG_{ATterm}	ΔG kcal mol^{-1}
SST ring	GGCTAC	1.96	0	-7.38	0	-5.42
SST ring	ACGGCT	1.96	0	-8.97	0.05	-6.96
SST ring	CGGTCT	1.96	0	-8.03	0.05	-6.02
SST ring	GCTCCT	1.96	0	-7.94	0.05	-5.93
SST ring	CAGCTC	1.96	0	-7.55	0	-5.59
SST ring	GTAGCC	1.96	0	-7.38	0	-5.42
SST ring	AGGAGC	1.96	0	-7.94	0	-5.98
SST ring	CGCCTA	1.96	0	-8.11	0.05	-6.1
SST ring	ACTGGC	1.96	0	-8.25	0.05	-6.24
SST ring	GAGCTG	1.96	0	-7.55	0	-5.59
SST ring	TAGGCG	1.96	0	-8.11	0.05	-6.1
SST ring	GCAGGT	1.96	0	-8.25	0.05	-6.24
SST ring	CTAGGG	1.96	0	-6.82	0	-4.86
SST ring	GCCAGT	1.96	0	-8.25	0.05	-6.24
SST ring	ACCTGC	1.96	0	-8.25	0.05	-6.24
SST ring	TGGCGA	1.96	0	-9	0.1	-6.94
SST ring	TCACGC	1.96	0	-8.6	0.05	-6.59
SST ring	CCCTAG	1.96	0	-6.82	0	-4.86
SST ring	TCGCCA	1.96	0	-9	0.1	-6.94
SST ring	GGATCG	1.96	0	-7.49	0	-5.53
SST ring	AGCCGT	1.96	0	-8.97	0.1	-6.91
SST ring	GCGTGA	1.96	0	-8.6	0.05	-6.59
SST ring	CGATCC	1.96	0	-7.49	0	-5.53
SST ring	AGACCG	1.96	0	-8.03	0.05	-6.02

Supplementary Table 13 | The invader and setting strands information of each transition of four pairs of dynamic nanorings. Sequences of invader and setting strands are summarized in section 3 Supplementary Notes.

pair	path	State 1 monomer tile	State 2 monomer tile	invader strand	setting strand
pair 1	path 1	TC-C-7-3.5-6	TC-C-7-3.5-4	invader strand 2	setting strand 2
	path 2	TC-C-7-3.5-6	TC-C-9-3.5-6	invader strand 3	setting strand 3
	path 3	TC-C-7-3.5-6	TC-C-9-3.5-4	invader strand 2&3	setting strand 2&3
	path 4	TC-C-9-3.5-6	TC-C-7-3.5-6	invader strand 1	setting strand 1
	path 5	TC-C-9-3.5-6	TC-C-9-3.5-4	invader strand 2	setting strand 2
	path 6	TC-C-9-3.5-6	TC-C-7-3.5-4	invader strand 1&2	setting strand 1&2
	path 7	TC-C-7-3.5-4	TC-C-9-3.5-6	invader strand 3&4	setting strand 3&4
	path 8	TC-C-7-3.5-4	TC-C-7-3.5-6	invader strand 4	setting strand 4
	path 9	TC-C-7-3.5-4	TC-C-9-3.5-4	invader strand 3	setting strand 3
	path 10	TC-C-9-3.5-4	TC-C-7-3.5-4	invader strand 1	setting strand 1
	path 11	TC-C-9-3.5-4	TC-C-9-3.5-6	invader strand 4	setting strand 4
	path 12	TC-C-9-3.5-4	TC-C-7-3.5-6	invader strand 1&4	setting strand 1&4
pair 2	path 1	TC-C-7-5.5-6	TC-C-7-5.5-4	invader strand 2	setting strand 2
	path 2	TC-C-7-5.5-6	TC-C-9-5.5-6	invader strand 3	setting strand 3
	path 3	TC-C-7-5.5-6	TC-C-9-5.5-4	invader strand 2&3	setting strand 2&3
	path 4	TC-C-9-5.5-6	TC-C-7-5.5-6	invader strand 1	setting strand 1
	path 5	TC-C-9-5.5-6	TC-C-9-5.5-4	invader strand 2	setting strand 2
	path 6	TC-C-9-5.5-6	TC-C-7-5.5-4	invader strand 1&2	setting strand 1&2
	path 7	TC-C-7-5.5-4	TC-C-9-5.5-6	invader strand 3&4	setting strand 3&4
	path 8	TC-C-7-5.5-4	TC-C-7-5.5-6	invader strand 4	setting strand 4
	path 9	TC-C-7-5.5-4	TC-C-9-5.5-4	invader strand 3	setting strand 3
	path 10	TC-C-9-5.5-4	TC-C-7-5.5-4	invader strand 1	setting strand 1
	path 11	TC-C-9-5.5-4	TC-C-9-5.5-6	invader strand 4	setting strand 4
	path 12	TC-C-9-5.5-4	TC-C-7-5.5-6	invader strand 1&4	setting strand 1&4
pair 3	path 1	TC-Z-7-4-6	TC-Z-7--4-4	invader strand 2	setting strand 2
	path 2	TC-Z-7-4-6	TC-Z-9--4-6	invader strand 3	setting strand 3
	path 3	TC-Z-7-4-6	TC-Z-9--4-4	invader strand 2&3	setting strand 2&3
	path 4	TC-Z-9-4-6	TC-Z-7--4-6	invader strand 1	setting strand 1
	path 5	TC-Z-9-4-6	TC-Z-9--4-4	invader strand 2	setting strand 2
	path 6	TC-Z-9-4-6	TC-Z-7--4-4	invader strand 1&2	setting strand 1&2
	path 7	TC-Z-7-4-4	TC-Z-9--4-6	invader strand 3&4	setting strand 3&4
	path 8	TC-Z-7-4-4	TC-Z-7--4-6	invader strand 4	setting strand 4
	path 9	TC-Z-7-4-4	TC-Z-9--4-4	invader strand 3	setting strand 3
	path 10	TC-Z-9-4-4	TC-Z-7--4-4	invader strand 1	setting strand 1
	path 11	TC-Z-9-4-4	TC-Z-9--4-6	invader strand 4	setting strand 4
	path 12	TC-Z-9-4-4	TC-Z-7--4-6	invader strand 1&4	setting strand 1&4
pair 4	path 1	TC-Z-8-4-6	TC-Z-6--4-4	invader strand 2	setting strand 2
	path 2	TC-Z-8-4-6	TC-Z-8--4-6	invader strand 3	setting strand 3
	path 3	TC-Z-8-4-6	TC-Z-8--4-4	invader strand 2&3	setting strand 2&3
	path 4	TC-Z-8-4-6	TC-Z-6--4-6	invader strand 1	setting strand 1
	path 5	TC-Z-8-4-6	TC-Z-8--4-4	invader strand 2	setting strand 2
	path 6	TC-Z-8-4-6	TC-Z-6--4-4	invader strand 1&2	setting strand 1&2
	path 7	TC-Z-8-4-4	TC-Z-8--4-6	invader strand 3&4	setting strand 3&4
	path 8	TC-Z-8-4-4	TC-Z-6--4-6	invader strand 4	setting strand 4
	path 9	TC-Z-8-4-4	TC-Z-8--4-4	invader strand 3	setting strand 3
	path 10	TC-Z-8-4-4	TC-Z-6--4-4	invader strand 1	setting strand 1
	path 11	TC-Z-8-4-4	TC-Z-8--4-6	invader strand 4	setting strand 4
	path 12	TC-Z-8-4-4	TC-Z-6--4-6	invader strand 1&4	setting strand 1&4

Supplementary Table 14 | The invader and setting strands information of SST nanorings transformation. Sequences of invader and setting strands are summarized in section 3 Supplementary Notes.

	State1 monomer tile	State 2 monomer tile	invader strand	setting strand
SST nanorings	TC-C-6-2.5-3	TC-C-4-2.5-3	invader strand 1*	setting strand 1*
	TC-C-4-2.5-3	TC-C-6-2.5-3	invader strand 2*	setting strand 2*

Section 5 Supplementary references

1. SantaLucia, J., Jr. and D. Hicks, *The thermodynamics of DNA structural motifs.* Annu Rev Biophys Biomol Struct, 2004. **33**: p. 415-40.

Section 6 Source data of Supplementary information

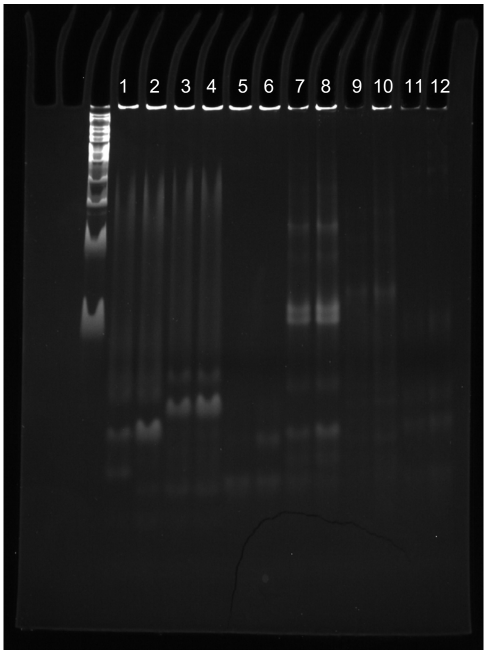

Supplementary Source Figure 1. Original gel images of Supplementary Figure 1c, 5c, and 9c. Lane 8, Supplementary Figure 1c, TC-1-1, 66 h; Lane 10, Supplementary Figure 5c, TC-2-1, 66 h; Lane12, Supplementary Figure 9c, TC-4-1, 66 h.

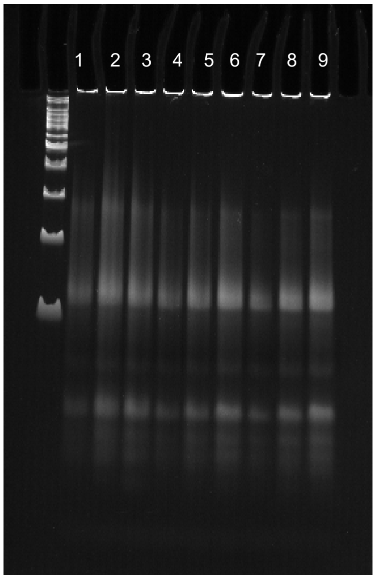

Supplementary Source Figure 2. Original gel images of Supplementary Figure 2c. Lane 2, TC-1-2, 2 h; Lane 5, TC-1-2, 16 h; Lane 8, TC-1-2, 66 h.

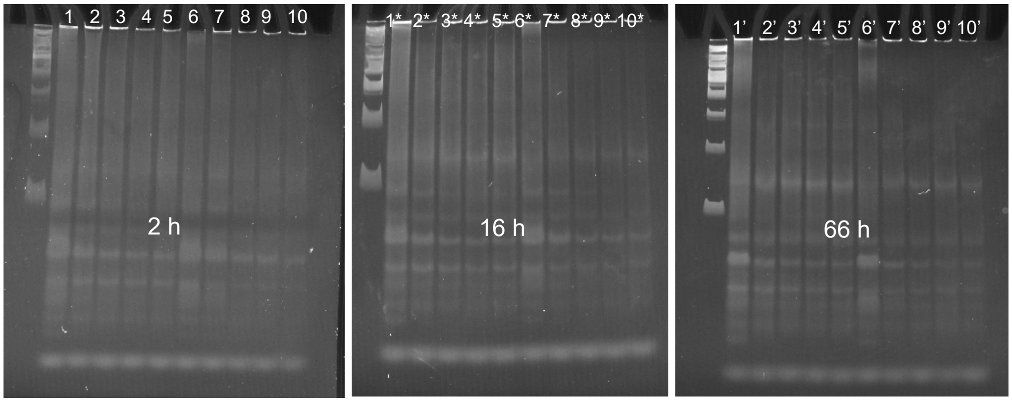

Supplementary Source Figure 3. Original gel images of Supplementary Figure 6c. Lane 9, Supplementary Figure 6c, TC-2-2, 2 h; Lane 9*, Supplementary Figure 6c, TC-2-2, 16 h; Lane 9’, Supplementary Figure 6c, TC-2-2, 66 h.

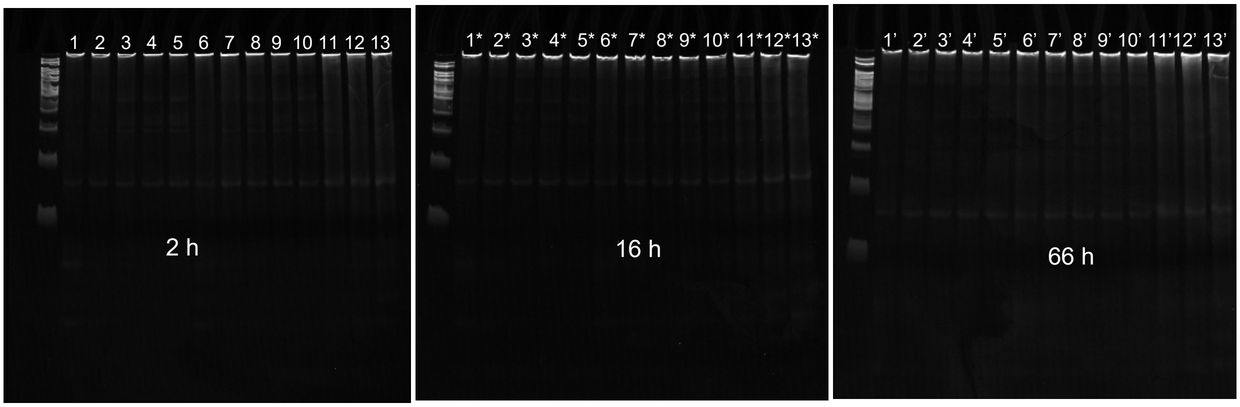

Supplementary Source Figure 4. Original gel images of Supplementary Figure 7c. Lane 10, Supplementary Figure 7c, TC-3-1, 2 h; Lane 8*, Supplementary Figure 7c, TC-3-1, 16 h; Lane 10’, Supplementary Figure 7c, TC-3-1, 66 h.

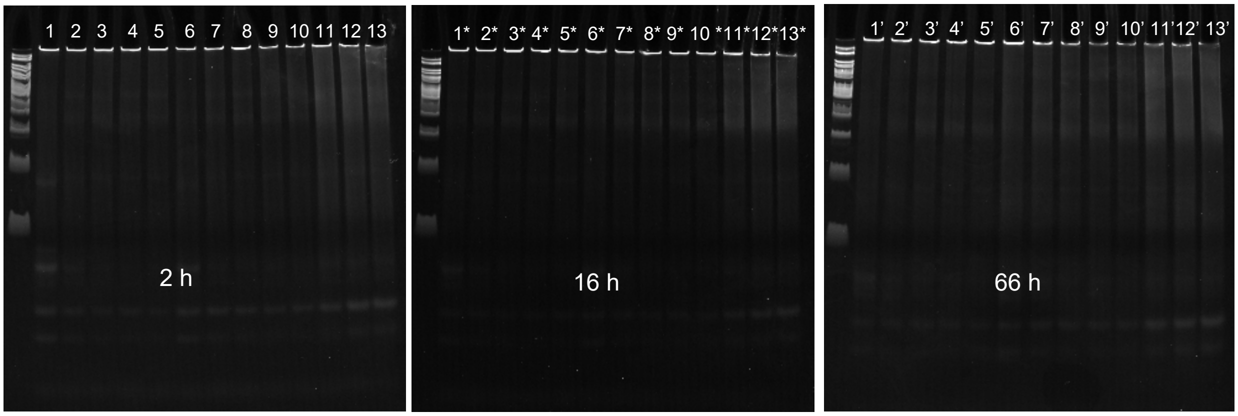

Supplementary Source Figure 5. Original gel images of Supplementary Figure 8c. Lane 11, Supplementary Figure 8c, TC-3-2, 2 h; Lane 10*, Supplementary Figure 8c, TC-3-2, 16 h; Lane 10’, Supplementary Figure 8c, TC-3-2, 66 h.

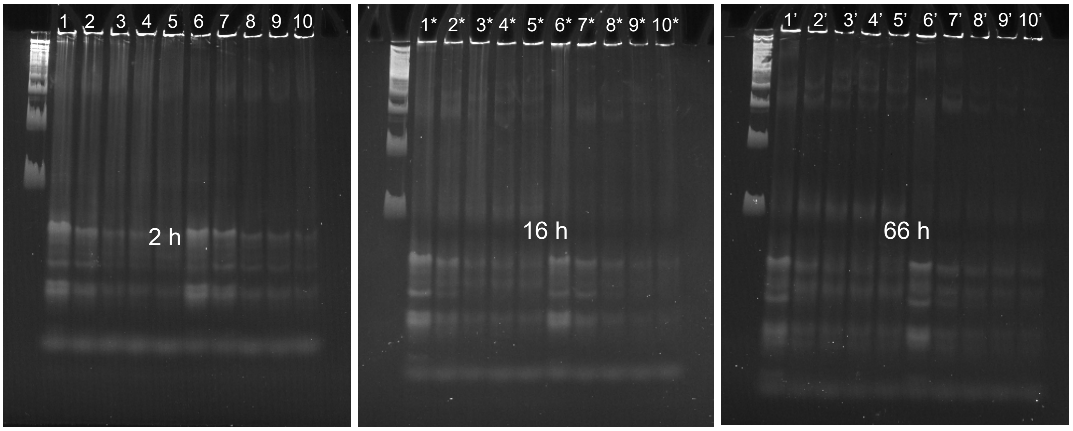

Supplementary Source Figure 6. Original gel images of Supplementary Figure 10c. Lane 8, Supplementary Figure 10c, TC-4-2, 2 h; Lane 8*, Supplementary Figure 10c, TC-4-2, 16 h; Lane 8’, Supplementary Figure 10c, TC-4-2, 66 h.

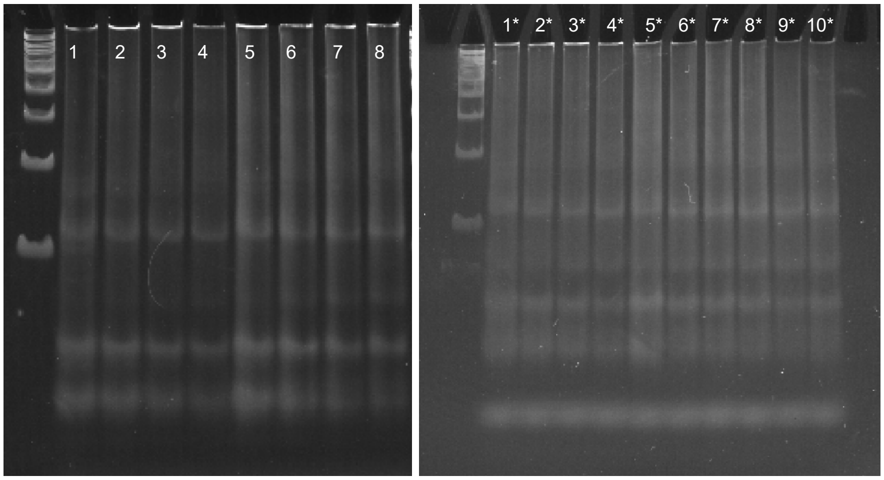

Supplementary Source Figure 7. Original gel images of Supplementary Figure 12c. Lane 7, Supplementary Figure 12c, TC-8-3, 2 h; Lane 7*, Supplementary Figure 12c, TC-8-3, 16 h; Lane 9*, Supplementary Figure 12c, TC-8-3, 66 h.

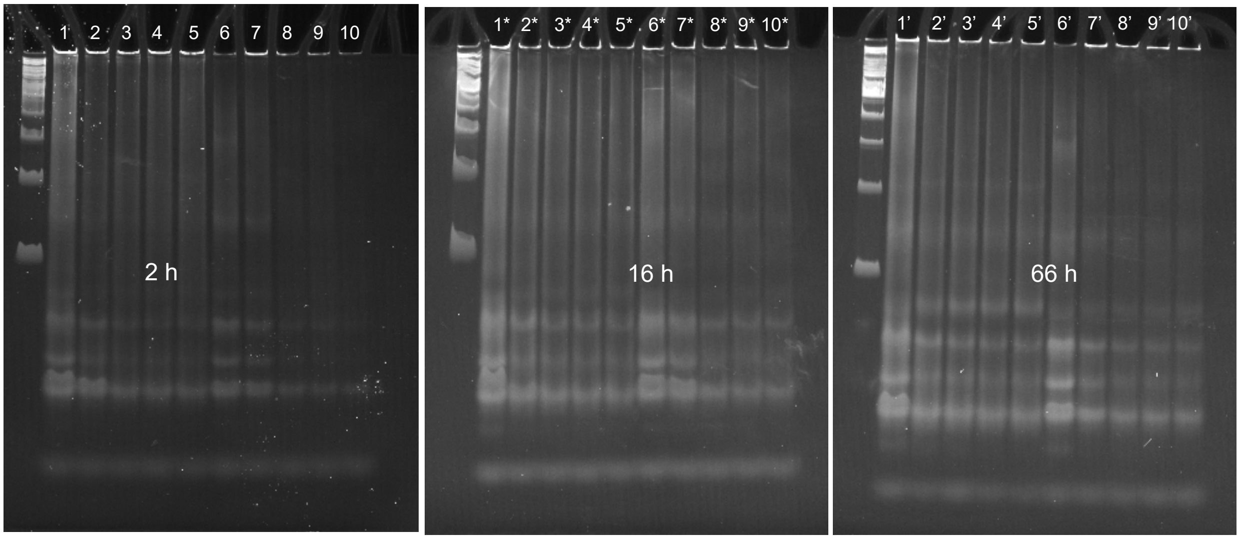

Supplementary Source Figure 8. Original gel images of Supplementary Figure 13c. Lane 8, Supplementary Figure 13c, TC-9-3, 2 h; Lane 8*, Supplementary Figure 13c, TC-9-3, 16 h; Lane 8’, Supplementary Figure 13c, TC-9-3, 66 h.

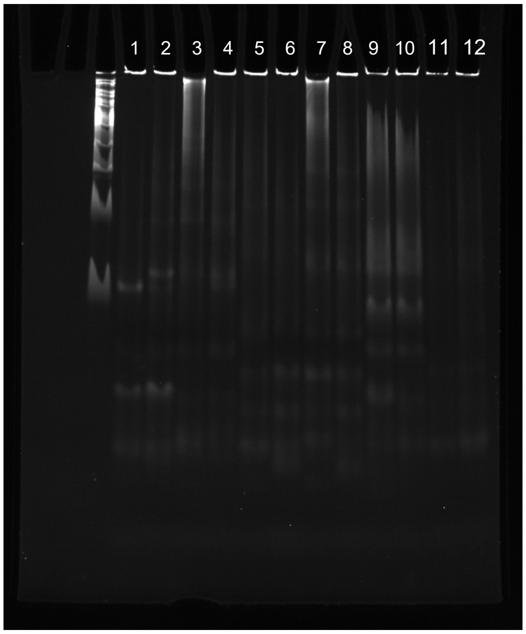

Supplementary Source Figure 9. Original gel images of Supplementary Figure 15c, 16c, 17c, 18c, and 19b. Lane 2, Supplementary Figure 16 c, TC-8-2, 66 h; Lane 3, Supplementary Figure 15 c, TC-8-1, 66 h; Lane 6, Supplementary Figure 18 c, TC-9-2, 66 h; Lane 7, Supplementary Figure 17 c, TC-9-1, 66 h; Lane 10, Supplementary Figure 19 b, TC-8-4, 66 h.

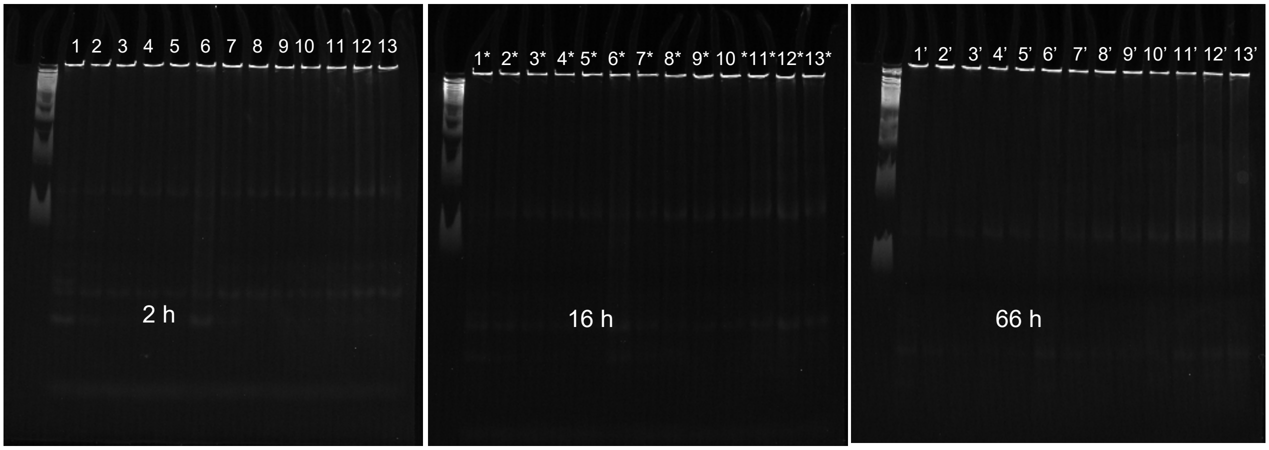

Supplementary Source Figure 10. Original gel images of Supplementary Figure 22c. Lane 11, Supplementary Figure 22c, TC-5-1, 2 h; Lane 11*, Supplementary Figure 22c, TC-5-1, 16 h; Lane 11', Supplementary Figure 22c, TC-5-1, 66 h.

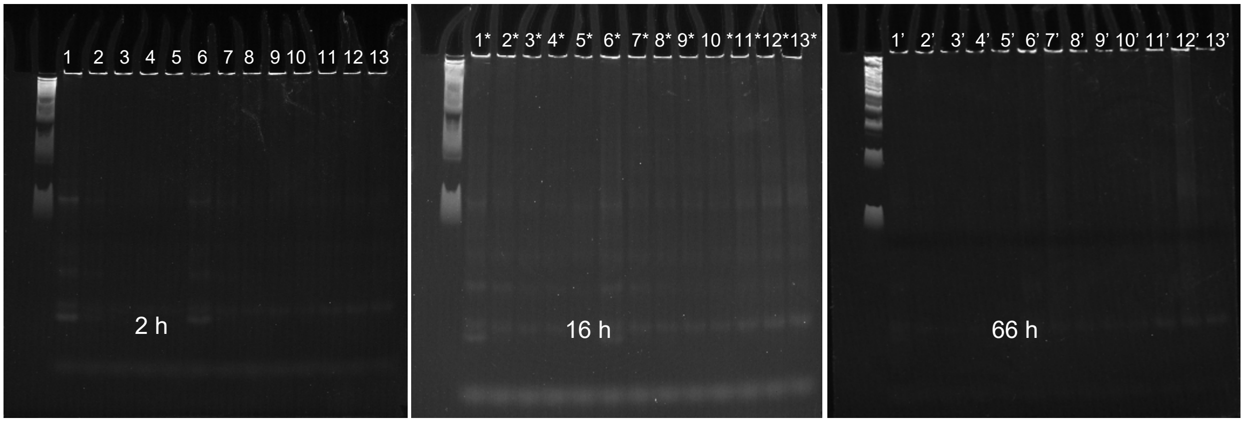

Supplementary Source Figure 11. Original gel images of Supplementary Figure 23c. Lane 10, Supplementary Figure 23c, TC-5-2, 2 h; Lane 10*, Supplementary Figure 23c, TC-5-2, 16 h; Lane 10', Supplementary Figure 23c, TC-5-2, 66 h.

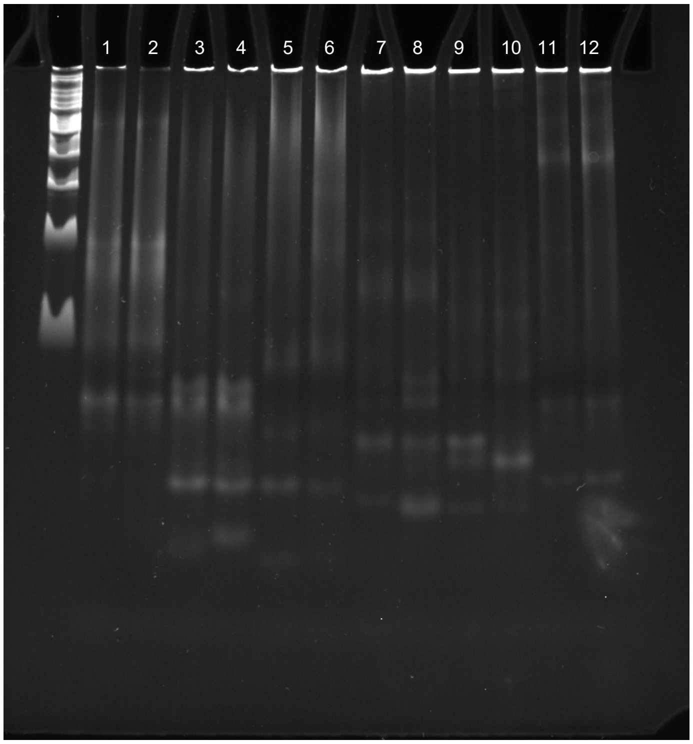

Supplementary Source Figure 12. Original gel images of Supplementary Figure 26 c. Lane 3, Supplementary Figure 26 c, TC-6-1, 66 h。

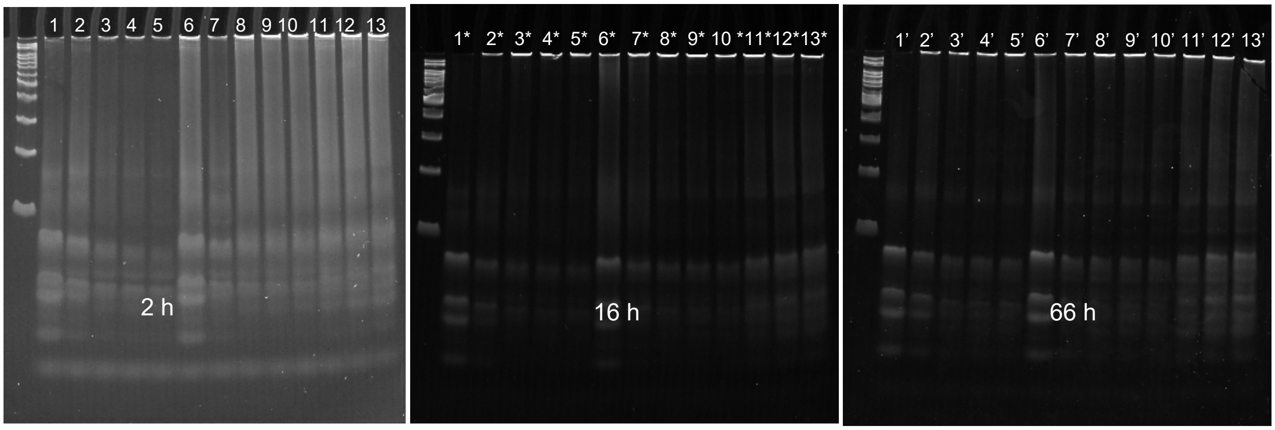

Supplementary Source Figure 13. Original gel images of Supplementary Figure 27c. Lane 10, Supplementary Figure 27c, TC-6-2, 2 h; Lane 10*, Supplementary Figure 27c, TC-6-2, 16 h; Lane 10’, Supplementary Figure 27c, TC-6-2, 66 h.

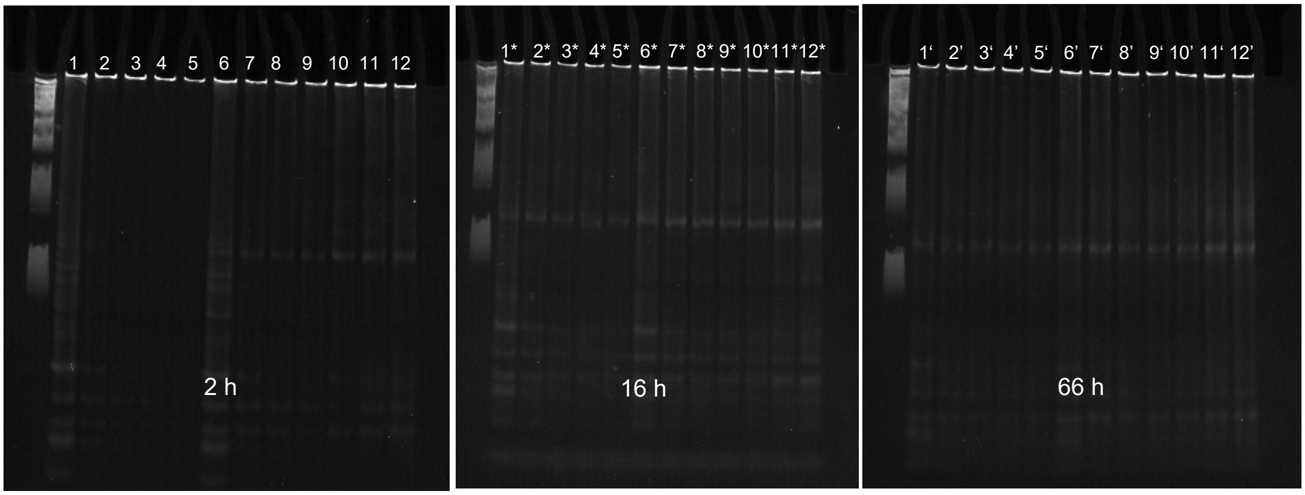

Supplementary Source Figure 14. Original gel images of Supplementary Figure 28c. Lane 11, Supplementary Figure 28c, TC-7-1, 2 h; Lane 11*, Supplementary Figure 28c, TC-7-1, 16 h; Lane 11’, Supplementary Figure 28c, TC-7-1, 66 h.

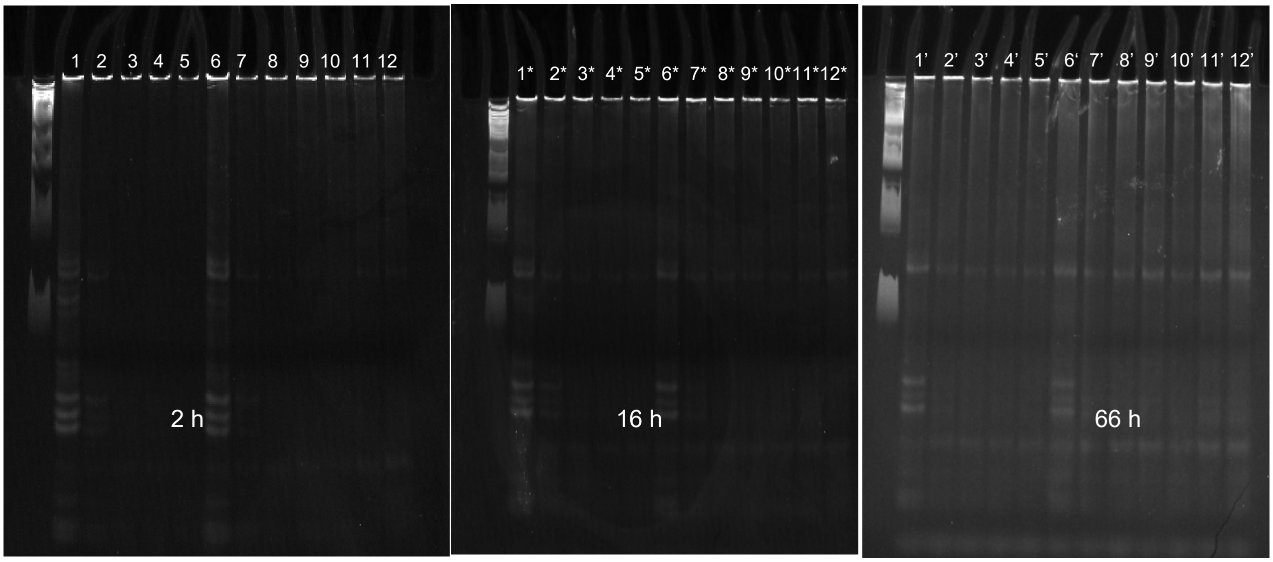

Supplementary Source Figure 15. Original gel images of Supplementary Figure 29c. Lane 10, Supplementary Figure 29c, TC-7-2, 2 h; Lane 10*, Supplementary Figure 29c, TC-7-2, 16 h; Lane 10’, Supplementary Figure 29c, TC-7-2, 66 h.

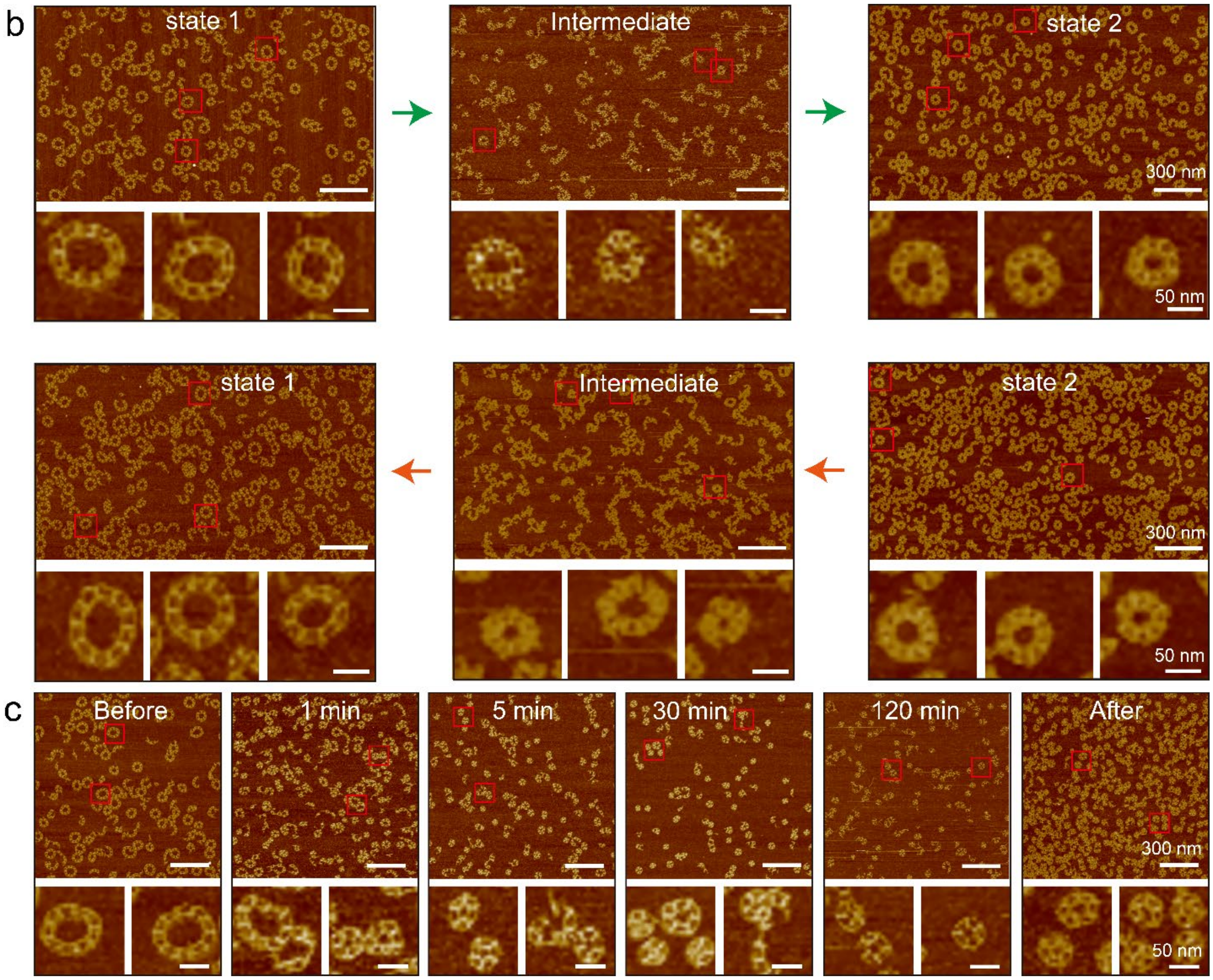

Supplementary Source Figure 16. The cropped and uncropped AFM images that are present in Supplementary Figure 35. The red squares indicate the cropped-out region.
