# Supplementary material for: DNA T-shaped crossover tiles for 2D tessellation and nanoring reconfiguration
Source: Nat Commun. 2023 Nov 23;14:7675. doi: 10.1038/s41467-023-43558-8 (PMC10667507; doi:10.1038/s41467-023-43558-8)
Supplement: Supplementary file 3 — Reporting Summary [file 41467_2023_43558_MOESM3_ESM.pdf]

nature portfolio

Corresponding author(s): Fei Zhang

Last updated by author(s): Oct 17, 2023

Reporting Summary

Nature Portfolio wishes to improve the reproducibility of the work that we publish. This form provides structure for consistency and transparency in reporting. For further information on Nature Portfolio policies, see our Editorial Policies and the Editorial Policy Checklist.

Statistics

For all statistical analyses, confirm that the following items are present in the figure legend, table legend, main text, or Methods section.

n/a	Confirmed	
☐	☒	The exact sample size (n) for each experimental group/condition, given as a discrete number and unit of measurement
☒	☐	A statement on whether measurements were taken from distinct samples or whether the same sample was measured repeatedly
☒	☐	The statistical test(s) used AND whether they are one- or two-sided<br>*Only common tests should be described solely by name; describe more complex techniques in the Methods section.*
☒	☐	A description of all covariates tested
☒	☐	A description of any assumptions or corrections, such as tests of normality and adjustment for multiple comparisons
☒	☐	A full description of the statistical parameters including central tendency (e.g. means) or other basic estimates (e.g. regression coefficient) AND variation (e.g. standard deviation) or associated estimates of uncertainty (e.g. confidence intervals)
☒	☐	For null hypothesis testing, the test statistic (e.g. F, t, r) with confidence intervals, effect sizes, degrees of freedom and P value noted<br>*Give P values as exact values whenever suitable.*
☒	☐	For Bayesian analysis, information on the choice of priors and Markov chain Monte Carlo settings
☒	☐	For hierarchical and complex designs, identification of the appropriate level for tests and full reporting of outcomes
☒	☐	Estimates of effect sizes (e.g. Cohen's d, Pearson's r), indicating how they were calculated

Our web collection on statistics for biologists contains articles on many of the points above.

Software and code

Policy information about availability of computer code

Data collection	No software was used.
Data analysis	No software was used.

For manuscripts utilizing custom algorithms or software that are central to the research but not yet described in published literature, software must be made available to editors and reviewers. We strongly encourage code deposition in a community repository (e.g. GitHub). See the Nature Portfolio guidelines for submitting code & software for further information.

Data

Policy information about availability of data

All manuscripts must include a data availability statement. This statement should provide the following information, where applicable:

- Accession codes, unique identifiers, or web links for publicly available datasets
- A description of any restrictions on data availability
- For clinical datasets or third party data, please ensure that the statement adheres to our policy

The authors declare that the data supporting the findings of this study are available within the paper and its Supplementary Information. Source data for all the statistical analysis of yield are provided with the paper. Raw data for figures with cropped AFM and gel images are supplied in Source Data files. Source data are provided with this paper.

Research involving human participants, their data, or biological material

Policy information about studies with human participants or human data. See also policy information about sex, gender (identity/presentation), and sexual orientation and race, ethnicity and racism.

Reporting on sex and gender	No human participant or biological materiel is involved in the research.
Reporting on race, ethnicity, or other socially relevant groupings	No human participant or biological materiel is involved in the research.
Population characteristics	No human participant or biological materiel is involved in the research.
Recruitment	No human participant or biological materiel is involved in the research.
Ethics oversight	No human participant or biological materiel is involved in the research.

Note that full information on the approval of the study protocol must also be provided in the manuscript.

Field-specific reporting

Please select the one below that is the best fit for your research. If you are not sure, read the appropriate sections before making your selection.

[x] Life sciences [ ] Behavioural & social sciences [ ] Ecological, evolutionary & environmental sciences

For a reference copy of the document with all sections, see nature.com/documents/nr-reporting-summary-flat.pdf

Life sciences study design

All studies must disclose on these points even when the disclosure is negative.

Sample size	For the yield counting, we used four 3 μm ×3 μm sized AFM images for each sample. These images for each sample contained at least 260 nanorings (more than 3000 DNA tile monomers). These AFM images for yield estimation are representative AFM images from many scans of at least three independent experiments. These AFM images with nanorings are sufficient for the estimation of the yield of well-formed structures because the nanoring samples are uniformly deposited on mica for AFM scanning.
Data exclusions	No data is excluded from the analyses.
Replication	All data are representative results of at least three independent experiments.
Randomization	No randomization is needed in this study because the samples are synthesized DNA nanostructures, which are self-assembled from DNA strands with designed sequences obtained from solid phase synthesis.
Blinding	Blinding is not relevant because the samples are synthesized DNA nanostructures, which are self-assembled from DNA strands with designed sequences obtained from solid phase synthesis.

Reporting for specific materials, systems and methods

We require information from authors about some types of materials, experimental systems and methods used in many studies. Here, indicate whether each material, system or method listed is relevant to your study. If you are not sure if a list item applies to your research, read the appropriate section before selecting a response.

Materials & experimental systems

n/a	Involved in the study	
[x]	[ ]	Antibodies
[x]	[ ]	Eukaryotic cell lines
[x]	[ ]	Palaeontology and archaeology
[x]	[ ]	Animals and other organisms
[x]	[ ]	Clinical data
[x]	[ ]	Dual use research of concern
[x]	[ ]	Plants

Methods

n/a	Involved in the study	
[x]	[ ]	ChIP-seq
[x]	[ ]	Flow cytometry
[x]	[ ]	MRI-based neuroimaging
