# Supplementary material for: DNA T-shaped crossover tiles for 2D tessellation and nanoring reconfiguration
Source: Nat Commun. 2023 Nov 23;14:7675. doi: 10.1038/s41467-023-43558-8 (PMC10667507; doi:10.1038/s41467-023-43558-8)
Supplement: Supplementary file 4 — source data [file 41467_2023_43558_MOESM4_ESM.zip › Source data/Source data-1 uncropped AFM images of Main Figures (Figure 2, Figure 3, Figure 4, Figure 5, and Figure 6).pdf]

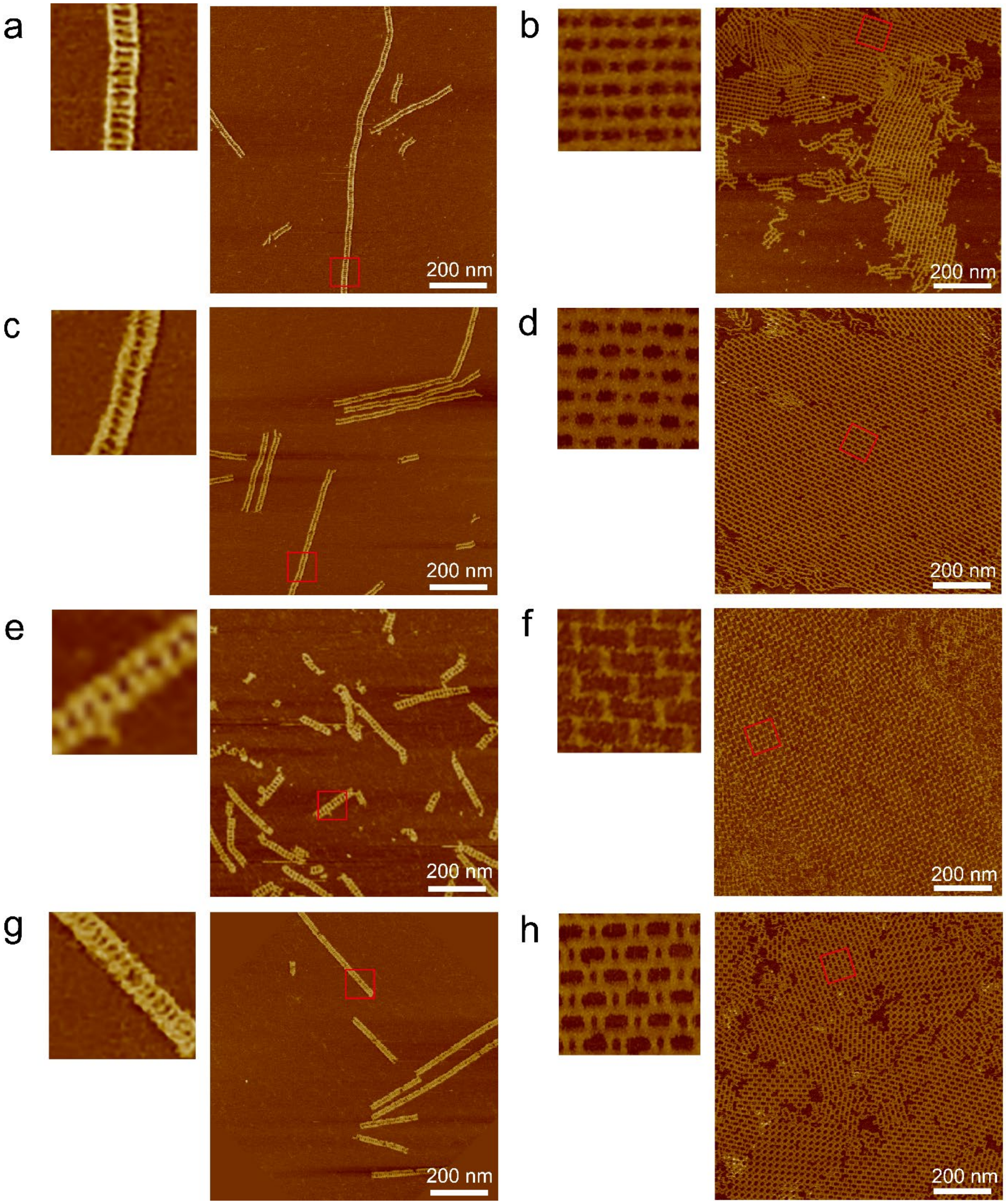

Source Figure 1. The cropped and uncropped AFM images that are present in Main Figure 2. The red squares indicate the cropped-out region.

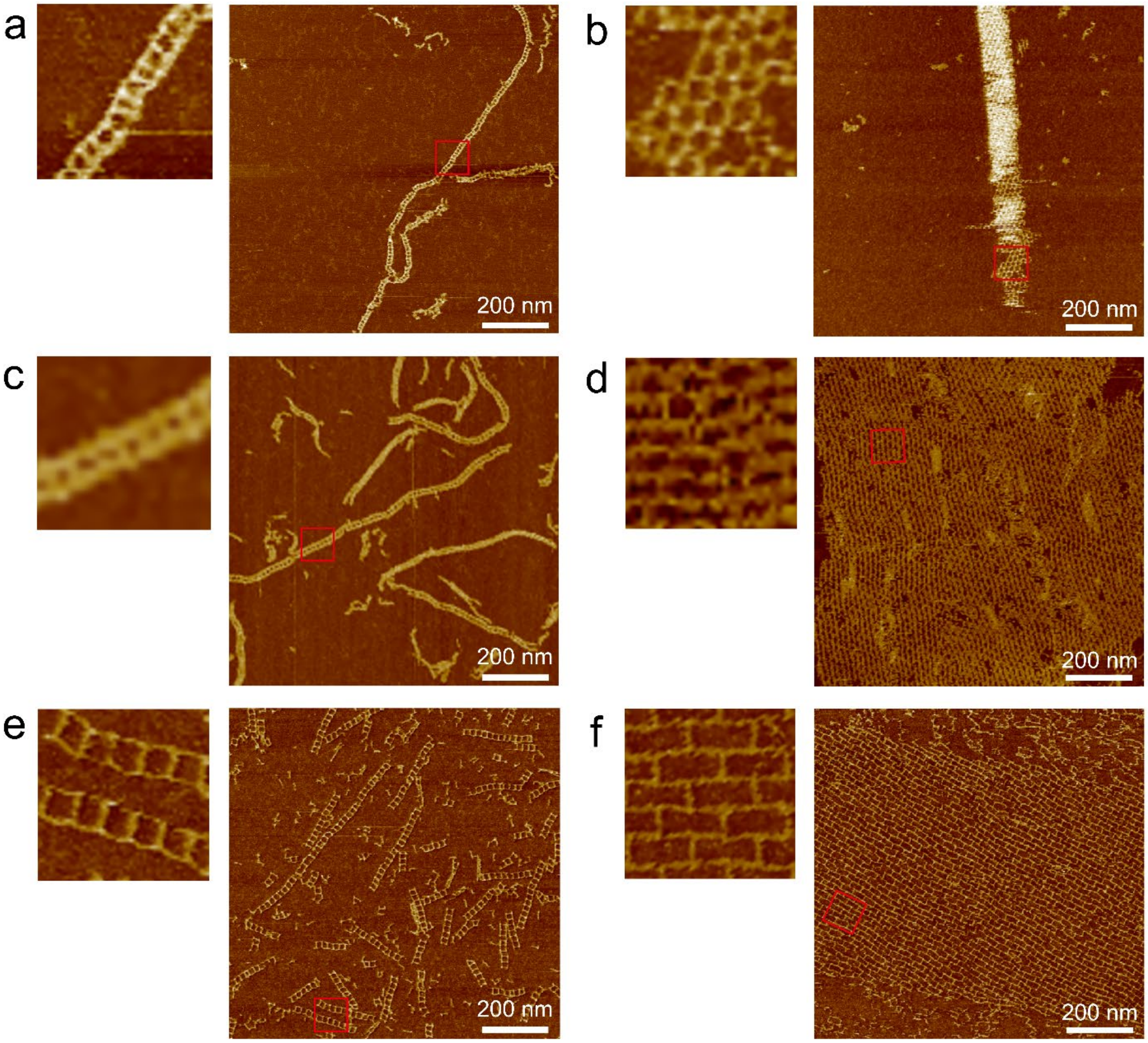

Source Figure 2. The cropped and uncropped AFM images that are present in Main Figure 3. The red squares indicate the cropped-out region.

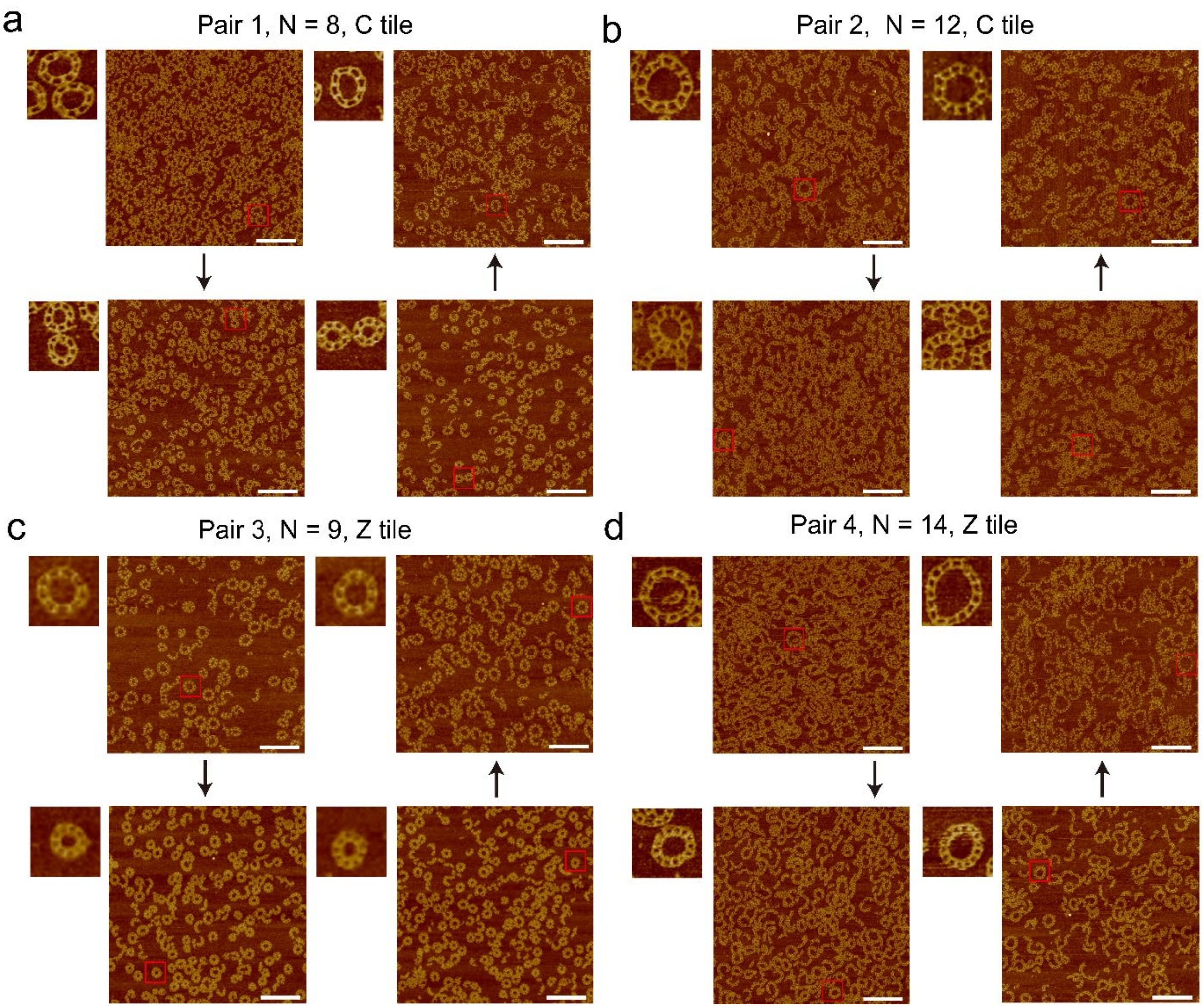

Source Figure 3. The cropped and uncropped AFM images are present in Main Figure 4. The red squares indicate the cropped-out region.

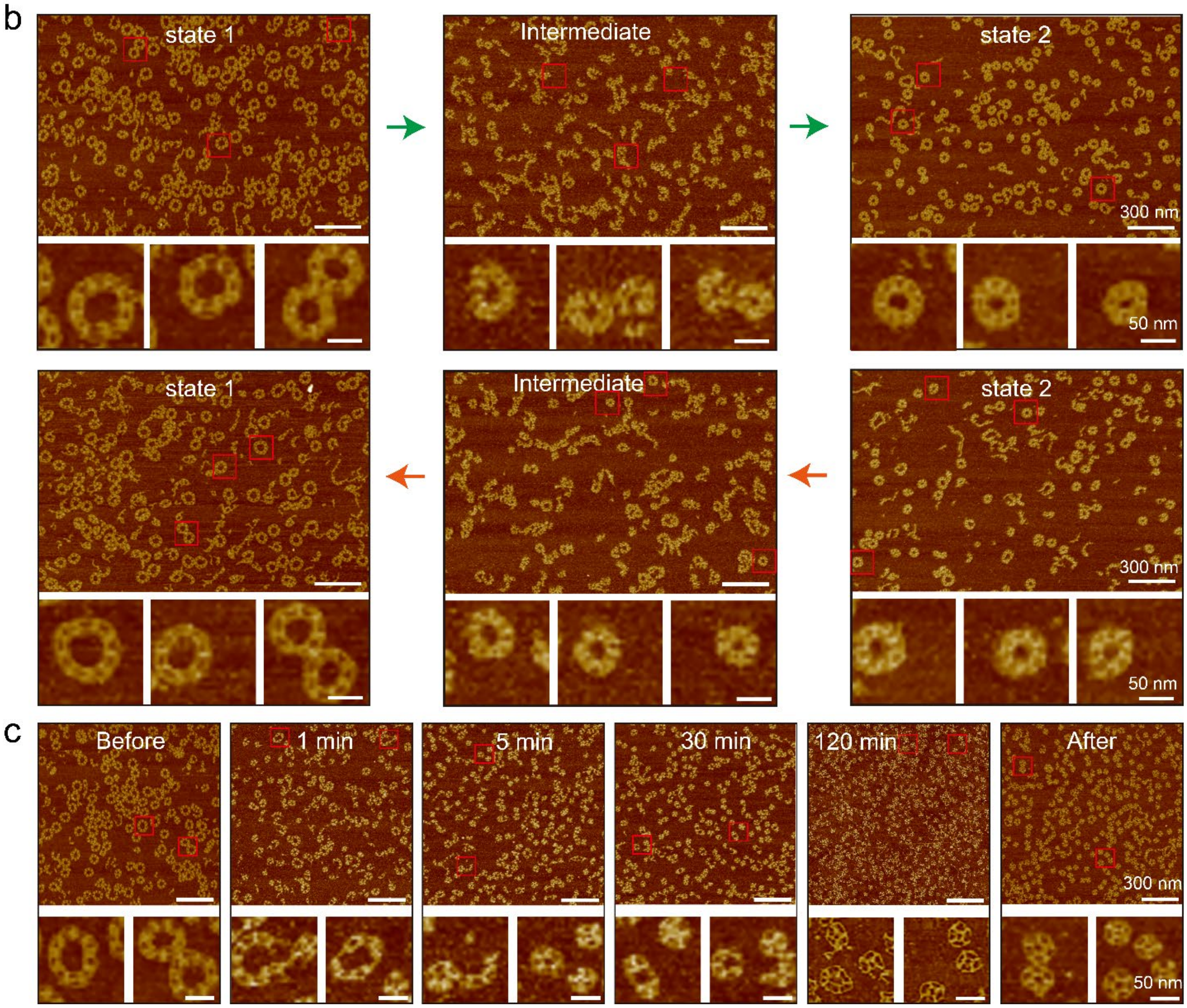

Source Figure 4. The cropped and uncropped AFM images that are present in Main Figure 5. The red squares indicate the cropped-out region.

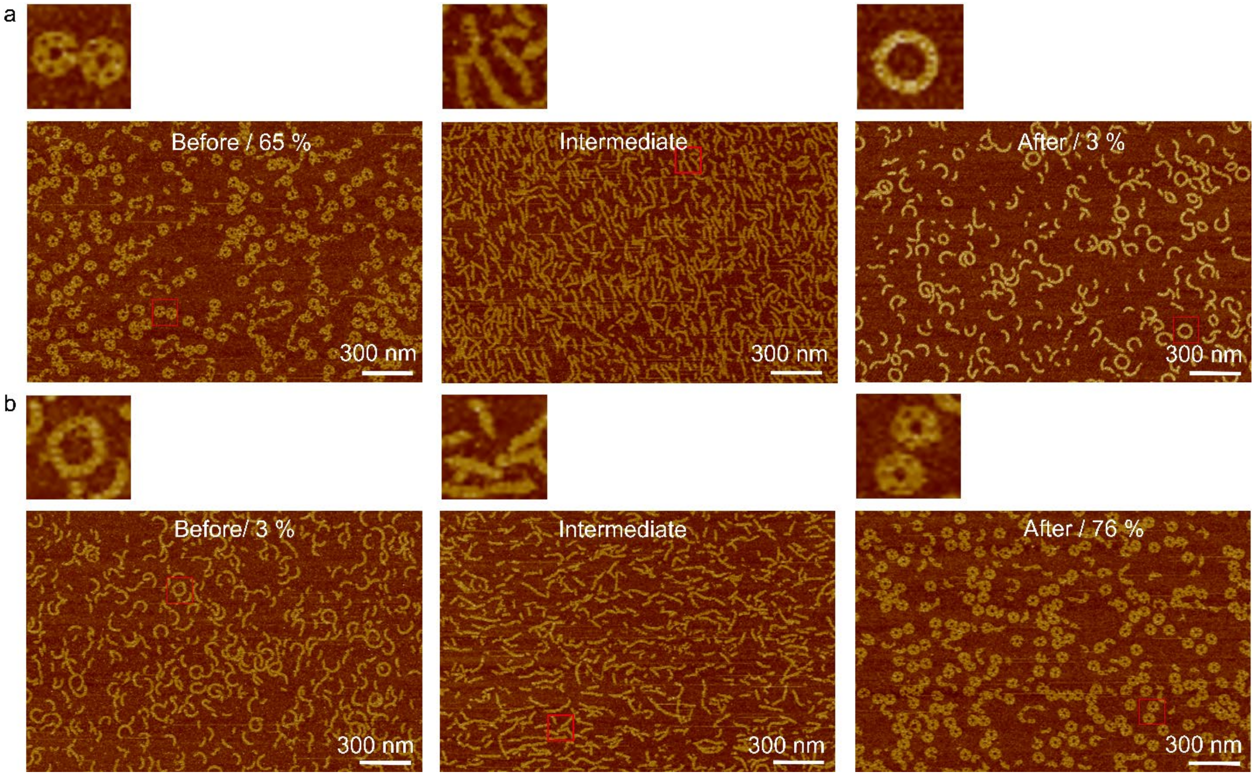

Source Figure 5. The cropped and uncropped AFM images that are present in Main Figure 6. The red squares indicate the cropped-out region.
